# Efficacy of an asynchronous telerehabilitation program in post-COVID-19 patients: A protocol for a pilot randomized controlled trial

**DOI:** 10.1371/journal.pone.0270766

**Published:** 2022-07-19

**Authors:** Beatriz Carpallo-Porcar, Laura Romo-Calvo, Sara Pérez-Palomares, Carolina Jiménez-Sánchez, Pablo Herrero, Natalia Brandín-de la Cruz, Sandra Calvo

**Affiliations:** 1 Department of Physical Therapy, Faculty of Health Sciences, Universidad San Jorge, Villanueva de Gállego, Zaragoza, Spain; 2 Hospital Real y Provincial Nuestra Señora de Gracia, Zaragoza, Spain; 3 Department of Physiatry and Nursing, Faculty of Health Sciences, IIS Aragon, University of Zaragoza, Zaragoza, Spain; Hong Kong Polytechnic University, HONG KONG

## Abstract

**Background:**

About 40% of patients who have had COVID-19 still have symptoms three months later whereas a 10% may experience physical and/or psychological consequences two years later. Therefore, it is necessary to perform preventive interventions when patients are discharged from the hospital to decrease the aforementioned sequelae. The purpose of this pilot-controlled trial will be to determine the efficacy of a rehabilitation program on functional status and psychosocial factors for post-COVID-19 patients when it is delivered through a tele-care platform versus a booklet-based rehabilitation.

**Methods:**

The estimated sample size will be of 50 participants who have been discharged after COVID-19 and have a level of fatigue equal or greater than 4 on the Fatigue Severity Scale. The primary outcome will be the severity of fatigue. Participants will be randomly allocated to an “asynchronous telerehabilitation group” or to a “booklet-based rehabilitation group”. Treatment in both groups will be the same and will consist of a combination of therapeutic exercise and an educative program. Treatment outcomes will be evaluated the last day of the intervention and at three- and six-months follow-up.

**Discussion:**

The telerehabilitation intervention appears to be a viable and efficacy option in decreasing severe fatigue and other fitness variables such as strength and aerobic capacity, similar to other traditional rehabilitation formats such as through an explanatory booklet.

**Clinical trial registration:**

This trial has been prospectively registered at clinialtrials.gov identifier: NCT04794036.

## Introduction

About 40%-70% of patients still have symptoms three months later whereas a 10% may experience physical and/or psychological consequences two years later [1–3]. In addition, it is known that patients who have been hospitalized or home-bound have greater physical consequences [4]. Therefore, these symptoms can be perpetuated over time and may lead to long-term consequences called “post-COVID condition” in approximately 10% of post-COVID-19 patients [2, 5].

Fever, cough, generalized pain, gastrointestinal disturbances, dyspnea, and fatigue are amongst the most frequent symptoms in the acute phase [6]. In the post-COVID-19 phase, when the infection has disappeared, the most common symptom was fatigue, which was present from 46 to 53% of the cases [1–3].

Furthermore, psychosocial factors such as the experience of illness and isolation, have entailed to higher levels of depression, stress and anxiety in post-COVID-19 patients affecting their health and recovery [7–10]. Both, physical and psychological sequelae affect quality of life, increasing the risk of comorbidities, and slowing down the recovery of these patients [11]. This might be very detrimental, especially in the elderly population [12, 13].

So, it is necessary to perform early interventions based on therapeutic exercise to prevent and improve all these sequelae after post-acute phase [4, 14–16]. This may lead to improvements in the functional status, which ultimately may reduce dyspnea, fatigue and other associated symptoms [17], even for patients with respiratory disease [18]. However, the health system overload due to the high number of people affected, the avoidance of face-to-face attendance, and the difficulty to provide health services due to the dispersion of the population, may limit the access of people to treatment after hospital discharge.

The use of telerehabilitation, defined as the delivery of rehabilitation services via technologies, appears to be a viable option [4, 13]. Furthermore, it could reduce the possible consequences of disease and prevent disability without the need to attend in person [12, 14, 19]. In addition, asynchronous telerehabilitation allows to incorporate home-based rehabilitation for the treatment of patients who have been discharged and do not require face-to-face treatment. This benefits especially to different population groups such as those who live in rural areas and has less access to health services, who experience any mobility limitations or have to be confined at their homes [20, 21].

According to the current scientific evidence, the efficacy of telerehabilitation has been demonstrated in many other pathologies such as the cardiac, neurological, respiratory, and musculoskeletal [19, 22–26]. There is already evidence of the efficacy of telerehabilitation interventions in the acute phase of COVID-19, at hospital setting patients and at home setting patients without hospitalization [27–30]. On the other hand, several studies have shown that telerehabilitation interventions can lead to an increased patient adherence and satisfaction [24, 31, 32]. To the authors’ knowledge, no previous studies have assessed if an asynchronous telerehabilitation program consisting of a combined therapeutic exercise and education program is effective to improve the functional status and the patient´s adherence and satisfaction of post-COVID-19 patients.

Therefore, the aim of this pilot study will be to analyze the efficacy on fatigue, functional status and psychosocial factors of a 12-weeks therapeutic exercise and education program, when this is delivered through an asynchronous telerehabilitation format versus a booklet-based rehabilitation format in post-COVID-19 patients.

## Methods

### Study design

**Fig 1:**
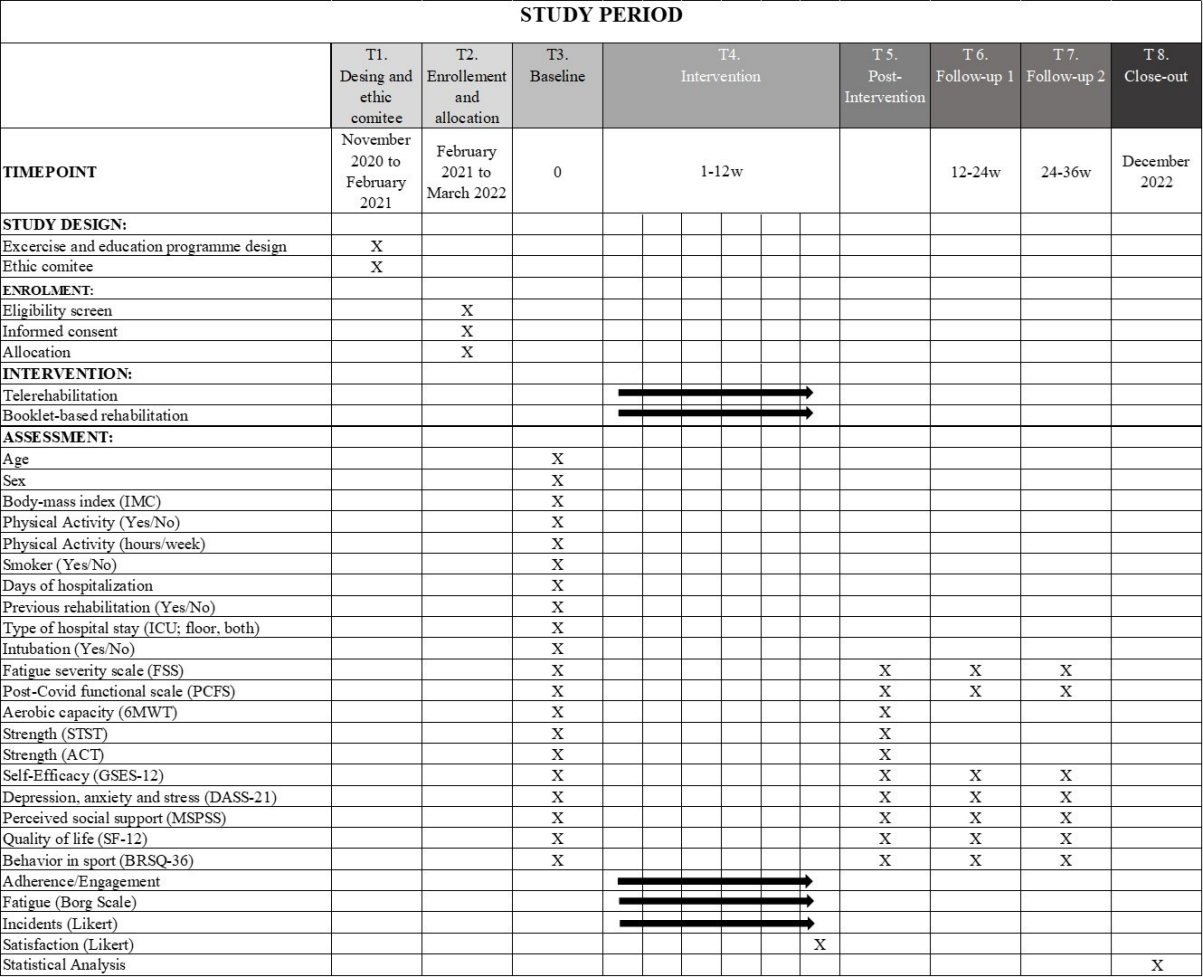
SPIRIT statement. Description of the study steps: 6MWT: 6 Meter Walk Test; STST: Sit-To-Stand Test; ACT: Arm Curl Test; GSES-12: General Self Efficacy Scale; DASS-21: Depression, Anxiety and Stress Scale; MSPSS: Multidimensional Scale of Perceived Social Support; SF-12: Self-reported Quality of Life Questionnaire-version 2; BRSQ-36: Behavioral Regulation in Sport Questionnaire.

**Fig 2:**
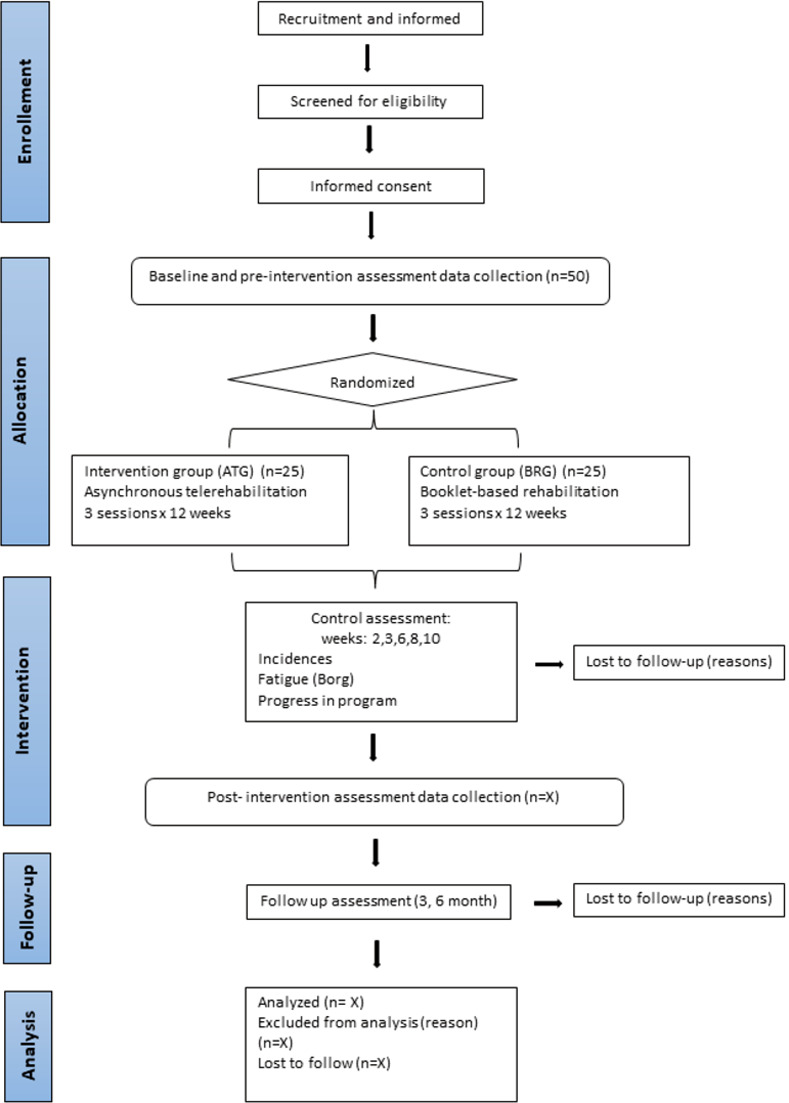
Flow chart.

This protocol will be a pilot and feasibility study with a single-blind randomized clinical trial design. The trial has been designed according to the Standard Protocol Items: Recommendations for Interventional Trials (SPIRIT) and will compare two parallel interventions: an intervention group (asynchronous telerehabilitation group, (ATG)) and a control group (booklet-based rehabilitation group, (BRG)). The duration of the study will be 12 months, with 12 weeks of intervention and two follow-ups at three- and six-months post-intervention. Figs 1 and 2 illustrate the pilot trial design. The study protocol has been approved by the Ethics Committee of Aragón (reference number: PI21/019, current protocol version date April 04, 2021). The protocol has been registered at clinialtrials.gov (NCT04794036).

### Setting and population

This study will be conducted at the Hospital Real y Provincial Nuestra Señora de Gracia (HPNSG) and the Hospital Royo Villanova (HRV), both in Zaragoza (Spain). Recruitment will be carried out at post-COVID-19 rehabilitation unit. Participants will be patients who had been infected by the COVID-19 and have been discharged from the hospital.

#### Eligibility criteria

All potential participants will be informed before and will have to give their consent to participate in the study. The inclusion criteria will be: 1) post-COVID-19 patients discharged after more than 5 days hospitalized; 2) aged 18 to 75 years; 3) independent standing with or without technical aids; and 4) present a degree of fatigue ≥4 points in the FSS. The exclusion criteria will be: 1) having any other central and/or peripheral neurological disorders; 2) a previous history of rheumatic pathology or acute musculoskeletal injury; 3) patients with severe hypoxemia, defined as having a SaO2 less than 90% or a respiratory rate ≥30; 4) having any cardiac comorbidities or signs of cardiovascular instability as uncontrolled arrythmia, blood pressure and/or effort angina; 5) having any other contraindicated pathology for moderate-intensity aerobic or strength exercise; 6) a score ≤24 evaluated with the validated Spanish version of Mini-mental State Examination (MMSE-MEC) [33]; 7) no access to internet; and 8) to be unable to follow oral and written instructions in the Spanish language.

#### Allocation and blinding

Participants will be assigned to the intervention or the control group through the software www.randomizer.org by an independent researcher, not involved in the treatment or evaluation, conducted at 1:1 ratio, giving each participant an identification code (IC) to guarantee their anonymity. The envelopes will be securely stored and will be opened in sequence to reveal group allocation.

Both, participants, and main researchers, will be masked to the randomization sequence. The evaluator will be unaware of the allocation and the intervention researcher will not participate in the evaluations.

### Procedure

At the time of hospital discharge all potential participants will receive the study information with a telephone number to contact. Those who agree to participate will call to the information number provided in the information sheet. One of the researchers of the study will ensure that the patient meets the inclusion and exclusion criteria and will schedule the patient for the baseline and pre-intervention assessment. In the first meeting with the researcher, the patient will be asked to sign the informed consent form.

All assessments will be always performed at the same time and place in the hospital to maximally preserve the participant conditions. Each assessment will last about 60 min. This evaluation shall be carried out according to the same procedure previously trained.

Once the baseline and pre-intervention evaluation is completed, a second researcher will assign each patient to the ATG or BRG according to the 1:1 randomization previously done with a web page (www.randomizer.org). This same researcher will help the ATG patients to install the telerehabilitation platform in the mobile (through an app) or will explain the home booklet-based program to the BRG patients. The intervention will be prescribed considering the starting level of the program which will depend on the pre-intervention FSS score.

Every two weeks during the intervention, all patients will receive a control call from the second researcher to ask them about possible incidents and decide if the patient must progress to the next exercise phase based on the intensity of fatigue perceived by each patient. Three- and six- months after the intervention, patients will receive a follow-up call to analyze the level of fatigue (FSS), the functional status (PCFS), the quality of life (SF-12) and the psychosocial factors (GSES-12, DASS-21, BRSQ-36, MSPSS) (Figs 1 and 2).

### Intervention

The ATG and BRG will receive the same therapeutic exercise and education program. It has been designed following the recommendations of the Spanish physiotherapy associations, the Spanish Society of Pneumology and Thoracic Surgery (SEPAR) and the American Thoracic Society (ATS) [34–36]. The program will be performed for 12 weeks (three sessions/week). The therapeutic exercise program will include aerobic, strength and lung capacity exercises [37]. It will be composed of three levels of difficulty with different exercises in each one, except for the pulmonary exercises (S1 Appendix). The first level will include patients with a score equal to or greater than 6 on the FSS, the second level will include those with a score between 5–5.99 and the third one will include those patients with a score between 4–4.99. Every patient, regardless of their initial level, will start with 10 minutes of the proposed aerobic exercise and 3 sets of strength exercises doing as many repetitions as the patient can. The lung capacity exercises will be the same throughout the entire intervention and will be performed in 2 sets of 10 repetitions with a fatigue level below 3 on the Borg Scale.

The intensity will be measured with the modified Borg Scale. All participants will start for the first two weeks with a fatigue intensity between 3 and 4 and, after 2 weeks, the intensity will be increasing to levels between 5–7 level. Patients will progress every two weeks according to the information provided during the control calls. The appearance of symptoms such as chest pain, lot of coughing, high fatigue, fever, dizziness, or any others deemed relevant, will be taken into account not to progress with the exercises.

The therapeutic education program will be available to all participants during the 12-week intervention. It will consist of 3 blocks of health advice. The first block will focus on how to prevent reinfection by COVID-19 though hygienic recommendations. The second block will focus on how to plan daily life when the patient is at home for a longer period and the third block will focus on psychological advice to reduce levels of anxiety and stress during the recovery period.

#### Intervention group

**Fig 3:**
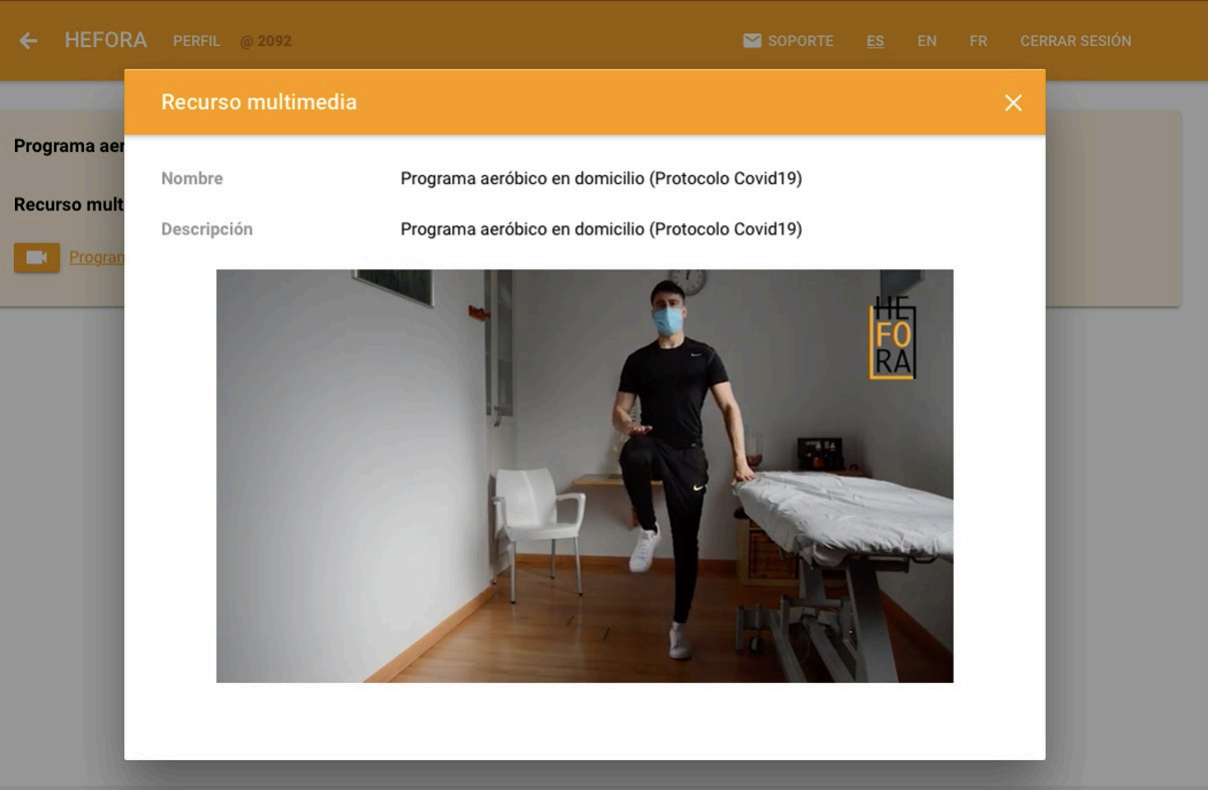
HEFORA interface.

The ATG will perform the program through a telerehabilitation platform which is accessible via www.hefora.net or with an app mobile that will be installed in the patients´ mobile (HEFORA, Fisio Consultores, Zaragoza, Spain). Therapeutic exercises will be presented in the form of an explanatory video with a specific description (Fig 3). The platform allows the physical therapist to customize the number of sets, repetitions, speed, and observations for each patient. The therapeutic education recommendations will be presented through animated educational videos, explaining to the patients the health and emotional tips to improve their quality of life after COVID-19.

#### Control group

The BRG will perform the same therapeutic exercise and education program than the ATG but through a booklet-based rehabilitation, which will include key pictures and descriptions for every exercise of each level. In addition, patients in the BRG will receive the same therapeutic education recommendations that in the ATG but in text format.

### Outcome measures

#### Primary outcome: Fatigue

The primary outcome measure will be the fatigue, measured with the Fatigue Severity Scale (FSS). The FSS is a self-reported scale that allows assessing the severity of fatigue as a sense of physical tiredness, muscle weakness and lack of energy [38–40]. It is composed of 9 items with scores ranging from 1 = strongly disagree to 7 = strongly agree. The higher the number, the greater the severity of fatigue. The most common cut-off point is a mean score of 4 point, considering equal or more than 4 as severe fatigue [38, 41–45]. Three levels of severity will be considered: 4 = borderline fatigue; 5 = high fatigue; 6–7 = very high fatigue [44]. It has been shown to be reliable [38].

#### Secondary outcomes: Functional status, psychosocial factors, quality of live and feasibility

*Functional status*. It will be measured using the main recommended test for post-COVID-19 patients [46].

Aerobic capacity
  - 6MWT is a sub-maximal exercise test used and recommended to assess the maximum distance possible for six minutes, in a 30-meter corridor, allowing the patient to rest as needed. It has been shown to be reliable [47–49]. The distance covered by each participant will be compared with the estimated distance for their gender, weight, and age according to the Troosters equation [50–52].
Strength
  - 30” STST is part of the Senior Fitness Test (SFT) designed by Rikli and Jones and it will be used as a stand-alone test, especially to assess weakness in respiratory patients who have passed COVID-19 [53–55]. It has been shown to be reliable in adults with asthma or pulmonary hypertension [53, 56–58]. This test will be performed with a chair, with the patient´s feet resting on the floor and the arms crossed on the chest. Once in this position, the patients will sit down and stand up to the starting position as many times as possible within 30 seconds [59].
  - 30” ACT is part of the SFT and is also used as a stand-alone test to assess strength. It has been shown to be reliable [53, 60, 61] in deconditioned patients and in the elderly population. From a sitting position, the patient is asked to do as many elbow flexion-extension movements as possible with his/her dominant limb during 30 seconds with a two kg weight. The higher number of repetitions the better strength [53, 59].
Functional Status post-COVID
  - PCFS is a scale specially designed and that has been shown to be reliable to assess the functional status of patients who have gone through COVID-19 at discharge [50, 62–64]. It consists of four levels of post-COVID-19 functional limitations been: D = death; 0 = no limitation; 1 = no significant functional limitation; 2 = mild functional limitation; 3 = moderate functional limitation; 4 = severe functional limitation. This scale can be administered by telephone or self-administered [12].

*Psychosocial factors*.

- Self-efficacy will be measured with the General Self-Efficacy Scale (GSES-12), which is an abbreviated scale developed by Bosscher and Smit [65]. It is composed of 10 items scored on a Likert scale from 1 = never happens to me, to 5 = always happens to me. It has been shown to be reliable [66]. The higher the score, the greater the perceived self-efficacy [65, 67–70].
- The level of depression, anxiety and stress will be measured with the Depression, Anxiety, and Stress Scale (DASS-21), which is a set of 3 self-reported questionnaires designed to measure the negative emotional states of depression, anxiety, and stress. Each of these DASS subscales contains 7 Likert-type items with a score from 0 = did not apply to me, to 3 = applied to me very much or most of the time. The total score for the global scale score ranges from 0 to 21 points. The higher scores indicate higher levels of stress, depression, and anxiety [71–73]. The DASS-21 has been shown to be reliable [74, 75].
- The Behavioral Regulation in Sport Questionnaire (BRSQ-36) will be used to measure the motivation to practice physical activity. It has been shown to be reliable [76, 77]. The BRSQ-36 is composed of 36 items, and it is divided in three main domains: extrinsic motivation: integrated regulation (items 5,14,23,32), identified regulation (items 6,15,24,33), introjected regulation (items 7,16,25,34) and external regulation (items 8,17,26,35); intrinsic motivation: knowledge (items 2,11,20,29), performance (items 4,13,22,31), stimulation (items 3,12,21,30) and general (items 1,10,19,28) and 3); and amotivation (items 9,18,27,36). Its items are valued using a Likert scale that ranges from 1 = not at all true, to 7 = very true. A higher total score on each of the factors indicates a higher dominance of that motivational factor [78].
- The perceived social support will be measured with the Multidimensional Scale of Perceived Social Support (MSPSS) [79]. It is considered a priority scale to be applied in people who are in the process of recovery. It has been translated and validated in different languages [80]. It measures social support in 3 domains, family, friends, and significant others. It has a three factors structure with each subscale comprising four items addressing practical help. Emotional support, availability to discuss problems and help in decision making. The version used will consist of 12 items that are answered using a Likert scale that ranges from 1 = totally disagree, to 7 = totally agree. Total scores from 12–14 indicate low social support, scores from 49 to 68 indicate moderate social support, and scores from 69 to 84 indicate high social support [81, 82].

*Quality of life*. It will be measured with the Self-Reported Quality of Life questionnaire, version 2 (SF-12). The SF-12 is a shortened version of the SF-36 conducted in 2002. It consists of 12 questions covering 8 dimensions which are grouped into two components, physical and mental. The scores for each item are different and range from 1–2 to 1–6. The overall score ranges from 0 to 100 considering both components. Its interpretation is based on the reference values for the Spanish population, indicating higher scores a higher self-perceived quality of life [83–85]. The version 2 of SF-12 has been shown to be reliable in a student population during COVID-19 [86, 87].

*Feasibility*. It will be measured by the recruitment and adherence rate, the report of satisfaction and the number of incidences. It will be reported with a diary in the telerehabilitation platform or in a diary in the booklet [88, 89].

- Adherence is the degree to which a person’s behavior, with respect to taking medication or lifestyle change recommendations, is in accordance with the prescriptions of the healthcare professional. It will be recorded in the activity logs of the brochure or through the platform questionnaire and will be coded by means of a Likert scale, with 0 = if do not has performed the exercises, 1 = if has have carried out some exercises, 2 = if has performed all recommended exercise, and 3 = if they have carried out more additional activity. Additional physical activity will be understood as voluntary physical activity that exceeds the recommended daily activity. A high adherence will be considered when the participant performs at least the 80% of the sessions, and they have done all recommended exercised or more. Non-adhesion will be considered to perform less than 20% of the sessions [90, 91].
- Recruitment rate, will be calculated based on the percentage of the sample size recruited.
- Satisfaction with the pilot process will be recorded with a predetermined questionnaire on a Likert scale, from 0 = very dissatisfied, to 4 = very satisfied [92–94]. Participants will evaluate the overall aspects, the brochure, the platform, the attention received, and the exercises given.
- Incidents will be assessed during the control calls: 0 = no incidences; 1 = some incidence but it has been solved; 2 = incidences that makes it impossible to follow up that period.

**Fig 4:**
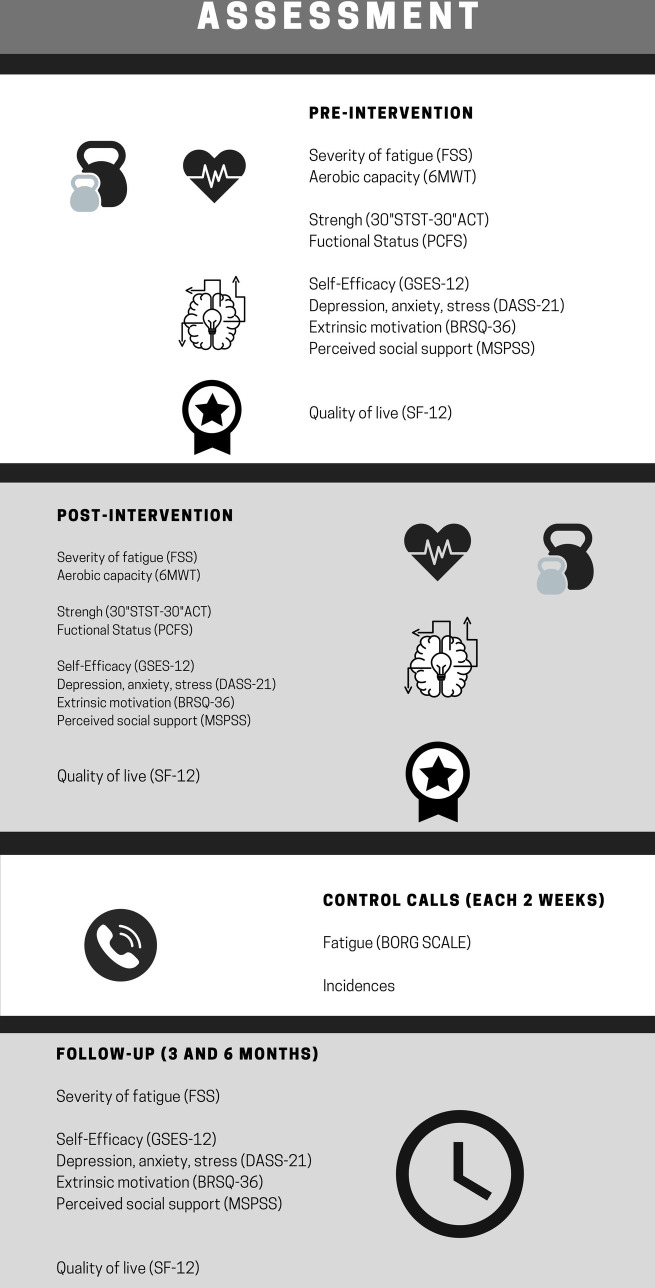
Timeline of outcome measurement. FSS: Fatigue Severity Scale; 6MWT: 6 Meters Walk Test; STST; Sit-To-Stand Test; ACT: Arm Curl Test; PCFS: Post-Covid Functional Scale; GSES: General Self Efficacy Scale; DASS: Depression, Anxiety and Stress Scale; BRSQ: Behavioral Regulation in Sport Questionnaire; MSPSS: Multidimensional Scale of Perceived Social Support.

All the clinical outcomes (FSS, PCFS, GSES-12, DASS-21, BRSQ-36, MSPSS, SF-12) will be measured at pre-intervention, post-intervention and at three- and six-months follow-up. The functional status outcomes (6MWT, 30” ACT and 30” STST) only will be measured pre- and post-intervention. Regarding the feasibility outcomes, incidences and adherence will be collected during the control calls every two weeks. Satisfaction will be measured in the post-intervention evaluation. The timeline is shown in Fig 4.

#### Sample size calculation

A sample size of 50 subjects will be required, 20 in each group, plus 10 in anticipation of future losses according to the recommendations for RCT pilot studies [95, 96].

#### Data management

A detailed database will be set up to track each participant’s progress, including all evaluations. All the generated data will be recorded and stored on password-protected computers, that will be only accessible to the researchers involved in this study. An independent researcher will monitor data collection progress and safety. Data will be analyzed when all recruitment and data collection has been performed.

#### Statistical analysis

Statistical analysis will be performed with Statistical Package for the Social Sciences version 25.0 (SPSS Inc, Chicago, IL). The significance level will be 0.05 for all statistical analyses. Descriptive statistics, including frequency counts for categorical variables and measurements of central tendency and dispersion for continuous variables (standard deviation, 95% confidence interval) will be calculated to summarize the data.

The Shapiro-Wilk test will be used to determine the normality of the data.

General lineal models will be performed to compare the intervention effects [time (pre-intervention vs post-intervention vs follow-up 1 and 2 assessments) x group interaction] on the primary and secondary outcomes: severity of fatigue, functional status, self-efficacy, depression, anxiety and stress, quality of live, behavioral regulation in sport and perceived social support. It will be examined with analysis of variance (ANOVA) mixed model for repeated measures when normal distribution will be detected. A post hoc test will be used to locate the differences between the groups, if necessary. If data are not normally distributed, statistical analysis will be performed using non-parametric correspondent tests like Friedman test to compare the three moments involved and Wilcoxon test if statistics significance will be reached in previous Friedman test. In Wilcoxon test, type I error will be divided by the number of tests done.

For the aerobic capacity, strength, functional status post-COVID and feasibility outcomes, comparisons between-groups outcomes will be using t-tests for independent samples and Levene test for parametric data, or Mann-Whitney test U tests for non-parametric data. Chi-square and Fisher tests will be used of independence for categorical data. Finally, in the event of possible dropouts, analyses will be conducted as intention to treat.

Between- and within-group effect sizes using Cohen’s d or r for main outcomes will also be computed.

Chi square test for qualitative data and t-test or Mann-Whitney for normal or not normal data respectively will be used to check the comparability between the two groups at baseline.

#### Ethical aspects and dissemination

The study will be conducted following the ethical guidelines of the Declaration of Helsinki. This protocol has been approved by the Ethics Committee of Aragón (reference number: PI21/019, current protocol version date April 04, 2021). This research was registered at ClinicalTrials.gov Protocol Registration System with the number NCT04794036.

Only the researcher that will be performed the analysis will have access to the data set. To ensure confidentiality, data will be codified to blinded of any identifying participant information.

## Discussion

This protocol describes a pilot and feasibility study to analyze the efficacy of an exercise and education therapeutic program using asynchronous telerehabilitation for post-COVID-19 patients. The aim of the pilot study will be to analyze if there are improvements in the participants´ level of fatigue and if these changes are maintained in the short (3 months) and medium term (6 months) and compare it with traditional home rehabilitation using a booklet. The secondary aim will be to analyze the feasibility of this pilot study.

Telerehabilitation is well accepted in terms of overall user experience, adherence, and satisfaction [94]. So, it represents a great opportunity for the treatment and monitoring of long-term patients. Currently, the large increase of COVID-19 cases during 2020 and 2021 has made telerehabilitation-based consultation a viable and reliable option in several pathologies. Scientific evidence shows how telerehabilitation is already effective in post-COVID-19 patients, as reported in the systematic review of da Silva et al. [97] where it was found that a physical programme using telerehabilitation can improve functional capacity, dyspnea and quality of life. Similarly, Dalbosco-Salas et al. [98], found that a group of post-COVID-19 patients undergo remote rehabilitation using synchronous telerehabilitation, achieved improvements in physical condition and quality of life. Although their model is on-line and there is no control group, the study shows positive results for post-COVID-19 patients in lower limb strength and quality of life. Furthermore, in most of the studies carried out in the acute and post-COVID-19 patients, the control group does not receive treatment.

Moreover, telerehabilitation has also shown to be effective in many other different conditions such as musculoskeletal pain, chronic obstructive pulmonary disease (COPD) or cardiovascular disease [99]. The study of Lewis et al. [100] showed that telerehabilitation is effective in COPD patients on multiple variables such as lower limb strength, fatigue, dyspnea, and anxiety, including improvements also in patient engagement and exercise progression. Similar results were obtained by Batalik et al. [101], who performed a study with asynchronous telerehabilitation in cardiac patients and showed also to be effective to improve the aerobic capacity assessed with the 200 meter fast-walk test.

When telerehabilitation is compared to other formats, most of the studies about telerehabilitation reported clinical improvements in favor of telerehabilitation, but no statistically significant changes compared to other ways, which leads us to expect similar results in our study. Suso-Martí et al. [102] in their systematic review with meta-analysis, showed how telerehabilitation generated clinical improvements in physical condition variables in cardiorespiratory, musculoskeletal, and neurological patients, even comparable to conventional face-to-face rehabilitation. Other studies such as Dias et al. or Hansen et al. [103, 104] reported how telerehabilitation was not different to other interventions for physical function. The systematic review of Dias et al. [103] concluded that exercise via telerehabilitation can be an alternative to improve physical function and quality of live in persons with disabilities. Moreover, Hansen et al. [104] also found how telerehabilitation was similar to face-to-face rehabilitation in severe COPD patients to improve the aerobic capacity and the lower limb strength.

Regarding the psychosocial factors, several trials, such as ours, have implemented in their protocols a therapeutic education program together with the exercise program, with the aim of improving the emotional components by telerehabilitation [105]. These interventions seem to show better results than those based on isolated therapeutic exercise [103, 106]. However, although it seems that emotional factors can be improved with therapeutic education, it has no effect on decreasing the perception of fatigue [107]. In the cross-sectional study by Milani et al. [108] in adult population with a physical disability, it was observed that those patients who were able to undergo synchronous telerehabilitation via Skype had higher self-efficacy with respect to the control group. In addition, Hansen et al. [104] demonstrated improvements in the depression scale, compared to the group without telerehabilitation, measured with the HADS-D scale.

Finally, we also expect to find clinical changes in the quality of life, especially in the physical component, as was found in the study by Milani et al. [108] where a slight improvement was observed in the telerehabilitation group. Likewise, the systematic review by Dias et al. [103] showed that telerehabilitation is similar in efficacy in terms of improving quality of life, assessed with the SF-36 scale, to other systems in both the short and long term.

### Strengths and weaknesses of the study

The main limitation is that the two modalities of the program make it impossible to blind the patients. To avoid this intervention bias, all participants will be told that both programs are the same and with the same personalized control-calls, stressing that the only difference is the format. Other limitation will be that in the home booklet-based program will be not possible to control the execution of the exercises of each patient. Although there is a diary to record the activity on the platform and in the booklet, in order to reduce patient memory bias, adherence will be monitored during the control calls to control the dates when patients have not recorded their activity.

## Supporting information

S1 Checklist Checklist SPIRIT protocol. (DOCX)

S1 File Project submitted to the ethics committee (English). (PDF)

S2 File Project submitted to the ethics committee (Original Spanish). (PDF)

S3 File Opinion ethics committee (English). (PDF)

S4 File Opinion ethics committee (Original Spanish). (PDF)

S1 Appendix (DOCX)
