# Supplementary material for: Efficacy of an asynchronous telerehabilitation program in post-COVID-19 patients: A protocol for a pilot randomized controlled trial
Source: PLoS One. 2022 Jul 19;17(7):e0270766. doi: 10.1371/journal.pone.0270766 (PMC9295945; doi:10.1371/journal.pone.0270766)
Supplement: S1 File — (PDF) [file pone.0270766.s002.pdf]

PROJECT MEMORY

Efficacy of an asynchronous telerehabilitation programme in post-COVID-19 patients: a feasibility study.

INDEX

TITLE 3

RESEARCH TEAM 3

1.- BACKGROUND/JUSTIFICATION 4

2.- HYPOTHESIS 6

2.1.- HYPOTHESIS 7

OBJECTIVES 7

3.1.- GENERAL OBJECTIVE 7

3.2.- SPECIFIC OBJECTIVES 7

4.- METHODOLOGY 8

4.1.- DESIGN 8

4.2.- STUDY SUBJECTS 9

4.2.1.- Inclusion Criteria 10

4.2.2.- Exclusion Criteria 11

4.2.3.- Randomisation 11

4.3.- INTERVENTION 12

4.3.1.- Intervention protocol. 12

Experimental group 14

4.3.3.- Control group 15

4.4.- VARIABLES 16

4.4.1.-Main variable pre-observational phase: needs identification 16

4.4.2.-Main clinical trial variable: fatigue level 17

4.4.3.-Secondary Variables 18

4.5.- SAMPLE SIZE 22

4.6.- STATISTICAL ANALYSIS 22

4.7.- BIASES AND LIMITATIONS 23

5.- ETHICAL ASPECTS 23

5.1.- INFORMED CONSENT 23

5.2.- RISKS AND CONTINGENCIES 24

5.3.- DATA PROTECTION 24

5.4.- CARE IMPLICATIONS 28

6.- TIMETABLE 28

7.- ANNEXES 30

8.- BIBLIOGRAPHY 72

TITLE

Efficacy of an asynchronous telerehabilitation programme in post-COVID-19 patients: a feasibility study.

RESEARCH TEAM

- Sandra Calvo Carrión. Physiotherapist. Lecturer in the Degree in Physiotherapy. iPhysio Research Group. Faculty of Health Sciences. San Jorge University. Zaragoza.
- Carolina Jiménez Sánchez. Physiotherapist. Lecturer in the Degree in Physiotherapy. iPhysio Research Group. Faculty of Health Sciences. San Jorge University. Zaragoza.
- Sara Pérez Palomares. Physiotherapist. Lecturer in the Degree in Physiotherapy. iPhysio Research Group. Faculty of Health Sciences. San Jorge University. Zaragoza.
- Beatriz Carpallo Porcar. Physiotherapist. iPhysio Research Group. Faculty of Health Sciences. San Jorge University. Zaragoza.
- Laura Romo Calvo. Physiotherapist, Nuestra Señora de Gracia Provincial Hospital. Zaragoza.
- Pablo Herrero Gallego. Physiotherapist and researcher. Assistant Professor Doctor University of Zaragoza. Zaragoza.
- Natalia Brandín de la Cruz. Physiotherapist. Lecturer in the Degree in Physiotherapy. iPhysio Research Group. Faculty of Health Sciences. San Jorge University. Zaragoza.
- Dr. Jorge Alamillo Salas. Rehabilitation Physician Royo Villanova Hospital.
- Irene Liñares Valera. Degree in Psychology from the University of Santiago de Compostela. Lecturer in Physiotherapy Degree. Faculty of Health Sciences. San Jorge University. Zaragoza.
- Laura Bafaluy Franch. Degree in Psychology from the University of the Basque Country (San Sebastian). Lecturer on the Degree in Physiotherapy. Faculty of Health Sciences. San Jorge University. Zaragoza

1.- BACKGROUND/JUSTIFICATION

SARS-CoV-2 is the coronavirus responsible for developing the disease known as COVID-19. Its evolution can range from an asymptomatic course, to rapidly evolve and cause an acute[1] respiratory syndrome. In addition to respiratory symptoms, it also has an impact on the neuromuscular and cardiovascular[2,3] systems. According to December

2020 data from the Spanish Ministry of Health, since the appearance of SARS-CoV-2 in Wuhan (China) in December 2020, more than 46,000 people 2019, have died and more than 1,628,200 have been infected[2] in Spain. With a mortality rate in hospitalised patients of 21% according to the Spanish Society of Health Managers (SEDISA)[4]. The international Public Health emergency was declared by the World Health Organisation (WHO)[3] on 30 January 2020 and the classification of pandemic on 11 March 2020. 2020(5).

Since then, Spain has become the second European country in terms of the number of deaths per million inhabitants and more people 16.000have been admitted to hospital[2]. In Aragon, more than 72,500 cases have been confirmed and more than 8,200 people have been hospitalised, including those in Intensive Care 730Units (ICU), with a total of 2,280 deaths up to 6 December 2020 according to data from the Government of Aragón[6].

Patients with COVID-19 typically present with a variety of symptoms: fever, cough, generalised pain, gastrointestinal disturbances, and dyspnoea and fatigue, the latter two symptoms occurring in about half of these patients. The prevalence of musculoskeletal symptoms in COID-19 patients worldwide is greater than 50% of the world population, with the highest prevalence reported in Europe[7]. In Spain, among hospitalised cases, pneumonia appears in about 80% of cases[4], which increases the prevalence of dyspnoea and fatigue in this group of patients[1,9].[8,] These consequences of the disease are aggravated by the time they spend confined to their homes or admitted to hospital. The estimated time from onset of illness to disappearance of symptoms is 2 weeks for mild disease and 3-8 weeks on average for severe[10] disease. Therefore, this additional inactivity has a negative influence on the loss of muscular, cardiovascular and metabolic fitness among other systems.

During phase 3 of the epidemic, less urgent[(11)] inpatient rehabilitation treatments were suspended in Spain and the rest of Europe, as recommended by the World Confederation for Physical Therapy for safety[(12)] reasons. Due to this health emergency situation, current guidelines only recommend rehabilitation for severe COVID-19 patients or those with a high risk of comorbidities[(13)], until the health situation improves or the pressure of care decreases.

Partly as a result, a large number of patients currently have sequelae; about 40% of those discharged from hospital still have symptoms[(5)]. It is important to note that in Spain, 61% of new admissions for sequelae correspond to patients with mild COVID-19 who required hospitalisation. In addition to the physical sequelae, the expe) rience of th[(4]e disease and isolation has generated higher levels of depression and anxiety, as well as memory[(4)(14)] loss in patients who have already been discharged. All these sequelae affect the quality of life, increasing the risk of comorbidities and slowing down the recovery of these patients[(15,16)]. This can be very detrimental, especially in the elderly population[(17,18)]. With all this, it is recommended to continue with early rehabilitation in the post-acute phase after the hospital phase that increases the levels of physical activity, especially in patients without severe disability, which can also be continued with long- term telerehabilitation[(19)].

Telerehabilitation, therefore, seems to be a viable option, *"through wearable devices, mobile phone applications, virtual reality"* etc. [(18)]to reduce the sequelae of the disease and prevent disability[(17,20)]. Telerehabilitation refers to rehabilitation treatments using new information and communication technologies (ICT)[(21)] and is included in the term "telehealth". It can be carried out either live or asynchronously[(22)]. It has already proven to be effective in other pathologies such as cardiac, neurological, respiratory or musculoskeletal[(23-27)]. Among the advantages of this type of intervention is the possibility of providing greater health coverage to people with geographical[(28)] barriers, with other comorbidities, in a situation of dependency or who must be confined to their homes[(29)]. In addition, several studies confirm that working with remote technologies has a positive influence on patient adherence. A [(30,]large[31)] part of the population who are prescribed home rehabilitation do not do it as often as recommended.[(32)]

For this reason, it is necessary to increase the level of adherence of home rehabilitation patients, and telerehabilitation could be the answer, improving patient[(29)] satisfaction and thus patient adherence.

The differentiating and innovative element of this study lies, on the one hand, in the method of telerehabilitation: asynchronous; on the other hand, in the patients: focusing on treating the sequelae in discharged patients; and finally, in the type of intervention programme based on physical rehabilitation: focusing on improving fatigue. There are no previous studies that have assessed the efficacy of an asynchronous programme, but most have focused on the analysis of synchronous[(32)] telerehabilitation, so studies are needed to determine whether asynchronous telerehabilitation is also effective in these patients.

Currently, discharged patients continue their rehabilitation with health recommendations and in some cases regular check-ups. This project would make it possible to offer a free service for the patient that is easy to implement and follow up and which, a priori, would be of benefit to patients and a viable alternative in terms of cost/benefit in the long term[(29)].

According to the results revealed after this feasibility study, the aim is to adapt the intervention for a future clinical trial to provide data that can be extrapolated to the population and thus be able to extend this type of remote intervention to other musculoskeletal pathologies within rehabilitation services.

2.- HYPOTHESIS

The post-COVID-19 sequelae, both from hospitalisation and/or confinement, as well as from the disease itself, are generating a decrease in the physical capacity of patients and an increase in fatigue as a consequence[(33)]. These patients are no longer receiving inpatient rehabilitation treatment, which is why telerehabilitation appears as a short- and long-term complement that could help patients to achieve a functional state and, therefore, a better quality of life.

2.1.- MAIN HYPOTHESIS

H1: the implementation of a programme of weeks12 of physical therapy and therapeutic education using asynchronous telerehabilitation software is feasible and preliminarily effective in increasing levels of physical fitness in terms of fatigue, functional capacity, self-efficacy in managing the process and in improving psychosocial factors (quality of life, depression-anxiety-stress, motivation, commitment and perceived social support), as well as in the adherence to treatment, of patients who have been admitted for COVID-19 in the Hospital Provincial Nuestra Señora de Gracia (HPNSG) and the Hospital Royo Villanova (HRV) of Area 1 of Zaragoza Health, and who have been discharged.

OBJECTIVES

3.1.- GENERAL OBJECTIVE

To analyse the feasibility and preliminary efficacy on physical condition of a 12-week physical therapy and therapeutic education programme using asynchronous telerehabilitation in post-COVID-19 patients who are being discharged from the HPNSG and HRV, and to compare its effects with patients who have completed the same programme, but in a non-telematic format.

To determine the main physical sequelae, as well as the influence of psychosocial factors derived from COVID-19 in patients who have been discharged (maximum 3 months before the start date of the study) in Area 1 of Zaragoza Health and who had been admitted to the HPNSG and the HRV, by means of a survey of needs designed for this purpose.

3.2.- SPECIFIC OBJECTIVES

To analyse the effects on fatigue severity of a physical therapy programme using asynchronous telerehabilitation software in post-COVID-19 patients being discharged from the HPNSG and HRV, as measured by the fatigue severity scale (FSS).

To assess the change in functional capacity (aerobic endurance and strength assessed in activities of daily living) of a physical therapy programme using asynchronous telerehabilitation software in post-COVID-19 patients being discharged from the HPNSG and HRV. Measuring the change in aerobic endurance with the minute 6walk test, upper and lower limb strength with two items of the senior fitness test (FST) and the post-Covid-19 functional status scale (PCFS).

3.- To analyse changes in psychosocial factors: self-efficacy in the management of the process, depression, anxiety and stress, motivation, engagement and perceived social support after the implementation of a physical therapy programme by means of an asynchronous telerehabilitation in post-COVID-19 patients being discharged from HPNSG and HRV, measured by the General Self-Efficacy Scale (GSES-12), Depression, Anxiety and Stress Scale (DASS-21), behavioural in sport (BRSQ-36), the patient activity record, and the multidimensional of perceived social support (MSPSS) respectively

4.- To analyse the changes in quality of live of a physical therapy programme using asynchronous telerehabilitation software in post-COVID-19 patients being discharged from HPNSG and HRV with the SF-12 quality of live scale (version 2).

5.- To determine the level of adherence to a physical therapy programme, recruitment and satisfaction rates, in post-COVID-19 patients being discharged from HPNSG and HRV by recording diaries and a brief interview on the satisfaction and acceptability of the procedures on a 5-point Likert scale in both groups.

4.- METHODOLOGY

4.1.- DESIGN

Pilot and feasibility study with a single-blind randomised clinical trial design. The study has been approved by the management of the HPNSG of Zaragoza and the management of the HRV of Zaragoza, and has the authorisation of the Vice-Rectorate of Research of the University San Jorge. In addition, it will be registered at clinicaltrials.gov.

The feasibility study will have two simultaneous but distinct phases:

- One of the phases will consist of conducting an initial survey to detect needs derived from COVID-19. The survey will be carried out on all patients who have been discharged from the HPNSG and HRV no more than months3 before the start date of this study and who have given their informed consent at the time of hospital discharge to participate in the observational study.

- The other phase will consist of recruiting patients who are discharged from the HPNSG and the HRV from the start date of the present study and who meet the eligibility criteria.

  These patients (who are discharged) will also be given the needs survey to get an overview at the time of their enrolment in the study, should they wish to participate and sign the consent form.

  Recruitment for the clinical trial will take place as patients are informed that they will be discharged from the start date of the study, being informed by means of the patient information sheet, and signing the informed consent form if they wish to participate (in person before hospital discharge).

In addition, a telephone follow-up will be carried out every two weeks from the start date of the intervention programme throughout the duration of the study. A post- intervention follow-up at 3 and 6 months is also planned.

4.2.- STUDY SUBJECTS

All patients who have been discharged from the HPNSG and HRV no more than months3 before the start date of this study will be considered as subjects of the observational study. These patients will simply be surveyed using a needs survey created specifically for this study.

Longitudinal trial subjects will be considered to be patients of the HPNSG and HRV of Zaragoza, admitted for COVID-19 and to be discharged at the time of inclusion in the study.

Previous medical history data will be provided by the HPNSG and HRV. Recruitment for the longitudinal trial will be performed at the HPNSG and the HRV, and the

The needs survey for the observational study will be carried out on site at the HPNSG, and will be conducted by the HPNSG healthcare staff of the Hospital's physiotherapy service, with the authorisation of the Hospital Management, who will inform potential participants in person by means of the information sheet, and will ask patients who meet the inclusion criteria and who decide to participate to give their consent and sign it. Subsequently, they will be given an appointment to undergo the pre-intervention assessment (detailed below).

4.2.1.- Inclusion Criteria

- Age between 18 and 75 years*.
- Patients who have been hospitalised for more than 5 days for COVID-19.
- Patients who have been discharged from HPNSG and HRV and have a fatigue score higher than 4 points on average on the FSS**.
- Independent ambulation, even with the use of any technical aids.
- Have signed the informed consent form.

*The upper age limit of 75 years is intended to avoid bias in the intervention as it is a method using technological applications that require access to and knowledge of basic internet use.
On the other hand, the average patient in Spain hospitalised for COVID-19 has an age of years 69with some risk factor (hypertension 51%; dyslipidaemia 39.7%; diabetes 19.4%).

**A score of less than 4 points on the FSS is considered low fatigue. The programme focuses on intervening on those patients with moderate or high fatigue on the understanding that they have the greatest rehabilitation needs.

**The physical conditions of patients who have been both in ICU/floor and on the ward alone will be assessed in order to stratify according to their situation at the time of discharge in relation to the severity of fatigue.

4.2.2.- Exclusion Criteria

- Patients with severe central and/or peripheral neurological diseases that prevent them from following the programme*.
- Patients with severe respiratory insufficiency: SaO_2 90% or respiratory rate ≧30[34]*.
- Patients with rheumatic pathology or acute musculoskeletal injury in their medical history*.
- Patients with unstable cardiac comorbidities, such as arrhythmias, high blood pressure, angina pectoris or other pathologies that contraindicate moderate intensity training*.
- Patients without access to mobile internet or computer with internet at home**.
- Patients scoring ≤24 on the Mini-Cognitive Examination (MEC)[35] (ANNEX I)**.
- Who are not able to follow oral and written instructions in the Spanish language**.

*Patients whose medical condition prevents them from performing this physical activity programme without live monitoring in a clinically safe manner are excluded because the tool used does not have *in vivo* monitoring during exercise.
** On the other hand, the aim is to exclude those people who cannot follow the treatment, either due to lack of cognitive or language comprehension, or due to accessibility, avoiding biases in the intervention.

4.2.3.- Randomisation

Patients screened for the longitudinal trial who wish to participate, meet the inclusion criteria and sign the informed consent form will be enrolled in the study.

The feasibility clinical trial will feature two groups, an experimental group that will receive the telerehabilitation programme in telerehabilitation format and a control group that will receive it in paper format.

Randomisation will be carried out using the software www.randomizer.org, giving each subject an identification code (IC) to guarantee anonymity. The person responsible for the project will know the list of cases with the IC and will remain blinded to the patient. The evaluator will remain blinded to the identification number and the assignment. Once the study is completed, the sealed envelope with the list of cases and IC will be opened. Three initial levels of fatigue will be stratified according to the fatigue severity scale (FSS) and homogeneous groups will be created, controlling for possible confounding variables such as taking medication or having received previous or current physiotherapy treatment.

4.3.- INTERVENTION

4.3.1.- Intervention protocol.

A physical rehabilitation programme will be designed with three levels of intensity, each consisting of six exercises, following the recommendations of the Physiotherapy Associations of the Autonomous Communities of Andalusia, Madrid and Aragon and the recommendations of the physiotherapy area of the Spanish Society of Pneumology and Thoracic Surgery SEPAR(36,37). The first level will include those who obtain a score greater than or equal to 4 and less than 5 (4 to 4.9); the second level will consist of those who obtain a score greater than or equal to 5 points on average and less than 5 points on average (4 to 4.9); the second level will consist of those who obtain a score greater than or equal to 5 points on average and less than 5 points on average (4 to 4.9).
6 (5 a 5,9). The third level will consist of the most fatigued patients, with mean FSS scores between 6 and 7 (6 to 7).

The exercise programme will be divided into an aerobic exercise part (1 exercise), a strength training part (3 exercises) and a breathing exercise part (2 exercises) for 12 weeks, three days a week (ANNEX II).

Similarly, following the needs survey, a list of recommendations for self-management of the sequelae of the disease will be created. For both the exercise programme and the therapeutic education programme, explanatory videos will be created for the intervention group and a leaflet for the control group. All patients will be provided with the same physical rehabilitation and educational programme, with each allocation group differing in the format of the prescription.

The design of this programme will be carried out by the researchers: B. Carpallo, C. Jiménez, S. Calvo, S. Pérez and P. Herrero.

Patients who have been enrolled after meeting the inclusion criteria and who have signed the informed consent form (in person) will be scheduled for a pre-intervention assessment using the test battery discussed in the following section.

At the end of this assessment, a member of the research team will explain to each patient the battery of exercises prescribed according to their initial level of fatigue, depending on the group to which they have been assigned (they will have a list with the order of citation and the CI, without knowing the patient's name). At the end, understanding of the intervention will be checked by asking them to explain the exercises. The control group will be given the booklet with the exercises at this point and the experimental group will have the Health for All (HEFORA) application installed and explained to them, a platform designed to prescribe therapeutic exercise by trained professionals and which is currently available free of charge from the Official College of Physiotherapists of Aragon for all its registered physiotherapists. The platform registers in an encrypted and anonymised way the personal data (name and surname) accesses of each user to the platform and the number of times they have viewed the videos. These access and viewing data will be provided only to the principal investigators of the pilot study in order to record adherence and commitment to the telerehabilitation programme. The patient on their first access and registration on the platform must accept the platform's data protection and privacy policy (APPENDIX XVIII). In addition, the complete data protection policy of the platform is provided in the Patient Information Sheet.

After 12 weeks, each patient will be called back to the hospital after the last intervention session for the post-intervention assessment with the same scales as the initial assessment. Satisfaction with the assigned programme will also be assessed with the Likert scale.
The person responsible for the pre/post-intervention assessments is: L. Romo, physiotherapist at HPNSG. The person responsible for the explanation of exercises, handing out of leaflets and installation of the application and explanation of its use will be: B. Carpallo.

During the weeks12 of intervention, all patients will be monitored every two weeks. They will be called by telephone and their fatigue during the sessions will be assessed using the

Borg effort scale. If it is below 5/10 they will be modified.

The presence of obstacles in the follow-up will also be [36,37]. assessed.

The members responsible for the bi-weekly monitoring are the researchers: S. Calvo; C. Jiménez and B. Carpallo.

At 3 and 6 months after the end of the intervention, all patients will be given the initial needs survey by telephone to assess the general state of the patients in relation to their activities of daily living and fatigue. For the follow-up of psychosocial factors, the general self-efficacy survey abbreviated (GSES), the stress and anxiety scale (DASS- 21) and the quality of life scale (SF-12 version 2) will be administered.

During the whole process, there will be a methodological control by S. Calvo and C. Jiménez, and specifically of the psychosocial variables by the team psychologists I. Liñares and L. Bafaluy.

Experimental group

The experimental group will receive a therapeutic and educational exercise programme in the form of text and videos via the HEFORA application, so that they can replay the videos as often as they need to.

The HEFORA platform will not have any economic cost for any of the participants in the study. As mentioned above, the platform will record, in an encrypted and anonymised manner, each user's access to the platform and the number of times they have viewed the videos.

After the pre-intervention face-to-face assessment, these patients will meet with the researcher responsible for explaining the programme. The three levels of physical activity will be explained to this group and the application will be installed on their mobile phone. Its use will also be explained to them.

Each patient will have free access to the platform, with the complete programme of exercises and recommendations, from their private account.

Depending on your initial level of fatigue, you will be prescribed the corresponding videos to perform to facilitate their viewing and execution. Each video will appear in the "library" of the application. The videos will be designed and recorded specifically for this purpose.

Each video shows a trained person performing the exercise step by step. In addition, at the top of the video you will find the fundamental indications to be able to perform the exercise correctly.

Every two weeks, patients will receive a monitoring call to assess their level of fatigue, and if this has improved, the videos at that level will be closed and those at the next level will be activated. The platform also allows the sending of control messages to further individualise the home treatment.

In addition to the exercises, each patient will have at their disposal videos with health education recommendations that they will have to watch throughout the intervention. Each viewing is recorded and can only be checked by the research group at the end of the intervention. Patients will know at all times that each access to the platform is recorded. The participants of the experimental group will also have a section for annotations, where the participants will have to register weekly on this platform if they have carried out the exercise programme and if they have carried out additional physical activity.

Every two weeks, the aforementioned control calls will be made, in which they will be asked about the course of the treatment, if they present any difficulties in the implementation of the programme and their level of fatigue after the programme has been carried out. As their fatigue decreases during the exercises, the pattern of exercises will be modified and they will move on to the next level.

4.3.3.- Control group

The control group will receive, after the initial assessment with the HPNSG physiotherapist, a paper booklet with clear pictures and descriptions of the three levels of exercise intensity. In addition, there will be recommendations for therapeutic education. The researcher in charge of handing out the material will make sure that the exercises are understood and will mark the initial starting level of the activity, according to their initial level of fatigue.

At the same time, the procedure for control calls will be explained to them every

two weeks, which will be the same as for the experimental group. As their fatigue decreases during the exercises, the exercise pattern will be modified and they will move on to the next level.

They will also be given a diary in which they should record each week whether they have performed the proposed exercises (in load and repetitions and if they have done any additional physical activity in addition to their daily life activities such as walking or doing sport for more than a few minutes 30that day).

4.4.- VARIABLES

4.4.1.- Main variable: observational pre-phase: detection of needs.

***Post-COVID-19 needs assessment survey*:** the aim of which is to identify the main physical needs as well as psychosocial factors arising from COVID-19.
In order to detect the needs of patients who have passed the COVID-19, a survey has been designed that can be carried out in person or by telephone (with prior informed consent) based on other scales that allow the physical rehabilitation programme and the educational recommendations of the subsequent phase of the clinical trial to be adjusted (ANNEX III).

It is made up of four sections: personal data, living habits, characteristics of hospital admission and consequences of COVID-19. This last section, specific to the detection of needs, is made up of four parts:

Psychosocial factors based on suffering the COVID-19 with dichotomous questions on the presence of anxiety, depression, asthenia, stress, memory loss or headaches.

Functional status: based on the post-COVID-19 functional status scale (PCFS). This scale, specifically designed to detect the functional status of patients who have passed COVID-19[38)],[(20,] classifies patients into 4 levels of functional status, from 0=unlimited to 4=severely limited.

Level of fatigue and its interaction with daily life: based on the fatigue severity scale (FSS).[39,40] (discussed in depth in section 4.4.2).

Other symptoms: loss of strength, pain and other symptoms that affect functioning in activities of daily living (ADLs).

4.4.2.-Main clinical trial variable: fatigue level

Fatigue can be understood from various aspects, not only physical. The aim of this study is to intervene in patients' functional capacity, for which it is essential to act on the initial level of fatigue. There are currently no validated scales for post-COVID-19 patients.

*This variable will be measured during the pre- and post-intervention assessment. It will serve as a reference for grouping participants into fatigue levels and prescribing the programme that matches their physical needs in terms of intensity.

- Fatigue Severity Scale (FSS)[39], validated in Spanish by Bernal-Vargas et al. and Duarte et al.[40,41] It was initially designed by 1989Krupp et al. for neurological diseases such as lupus erythematosus and multiple sclerosis. It has been progressively validated in different languages, including Spanish, and in other population groups, such as Parkinson's disease, cancer, chronic pain, hepatitis, ankylosing spondylitis, in the elderly population, in a specific professional sector, etc...[42-45] The FSS fits our purpose by relating the level of fatigue to functional capacity, and it is also used to measure fatigue derived from pathology or ageing as in this case (with an expected elderly population). It also has the advantage of being brief, which facilitates its application and can be carried out by telephone or in person.

The FSS is a self-reported scale composed of 9 items relating the severity of fatigue to daily activities. Each item is rated 1from 71=strongly disagree to 7=strongly agree (ANNEX IV). A mean of less than 3 points is considered to be no fatigue[44], less than 4 is considered to be mild fatigue, more than 4 is considered to be severe fatigue. This range can be divided into three levels of fatigue: 4=fatigue

severe borderline fatigue; 5=moderate fatigue; 6-7=severe high fatigue. The original version only marks the cut-off at 4 points: presence or absence of fatigue.

4.4.3.-Secondary variables

The selection of these tests has been made according to the current literature on rehabilitation treatments in COVID-19 patients, seeking the greatest homogeneity of tests and recommendations[(16)].

*4.4.3.**1-Functional** capacity:* measured with the 6-minute walk test, the 30-second sit- to-stand test, the 30-second elbow flexion test, and the post-COVID-19 functional status scale.

- **Minute6 walking test (6MWT**). Validated in Spanish by Gimeno-Santos et al.[(46)] Participants in the study should walk the maximum possible distance for 6 minutes. During the walk, patients can rest if needed. They will be monitored with a pulse oximeter and will follow the instructions of the physiotherapist. The distance will be measured in metres (ANNEX V).

- **30-second sit-to-stand test (STST): it** is part of the Senior Fitness Test (SFT) designed and validated by Rikli and Jones[(47)] and in Spanish by Cuellar et al. and Cobo- Mejia et al[.(48,.49)] This item is currently used as an isolated test together with the elbow flexion test [(50),]especially to assess the weakness of elderly, respiratory and COVID- 19[(16,51-53)] patients.[38,] Another criterion for the choice of this second30 test was the expected age of the participants and their low physical condition and its validation with respect to the 1-minute test in elderly patients.[(54)]

This test should be performed with a chair of sufficient height for the feet to rest on the floor. The patient's arms should be crossed at the chest. Once in this position, they should rise and return to the initial position as many times as possible within 30 seconds . . (ANNEX VI).

- **Elbow flexion test (ACT)[(47,53)]: this** is also part of the SFT, mentioned in the previous test, although it is also used as a stand-alone test to assess strength at the elbow.

in deconditioned patients or the elderly population. From a seated position, as many elbow flexion-extension movements as possible should be performed in 30 seconds, with the dominant arm, with a 2kg weight (ANNEX VII).

- **Post-COVID functional status scale** (PCFS)[55]: scale specially designed to assess the functional capacity of patients who have undergone COVID-19 at the time of discharge, at 4 weeks, at 8 weeks and at 6 months. It is currently not a validated scale due to the recent emergence of the disease, but it is in the process of translation and validation[16]. Due to the scarcity of our own tools and given that we complete the assessment with other validated tools, we believe that it is appropriate to attach this assessment scale translated into Spanish, in order to obtain additional information. This scale can be administered by telephone or self-administered. It is composed of post-COVID-19 levels4 of disability. F=death; 0=no limitation; 1=no significant functional limitation; 2=mild functional limitation; 3=moderate functional limitation; 4=severe functional limitation (ANNEX VIII).

Psychosocial *factors:* Self-efficacy in the management of the process, depression and anxiety, measured with the GSES and DASS-21 general self-efficacy scales respectively.

- **General self-efficacy scale (GSES-12):** is a validated and abbreviated scale derived from the original scale composed of 30 items and two subscales[56]. The abbreviated version was developed by Bosscher et al. for elderly people[57], which is why it fits the profile of the patients estimated for this study. Validated in Spanish by López- Torrecillas, Herrero et al., and Sanjuán et al. and consisting [58-60]of 10 items, it is assessed on a Likert scale from 1=never happens to me to 5=always happens to me. The items are divided into 3 blocks: initiative, effort and perseverance (ANNEX IX).

- **Depression and Anxiety Scale (DASS-21)[61]**: is an abbreviated version of the DASS-42 scale designed in 1995 by Lovibond et al[62]. Validated in Spanish by Daza et al. and Ruiz et al.[63,64] It is composed of three content blocks (depression, anxiety and stress) assessed using a Likert scale from 0=never happened to me to 3= often or almost always happened to me (ANNEX X).

- ***Behavioural regulation in sport questionnaire (BRSQ-36):*** measures extrinsic motivation through different factors depending on whether the patient performs the

believe that it is part of a healthy lifestyle (integrated regulation), by believing that

the activity is important but not pleasurable (identified regulation), out of feelings guilt (introjected regulation) and/or external pressures (external regulation). The intrinsic motivation is measured by three factors: knowledge (the pleasure to learn more about physical activity), performance (the enjoyment of achieving goals) and execution and stimulation. Other factor it measures is demotivation, an action characterized by a lack of interest towards practice and feelings of frustration. We found that this scale is validated by the Spanish context[65]. Its items are rated on a Likert scale ranging from the 1(not true at all) to 7 (very true) (ANNEX XI).

- Commitment[66, 67]: will be measured by participation. This measurement will therefore be achieved through adherence to the programme which is another variable of this project which shall be established by means of a daily activity log to be completed by the patient. (This will correspond to the annex described for the appendix of adhesion)

- ***Multidimensional Scale of Perceived Social Support (MSPSS)[68]:*** validated in Spanish population by Landeta and Calvete, obtaining high psychometric properties. It is considered a priority scale to be applied to people who are in recovery process. It measires social support in three dimesions (family, friends and others). The version used consist of 12 items that are answered on a Likert scale ranging from (1, totally desagree) to (7, strongly agree) (ANNEX XII).

4.4.3.3.-Quality ***of life*** measured with the SF-12 (version 2)

Self-reported quality of life questionnaire (SF-12), version[70] **.2** Patients are asked to fill in the self-administered questionnaire consisting of 12 questions on self-perceived health. Version 2 is an abridged version of the SF-12, which2002, has 36been validated in Spanish by Vilagut et al. and Guerra-.

Tapia et al.[71,72] It consists of 12 questions covering 8 dimensions (physical function, social function, physical role, emotional role, mental health, vitality, bodily pain and general health) with several response options. The final score ranges from 1 to0 1 for 100,each of the dimensions, with 100 being the worst possible score (ANNEX XIII).

4.4.3.4.-Adherence measured with diaries and entries in the telerehabilitation platform.

- Adherence: diaries and platform "annotations" register: to be coded: 1=if they have done the activity, 0=if that day they have not done as prescribed, 2=if that day they have done more additional activity. Additional physical activity will be understood as voluntary physical activity that exceeds the usual daily activity such as: light walking for more than half an hour; sport for more than half an hour or other additional voluntary exercise (ANNEX XIV).

*4.4.3.5.-**Study** feasibility*

- Feasibility of the study: this will be calculated by the recruitment rate, in addition to the adherence rate indicated above, the report of satisfaction with the exercise programme and the application assessed at the end of the study, and incidents assessed during the follow-up calls. This will be recorded on a Likert scale 0=very dissatisfied to 4=very satisfied. Incidents will be recorded as: 0=no problem; 1=some problem solved; 2=problem making it impossible to follow up that period (ANNEX XV).

*4.4.3.6.-Level of **fatigue perceived during the intervention***

*Variable measured during the two-weekly controls to modify the intensity level of the programme.

- Borg[31] **effort scale**: a subjective scale of effort that quantifies from 0=very easy to 10=very hard the level of effort perceived when performing an activity.

This tool will be used to assess the level of effort of each patient in order to adjust the level of the proposed exercises. It will also be used for each patient to control the recommended intensity during the treatment.

The following table shows the measurement schedule for each of the variables within the general periodisation scheme of the project:

	T1. Fase Previa	T2. Fase 1. Observacional	T3. Fase 2 Pre-Intervención	T4. Fase 3 Intervención Día 1	T5. Día 15	T6. Día 29	T7. Día 43	T8. Día 57	T9. Día 71	T10. Día 85	T 11. Fase 4. Post-Intervención	T 12. Fase 5. Seguimiento 1	T 12. Fase 5. Seguimiento 2	T 13. Fase 6.
**Diseño estudio**	X													
**Diseño del programa de ejericcios y educativo**	X													
**Estudio observacional**		X												
Encuesta de necesidades		X												
**Reclutamiento/Selección**		X												
**Aleatorización**			X											
**Evaluación pre/post-intervención**				X							X	X	X	
Severidad Fatiga-FSS				X							X	X	X	
Estado Funcional Post-Covid-PCFS				X							X	X	X	
Capacidad aeróbica submáxima-6MWT				X							X			
Capacidad física mmii-STST				X							X			
Capacidad física mmss-ACT				X							X			
Autoeficacia-GSES-12				X							X	X	X	
Depresión-ansiedad-estrés-DASS-21				X							X	X	X	
Apoyo social-MSPSS				X							X	X	X	
Calidad de vida-SF-12				X							X	X	X	
Motivación-BRSQ-36				X							X	X	X	
**Intervención**					X	X	X	X	X	X				
Adherencia/compromiso-Dirarios de actividad					X	X	X	X	X	X				
Fatiga-BORG					X	X	X	X	X	X				
Incidencias Likert					X	X	X	X	X	X				
Satisfacción Likert										X				
**Seguimiento**												X	X	
Análisis de datos														X
Difusión de resultados														X

4.5.- SAMPLE SIZE

The sample size will be 32 patients, 16 in each group. Calculated using G*Power 3.1 software (Heinrich-Heine University of Düsseldorf, Germany), based on an effect size f of 0.25, a level of 0.05, a β level of 20% and a power of 80%, plus an estimated dropout rate of 30%.

4.6.- STATISTICAL ANALYSIS

Statistical analysis will be performed with IBM-SPSS Statistics version 25 software. The Kolmogorov-Smirnov test will be used to determine the normality of the data. Intra- group variables will be measured with the Student's t-test for related samples and U- Mann Whitney for non-parametric variables. Between-group and within-group analyses will be performed using a mixed model analysis of variance (ANOVA) for repeated measures with Bonferroni post hoc pairwise comparisons when a normal distribution is detected. Non-parametric analysis will be performed when a non-normal distribution is assumed, using the Mann-Whitney U-test for between-group comparisons and Friedman's test with Tukey's test to highlight within-group differences.

Dichotomous variables will be analysed using the chi-square test. A significance level of 95% ($p \leq 0.05$) will be assumed. Descriptive statistics will also be used to report on the feasibility of recruitment and adherence to the programme. Variables will be described as mean and standard deviation (SD) or median and interquartile range. If there are more than 15% dropouts, an intention-to-treat (ITT) analysis will be performed. Effect size will be calculated using Cohen's d to determine clinical significance: insignificant, small, medium and large differences will be reflected in effect sizes of <0.2, 0.2–0.5, 0.5–0.8 and >0.8, respectively.

4.7.- BIASES AND LIMITATIONS

The present feasibility study has the risk of memory bias in participants who do not fill in the diaries every day. In order to prevent them from taking notes at the end of the study and not being correct, a control every two weeks has been planned in which they will be personally reminded if they have recorded the activity they have carried out.

To avoid bias in the assessment of the intervention, the physiotherapist in charge will not know the assignment of each subject.

In order to reduce bias in the analysis of the results, those cases that drop out of the study due to problems with the platform, lack of motivation when carrying out the prescribed programme or any other incident will not be replaced, as long as they carry out the post-intervention assessment, as the aim is to find out the limitations to adherence with the aim of proposing improvements in a randomised clinical trial with a larger sample size.

Follow-up will be conducted in both groups, this may affect the increase in adherence in both groups.

5.- ETHICAL ASPECTS

5.1.- INFORMED CONSENT

All patients will sign an informed consent form on the first day they are seen. Participation is free and voluntary. No participant will receive financial or any other kind of compensation for their participation.

5.2.- RISKS AND CONTINGENCIES

The planned intervention does not present any health risks to any of the participants, as the exclusion criteria have been adjusted to avoid the participation of patients in whom an intensive intervention might be contraindicated. All patients will have the telephone number of the investigator at the reference hospital for any incident and of the principal investigator.

In addition, the experimental group will have a communication channel through the platform. As registered physiotherapists, all researchers are covered by civil liability insurance.

5.3.- DATA PROTECTION

The Organic Law on Data Protection will be complied with at all times. Coded data will be used so that no subject data will be included in the study database and no one other than the research team will have access to the participant's identity.

The entire privacy policy of the HEFORA platform is available at https://es.hefora.com/privacy-and-cookie-policiesyhttps://hefora.com/wp-content/uploads/2018/08/Politica_de_de_PRIVACIDAD.pdf. FISIO CONSULTORES S.L. is responsible for the processing of personal data of users of the platform being the data processed in accordance with Regulation (EU) 2016/679 of the European Parliament and of the Council of 27 April 2016 on Data Protection (GDPR). As a user you have the right to access, rectify and delete the data, as well as other rights indicated in the additional information via email address info@hefora.com and dpo@fisioconsultores.com. (ANNEX XVIII).

The HEFORA platform first collects some initial data to create a user account, which are a username, password, email address and name and surname. Once the patient has created their account, they contact the researcher and digitally accept the authorisation document for data processing.

Once this link has been established, the researcher can include in the platform the data collected directly by the researcher or access the results of the questionnaires self-administered by the patient (data described in the research project). The date and time of signature is recorded and can be consulted by both the patient and the researcher.

The data that may be collected on the platform after this acceptance, if the user decides to fill them in, will be, depending on the user (researcher/professional or patient):

- Professionals: DNI/NIF, Name and surname, postal or e-mail address, telephone, image, manual signature, personal characteristics, social, academic and professional circumstances, employment details, commercial, economic, financial and insurance information, transactions of goods and services.

- Users: DNI/NIF, Name and surname, postal or e-mail address, telephone, image, manual signature, personal characteristics (personality or behaviour), personal characteristics, social, academic and professional circumstances, employment details, commercial, economic, financial and insurance information, transactions of goods and services.

- Patients: DNI/NIF, Name and surname, postal or e-mail address, telephone, image, manual signature, physical marks, health, personal characteristics (personality or behaviour), personal characteristics, social circumstances, academic and professional, employment details, commercial, economic, financial and insurance information, transactions of goods and services.

2.- Regarding the data kept, for the duration of the investigation, the data kept will be those indicated above. If the service is terminated, the data will be deleted except for those that must be kept due to legal obligations. And for further use, research, these data are anonymised and therefore are not personal. The user has the possibility to delete his account also after the end of the study.

3.- The Privacy Policy is the one that appears on the hefora.net website and only those who do not have access to it will be able to access it.

researchers have access to the data, with the exception of name and surname and email, which can be accessed by the company that owns Hefora, which does not pass this data on to third parties.

4. Regarding security measures, the platform is GDPR compliant, and has the following security measures in place:

- At the server level, the server that manages hefora.net is hosted in a private data centre with cloud technology on OpenStack. The provider has no access to the server system or its data. The snapshots taken are stored in another data centre on the same premises and are transferred via an isolated internal network. The administration of the server is done under the OpenSSH service with authentication by password or RSA private key. The communication between the user and the web service is carried out under SSL with an EV certificate (SectigoRSA Extended Validation Secure Server CA). The communication between the web services and the database is not exposed to the outside and is reduced to its isolated internal network managed by the Debian 9.9 system services. Data backups are encrypted at the system level underAES-256 and transferred with OpenSSH over the internal network under VLAN to another backup server. Like the hefora.net server, this backup server runs with cloud technology on OpenStack and the provider also has no access to the system. The administration of this server is also carried out under the OpenSSH service with password or RSA private key authentication.

- At the database level, all personal and medical data are stored with record-level encryption, which means that everything is incomprehensible without first being decrypted. In addition, medical records are properly pseudo-anonymised so that it is not possible to directly link the natural person and his or her medical data. In addition, all documents exchanged with users (e.g. videos) are encrypted through an external security provider, chino.io, which is ISO certified.27001.

- Regarding the export of questionnaires: for each questionnaire and the person who has filled it in, what is done is to generate an encrypted identifier from the original identifier of that person, so that even if you have access to the

If the original database is exported, it would not be possible to know which original identifier it refers to, and therefore it would not be possible to know which person filled in which questionnaire. In this way, with the exported list of questionnaires, it is possible to know if 2 different questionnaires belong to the same person (they will have the same encrypted identifier), but it is not possible to know to which natural person they correspond. In other words, they are anonymous.

- As for the specific process of pseudo-anonymisation, what has been done is to break the link in databases between the patient and medical data tables. There is no identifier to indicate which patient each medical record corresponds to; they are independent tables. In another separate table, unrelated to the previous ones, what is being done is to store the patient's identifier encrypted with an AES algorithm and256 their personal data also encrypted with the same algorithm. In order to know to which individual a medical record corresponds, this identifier would have to be decrypted, so there is no way, if you have access to the database, to establish the relationship between patient and medical record without knowing the decryption key and algorithm. Moreover, no personally identifiable data is stored in the medical history table. The result is that the medical records are pseudo-anonymised, as their information does not contain any personal data and no direct relationship with the patient can be established. The same applies to the rest of the data related to the clinical history: questionnaires, prescriptions, etc.

In accordance with Regulation (EU) 2016/679 of the European Parliament and of the Council of 27 April 2016 on Data Protection (RGPD) and Organic Law 3/2018, of 5 December, on Personal Data Protection and guarantee of digital rights, the participant is informed that FUNDACION UNIVERSIDAD SAN JORGE will be responsible for the processing of his/her personal data.

As a participant in the study you may exercise your rights of access, modification, opposition, cancellation, limitation of processing and portability by contacting the Data Protection Delegate of the University, attaching a photocopy of your ID card or equivalent to your request to the registered office of USJ at Autovía A-23 Zaragoza- Huesca, km. 299, 50830- Villanueva de Gállego (Zaragoza), or to the e-mail address privacidad@usj.es. Likewise, you have the right to contact the Spanish Data Protection Agency in the event that you do not see your request correctly dealt with.

the exercise of their rights.

The participant may withdraw from the study at any time by informing the principal investigator but is informed that his/her data cannot be deleted in order to ensure the validity of the research and to guarantee compliance with the legal duties of the person in charge.

5.4.- CARE IMPLICATIONS

The HPNSG hospital centre will only serve as a reference centre from which patients will be recruited and where pre- and post-intervention assessments will be performed. The rehabilitation programme will be carried out at home or in the context of the patient and therefore does not interfere with the clinical development of the centre. For the assessment days, the assessing physiotherapist of the centre will be allowed to dedicate himself/herself for this purpose, organising the care agenda in advance in accordance with this requirement.

6.-CRONOGRAM

TIMETABLE	MONTHS										
PHASES	JANUARY	FEBRUARY	MARCH	2º half MARCH	APRIL	2º half APRIL	MAY	2º HALF MAY	JUNE	SEPTEMBER	JANUARY
Study design											
Creation of the exercise protocol											
Creation of educational videos											
Preliminary phase, observational study											
Data analysis, previous phase observational											

Selection and randomisation clinical trial											

Pre-intervention assessment											
Intervention											
Control every two years telephone weeks											
Post- intervention evaluation											
Initial data analysis											
Follow-up at 3 and 6 months											
Analysis of monitoring data											

7.-ANNEXES

ANNEX I. Mini Cognitive Examination (MEC)

	PUNTUACIÓN
**ORIENTACIÓN TEMPORAL:**<br>¿En qué día estamos?..................... ¿En qué fecha?............................... ¿En qué mes?.................. ¿En qué estación?............................... ¿En qué año?................	_____(5)
**ORIENTACIÓN ESPACIAL:**<br>¿En qué hospital o lugar estamos?... ¿En qué piso o planta?................ ¿En qué pueblo o ciudad?........................... ¿En qué provincia, región o autonomía?................ ¿En qué país?.................................	_____(5)
**FIJACIÓN:**<br>Repita estas 3 palabras: 'peseta- caballo- manzana'..<br>(*Repetir hasta 5 veces, pero puntuar solo el primer intento)<br>(**Pueden utilizarse series alternativas de palabras cuando se trata de reevaluaciones ej. Libro, queso, bicicleta)	_____(3)
**CONCENTRACIÓN Y CÁLCULO**<br>Si tiene 30 ptas y le van quitando de 3 en 3, ¿cuantas le quedan?.............(27)<br>¿y si le quitan otras 3?.........(24)<br>¿y ahora?...........................(21)<br>¿y 3 menos son?..................(18)<br>¿y si le quitan otras 3?..........(15)<br>(Anote un punto cada vez que la diferencia de 3 sea correcta, aunque la anterior fuera incorrecta)	_____(5)
Repita 5-9-2 (hasta que los aprenda). Ahora hacia atrás...<br>(Como alternativa, decirle 'mundo' y que lo repita al revés)	_____(3)
**MEMORIA**<br>¿Recuerda las 3 palabras que le he dicho antes?..	_____(3)
**LENGUAJE Y CONSTRUCCIÓN**<br>Mostrarle un lápiz o un bolígrafo: ¿Qué es esto?<br>Repetirlo con el reloj..	_____(2)
Repita la frase 'En un trigal había 5 perros'..<br>(Repetir hasta 5 veces, pero puntuar solo el primer intento)	_____(1)
Una manzana y una pera son frutas, ¿verdad?<br>¿Qué son un perro y un gato? ;<br>¿Qué son el verde y el rojo?...	_____(2)
Coja este papel con la mano derecha, dóblelo por la mitad y póngalo encima de la mesa	_____(3)
Lea esta frase y haga lo que dice..	_____(1)
Escriba una frase (con sujeto y predicado)...<br>**CIERRE LOS OJOS**	_____(1)
Copie este dibujo... 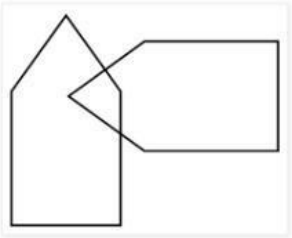	_____(1)
**Puntuación total**	_____(35)

ANNEX II: Initial exercise booklet.

PLAN DE EJERCICIOS

Indicaciones generales:

- Si aparece **dolor**, disminuir la intensidad o parar el ejercicio y ponerse en contacto con la fisioterapeuta encargada del programa.
- Tres días a la semana.
- **La sensación de fatiga** debe ser moderada 6-7 sobre 10 **cansado, pero puede hablar**.

Aeróbico:

Comenzar con 10 minutos al día y **progresar hasta 30** minutos seguidos al día, luego **aumentar la velocidad** para que siempre se sienta cansado.

Ejercicios nivel 1:

Caminar: en casa o en la calle de forma continua.

Subir escaleras o un escalón.

Ejercicios nivel 2:

Subir escaleras o un escalón.

Caminar rápido.

Nadar.

Ejercicios nivel 3:

Trotar

Saltar en estático

Cualquier otro deporte con la intensidad marcada.

Fuerza-Resistencia:

3 series de tantas repeticiones como se pueda. **Descansar 1 minuto** entre cada serie.

No deberá mantener el aire, la respiración debe ser continua y al sacar el aire soplando lentamente.

Ejercicios nivel 1:

- **Subir y bajar un peso con las dos manos:** empezar con una botella de 1 litro llena de agua y progresar a la de 2 litros.
- **Sentarse y levantarse** de una silla ayudándose de los brazos.

Ejercicios nivel 2:

- **Agacharse, coger un peso de 1 kilo y levantarlo por encima de la cabeza.**
- **Sentarse y levantarse** de una silla **sin ayuda** de los brazos.

Ejercicios nivel 3:

- **Agacharse, coger un peso de 2 kilos y levantarlo por encima de la cabeza.**
- **Sentadillas.**

Ejercicios de respiración

1.- Sentado en una posición cómoda durante 5 minutos cuantas veces se quiera. Al menos dos al día.

Inspirar lentamente por la nariz.
Guardar el aire 3 segundos.
Soplar lentamente con los labios juntos.

2.- Tumbado de lateral y luego del otro lado durante 5 minutos, al menos dos al día.

Inspirar despacio por la nariz
Sacar el aire de forma lenta con la boca abierta como empañando un cristal.

ANNEX III: Fatigue Severity Scale (FSS)

Please answer the following statements from 1 to 7. 1 means you strongly disagree and 7 means you strongly agree.

ESCALA DE INTENSIDAD DE LA FATIGA (KRUPP)

	1	2	3	4	5	6	7
1. Mi motivación se reduce cuando estoy fatigado....................							
2. El ejercicio me produce fatiga..							
3. Me fatigo fácilmente..							
4. La fatiga interfiere en mi funcionamiento físico.....................							
5. La fatiga me produce con frecuencia problemas..................							
6. La fatiga me impide hacer ejercicio físico continuado.............							
7. La fatiga interfiere en el desempeño de algunas obligaciones y responsabilidades...							
8. La fatiga es uno de mis 3 síntomas que más me incapacitan....							
9. La fatiga interfiere en mi trabajo, familia o vida social.............							

TOTAL:

ANNEX IV: Needs survey.

Good morning/afternoon, I need you to answer the following questions:

I.- Personal data

- id no: ....
- First name: ....Surname 2nd Surname...........................
- Date of birth: dd..........mes.............year..................
- Vivo
  - Yes
  - No
    - Causes arising from COVID-19:
    - Other causes:
- Genre: ....

II.- Life habits

- Smoking:
  - Ye s
    - Number of cigarettes per day: ....
  - No

- Alcohol intake:
  - Yes
    - Type:
      - Beer or wine
      - High proof alcohol
    - Glasses per week
  - No

- Weekly physical activity:
  - Yes
    - Walking: Days per week:
    - Exercise bike: Days per week
    - Others:
      - Which:
      - Days per week
    - Total hours per week:
  - No

III.- Income characteristics

- Days of hospital admission:
- Form of stay:
  - Plant:
  - UCI:
    - Oxygen:
      - Nose goggles:
      - Intubation:
    - Sedation:
      - Ye s
        - Days:
      - No.

- Hospital rehabilitation:
  - Yes:
    - Days/week:
    - Type of rehabilitation:
      - Respiratory.
      - Cardiac.
      - Neurological.
      - Mobilisation.
  - No.

IV.- Consequences of COVID-19

1- Which of these symptoms have you experienced that you did not have before?

- Asthenia (lack of appetite):
- Anxiety:
- Depression:
- Stress in everyday situations:
- Memory loss:
- Headaches or migraines: Associated with exercise:
  - Yes
  - No

2- Can you live alone without any constant assistance from other staff, e.g. are you able to eat, walk, use the toilet and manage daily hygiene routines independently?

- Yes (if you tick yes, continue the survey in point 3)
- No (if no, answer the following question and skip to point 6)

2.1.- If you have marked that you cannot live without personal assistance, please indicate in which areas you need essential and constant help: (continue the survey in point 6)

- Eating
- Using the toilet
- Daily hygiene
- Walking
- Household chores
- Local travel

3.- Are there tasks/activities which he/she is no longer able to do by him/herself, he/she does not need constant help, but for some activities in his/her life he/she does need help.

- o Yes (if yes, answer the following question and skip to point 6)
- o No (if you tick no, continue the survey at point 4)

3.1.- If you have marked that there are tasks that you cannot do on your own, but you do not need constant help, mark which tasks you cannot do on your own and which you need to modify or help with:

- Local purchases
- Household chores
- Work
- Caring for loved ones

- ▪ What in particular can it no longer do in the same way?
- ▪ Why do you feel you can't do it?

4.- Do you need to avoid or reduce tasks/activities or extend these tasks in time? You can do the same as before, but with modifications or something specific you cannot do it.

- o Yes (if yes, answer the following question and skip to point 6)
- o No (if you tick no, continue the survey at point 5)

4.1.- If you have marked that you are autonomous, but you need to modify some tasks, please answer which ones.

- Home
- Work
- Social or leisure participation

▪ What in particular can it no longer do in the same way?
▪ Why do you feel you can't do it?

5.- Do you suffer from symptoms such as pain, depression or anxiety?

o Yes
o No

6.- Do you have symptoms through which it is necessary to avoid or reduce tasks? Do you have symptoms as a result of COVID-19, but not experiencing functional limitations?

o Yes
  ▪ What tasks does it do with limitations? ....
  ▪ What is the cause of the limitation? ....
o No

7.- Do you have trouble relaxing and/or do you see COVID-19 as a trauma?

o Yes
o No

8- From 1: strongly disagree, to 7: strongly agree indicate:

- My motivation is low when I am fatigued:
- Exercise makes me tired:
- I get fatigued easily:
- Fatigue interferes with my physical functioning:
- Fatigue causes me frequent problems:

- My fatigue prevents me from sustained physical activity:
- Fatigue is among my top three most disabling symptoms:
- Fatigue interferes with my work, family life or social life:

9.- Do you feel a lack of strength in your arms or legs when carrying out daily activities?

- Yes
    - In which activities? ....

- No

10.- Do you have pain during the day or at night?

- Yes
    - From 0=nothing to 10=the maximum imaginable, how much:
    - It prevents you from carrying out the usual activities you used to do:
        - Yes
        - No

- No

11.- What is the symptom/s that most affects your daily life, since you were diagnosed with COVID-19?

....

....

....

12.- In relation to the sequelae you have suffered from COVID-19, what do you consider to be the current need that needs to be addressed from the point of view of rehabilitation?

...

...

...

...

ANNEX V: Minute6 walk test.

You should walk, without running, the maximum distance for 6 minutes at a time. If you feel fatigued, you can stop as long as you need to.

PRUEBA DE MARCHA DE 6 MINUTOS (6MWT)

Nombre **Fecha** **Sexo (H/M)**

Edad (años) **Peso (Kg)** **Talla (m)** **FC máx [210-(edad x 0,65)]**

Diagnóstico **Examinador**

Medicación (incluir dosis y horario)

6MWT Nº1	30 metros			
		BASAL		FINAL
SaO2			(%)	
FC			(ppm)	
Disnea			(Borg)	
Fatiga EEII			(Borg)	
Tensión Arterial			mmHg	
Vueltas	Metros	Tiempo	SaO2	FC
1	30			
2	60			
3	90			
4	120			
5	150			
6	180			
7	210			
8	240			
9	270			
10	300			
11	330			
12	360			
13	390			
14	420			
15	450			
16	480			
17	510			
18	540			
19	570			
20	600			
21	630			
22	660			
23	690			
24	720			
25	750			
26	780			
27	810			

Incentivo	
min 1	*"Lo está haciendo muy bien, faltan 5 minutos"*
min 2	*"Perfecto, continúe así, faltan 4 minutos"*
min 3	*"Está en la mitad del tiempo de la prueba, lo está haciendo muy bien"*
min 4	*"Perfecto, continúe así, faltan dos minutos"*
min 5	*"Lo está haciendo muy bien, falta un minuto"*
min 5:45	*"Deberá detenerse cuando se lo indique"*
min6	***PARE***

PARADAS	TIEMPO AL PARAR	TIEMPO AL REANUDAR
1		
2		
3		

RECUPERACION			
	SaO 2	*FC*	*TA*
min1			
min2			
min3			
min4			
min5			

Distancia adicional	
Distancia total caminada	

ANNEX VI: 30-second sit-to-stand test.

Please, with your hands crossed over your shoulders and your feet flat on the floor, stand up completely and return to the starting position as many times as you can within 30 seconds.

I will indicate the start at the "go" signal and at the end I will say "stop".

ANNEX VII: 30-second elbow flexion test.

Please pick up the 2 kg dumbbell with your dominant hand, sitting with your feet flat on the floor. Bring the weight to your shoulder and return to the starting position as many times as you can within 30 seconds.

I will indicate the start at the "go" signal and at the end I will say "stop".

ANNEX VIII: Post-Covid functional status scale.

EVALUACIÓN DE LA ESCALA

**Nombre / ID paciente**	
**Fecha de diagnóstico de COVID-19**	
**Fecha de evaluación de la escala PCFS**	
**Lugar**	Al alta<br>Visita ambulatoria a las 4 semanas<br>Visita ambulatoria a las 8 semanas<br>Visita ambulatoria a los 6 meses<br>Otra (especifique) ________
**Encuestado**	Paciente<br>Paciente y otra persona<br>Sólo otra persona<br>Especifique ______
**Evaluador**	Médico<br>Personal del estudio

Please answer yes or no to the following questions.

ENTREVISTA ESTRUCTURADA

**1. SOBREVIDA**	**Calificación correspondiente en la escala PCFS si respuesta es "SI"**
**1.1 ¿Ha fallecido el paciente después del diagnóstico de COVID-19?**	**D**

**2. CUIDADO CONSTANTE**<br>*Explicación*: significa que alguien más debe estar a su disposición todo el tiempo. El cuidado puede ser proporcionada por un cuidador entrenado o no entrenado. El paciente generalmente estará postrado en la cama y puede tener incontinencia.	**Calificación correspondiente en escala PCFS si respuesta es "SI"**
**2.1 ¿Requiere usted cuidados contantes?**	**4**

**3. ACTIVIDADES BÁSICAS DE LA VIDA DIARIA (ABVD)**<br>*Explicación:* la asistencia incluye la ayuda física, instrucción verbal o supervisión de otra persona. Puede considerarse esencial cuando hay una necesidad de ayuda física (por parte de otra persona) con una actividad o para supervisión, o cuando el paciente necesita indicaciones o recordatorios para realizar una tarea. La necesidad de supervisión por razones de seguridad debería obedecer a un peligro objetivo que se presenta, y no "por si acaso".	**Calificación correspondiente en escala PCFS si respuesta es "SI"**
**3.1 ¿Es esencial la asistencia para comer?**<br>(Comer sin ayuda: la comida y los utensilios pueden ser proporcionados por otros)	**4**
**3.2 ¿Es esencial la asistencia para usar el baño?**<br>(Usar el baño sin ayuda: llegar al baño/inodoro; desvestirse lo suficiente; limpiarse; vestirse y salir)	**4**

**3.3 ¿Es esencial la asistencia para la rutina de higiene diaria?**<br>(La rutina de higiene incluye sólo lavarse la cara, peinarse s, lavarse los dientes y colocarse la dentadura postiza. Los implementos pueden ser proporcionados por otros sin considerar esto como asistencia)	**4**
**3.4 ¿Es esencial la asistencia para caminar?**<br>(Caminar sin asistencia: si es absolutamente necesario, es capaz de caminar en el interior o alrededor de la casa o sala, puede utilizar cualquier ayuda, sin embargo, no requiere ayuda física o instrucción verbal o supervisión de otra persona)	**4**

**4. ACTIVIDADES INSTRUMENTALES DE LA VIDA DIARIA (AIVD)**<br>*Explicación*: la asistencia incluye la ayuda física, instrucción verbal o supervisión de otra persona. Puede considerarse esencial cuando hay una necesidad de ayuda física (por parte de otra persona) con una actividad o para supervisión, o cuando el paciente necesita indicaciones o recordatorios para realizar una tarea. La necesidad de supervisión por razones de seguridad debería obedecer a un peligro objetivo que se presenta, y no "por si acaso".	**Calificación correspondiente en escala PCFS si respuesta es "SI"**
**4.1 ¿Es esencial la asistencia para las tareas domésticas básicas que son importantes para la vida diaria?**<br>(Por ejemplo: preparar una comida sencilla, lavar los platos, sacar la basura; excluya tareas que no necesitan ser realizadas todos los días)	**4**
**4.2 ¿Es esencial la asistencia para los viajes locales?**<br>(Viajes locales sin asistencia: el paciente puede conducir o utilizar el transporte público para desplazarse. La posibilidad de utilizar un taxi es suficiente, siempre que el paciente pueda llamar e indicarle al conductor)	**4**
**4.3 ¿Es esencial la asistencia para las compras locales?**<br>(El paciente no es capaz de comprar alimentos o artículos de primera necesidad por sí mismo)	**3**

**5. PARTICIPACIÓN EN ROLES SOCIALES HABITUALES**<br>*Explicación:* esta sección se refiere al disminución en el cumplimiento de los principales roles sociales (no las circunstancias sociales o financieras).	**Calificación correspondiente en escala PCFS si respuesta es "SI"**
**5.1 ¿Es esencial adaptar las tareas/actividades en el hogar o en el trabajo/estudio porque usted no puede realizarlas por sí mismo (por ejemplo, produciendo un cambio en el nivel de responsabilidad, un cambio de de tiempo completo a tiempo parcial en el trabajo, o un cambio en la educación)?**<br>(El trabajo se refiere tanto al empleo remunerado como al trabajo voluntario. Las adaptaciones especiales que permiten a alguien volver a trabajar, aunque normalmente no podría hacerlo, deben considerarse como una adaptación del trabajo).	**3**
**5.2 ¿Necesita usted ocasionalmente evitar o reducir las tareas/actividades en el hogar o en el trabajo/estudio o necesita extenderlas a lo largo del tiempo (aunque básicamente usted sea capaz de realizar todas esas actividades)?**	**2**
**5.3 ¿Ya no puede cuidar bien de sus seres queridos como antes?**<br>(Cuidar bien incluye cuidar a su pareja, padres, nietos u otras personas dependientes).	**3**
**5.4 Desde el diagnóstico de COVID-19, ¿ha habido problemas en sus relaciones o se ha aislado?**<br>(Estos problemas incluyen problemas de comunicación, dificultades en las relaciones con las personas en casa o en el trabajo/estudio, pérdida de amistades ,(aumento del) aislamiento, etc.)	**3**
**5.5 ¿Está restringida su participación en actividades sociales y de ocio?**<br>(Incluye pasatiempos e intereses, incluyendo ir a un restaurante, bar, cine, salir a caminar, juegos, lectura de libros, etc.)	**2**

**6. LISTA DE CHEQUEO DE SÍNTOMAS**<br>*Explicación:* estos pueden ser cualquier síntoma o problema informado por los pacientes o encontrado en el examen físico. Los síntomas incluyen, pero no se limitan a: disnea, dolor, fatiga, debilidad muscular, pérdida de memoria, depresión y ansiedad.	**Calificación correspondiente en escala PCFS si respuesta es "SI"**
**6.1 ¿Presenta usted síntomas por los cuales se deben evitar, reducir o extender las tareas/actividades habituales a lo largo del tiempo?**	**2**
**6.2 ¿Presenta usted algún síntoma, resultante de COVID-19, sin experimentar limitaciones funcionales?**	**1**
**6.3 ¿Tiene usted problemas para relajarse o experimenta el COVID-19 como un trauma?**<br>('Trauma' es definido como: sufrir recuerdos intrusivos, recuerdos recurrentes o respuestas evitativas, asociadas a haber experimentado el COVID-19.)	**1**

Asignación de la calificación en la escala de estado funcional post-COVID-19

La clasificación general es simplemente el peor estado funcional indicado por las respuestas del paciente (la calificación más alta corresponde a mayores limitaciones). Si un paciente no tiene limitaciones o síntomas, entonces la calificación correspondiente en la escala es 0.

**Calificación final de la Escala PCFS: _______**

¿Cuál era su grado de escala PCFS antes del COVID-19? ________

Figura 1: Flujograma para auto reporte del paciente de la Escala de estado funcional post COVID19

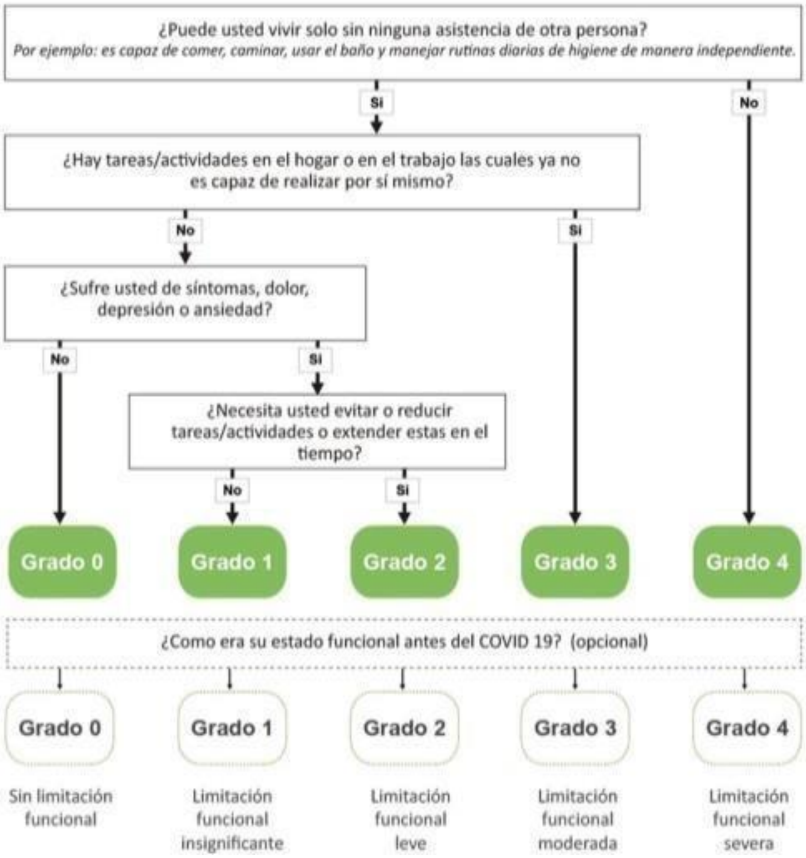

ANNEX IX: Abbreviated general self-efficacy scale.

Please rate from 1= never happens to 5= always happens to you, the following statements:

Item
1.A pesar de los obstáculos yo puedo encontrar las maneras de obtener lo que quiero
2. Yo enfrento problemas graves si me esfuerzo lo suficiente
3. Es fácil mantenerme en mis metas hasta alcanzarlas
4. Me siento seguro de poder enfrentar eficazmente situaciones inesperadas
5. Gracias a mis cualidades personales yo puedo enfrentar situaciones inesperadas
6. Yo puedo mantener la calma cuando estoy en problemas porque confío en mis habilidades para enfrentarlos
7. Venga lo que venga, por lo general soy capaz de manejarlo
8. Yo puedo resolver la mayoría de los problemas si me esfuerzo lo necesario
9. En una situación dificil, generalmente se me ocurre lo que debo hacer
10. Cuando me enfrento a un problema, generalmente encuentro varias soluciones

ANNEX X: Depression and anxiety scale.

DASS-21

Por favor lea las siguientes afirmaciones y coloque un círculo alrededor de un número (0, 1, 2, 3) que indica en qué grado le ha ocurrido a usted esta afirmación *durante la semana pasada*. La escala de calificación es la siguiente:

0: No me ha ocurrido; 1: Me ha ocurrido un poco, o durante parte del tiempo; 2: Me ha ocurrido bastante, o durante una buena parte del tiempo; 3: Me ha ocurrido mucho, o la mayor parte del tiempo.

1.	Me ha costado mucho descargar la tensión	0	1	2	3
2.	Me di cuenta que tenía la boca seca	0	1	2	3
3.	No podía sentir ningún sentimiento positivo	0	1	2	3
4.	Se me hizo difícil respirar	0	1	2	3
5.	Se me hizo difícil tomar la iniciativa para hacer cosas	0	1	2	3
6.	Reaccioné exageradamente en ciertas situaciones	0	1	2	3
7.	Sentí que mis manos temblaban	0	1	2	3
8.	He sentido que estaba gastando una gran cantidad de energía	0	1	2	3
9.	Estaba preocupado por situaciones en las cuales podía tener pánico o en las que podría hacer el ridículo	0	1	2	3
10.	He sentido que no había nada que me ilusionara	0	1	2	3
11.	Me he sentido inquieto	0	1	2	3
12.	Se me hizo difícil relajarme	0	1	2	3
13.	Me sentí triste y deprimido	0	1	2	3
14.	No toleré nada que no me permitiera continuar con lo que estaba haciendo....	0	1	2	3
15.	Sentí que estaba al punto de pánico	0	1	2	3
16.	No me pude entusiasmar por nada...	0	1	2	3
17.	Sentí que valía muy poco como persona	0	1	2	3
18.	He tendido a sentirme enfadado con facilidad	0	1	2	3
19.	Sentí los latidos de mi corazón a pesar de no haber hecho ningún esfuerzo físico	0	1	2	3
20.	Tuve miedo sin razón	0	1	2	3
21.	Sentí que la vida no tenía ningún sentido...	0	1	2	3

ANNEX XI: Questionnaire on behavioural regulation in sport

Cuestionario de Regulación de Conducta en el Deporte (BRSQ) Lonsdale, Hodge, y Rose (2008)

**Participo en este deporte…**	Nada es verdad	Bastante en desacuerdo	Algo en desacuerdo	Neutro	Algo de verdad	Bastante de verdad	Muy verdadero
Porque lo disfruto	❶	❷	❸	❹	❺	❻	❼
Por el placer que me da el conocer más acerca de este deporte	❶	❷	❸	❹	❺	❻	❼
Porque me encantan los estímulos intensos que puedo sentir mientras practico este deporte	❶	❷	❸	❹	❺	❻	❼
Porque disfruto cuando intento alcanzar metas a largo plazo	❶	❷	❸	❹	❺	❻	❼
Porque es parte de lo que soy	❶	❷	❸	❹	❺	❻	❼
Porque los beneficios del deporte son importantes para mi	❶	❷	❸	❹	❺	❻	❼
Porque me sentiría avergonzado si lo abandono	❶	❷	❸	❹	❺	❻	❼
Porque si no lo hago otros no estarían contentos conmigo	❶	❷	❸	❹	❺	❻	❼
Sin embargo, no se por qué lo hago	❶	❷	❸	❹	❺	❻	❼
Porque me gusta	❶	❷	❸	❹	❺	❻	❼
Porque me gusta aprender cómo usar nuevas técnicas	❶	❷	❸	❹	❺	❻	❼
Por el entusiasmo que siento cuando estoy implicado en la actividad	❶	❷	❸	❹	❺	❻	❼
Porque disfruto mientras trabajo algo importante	❶	❷	❸	❹	❺	❻	❼
Porque es una oportunidad de ser quien soy	❶	❷	❸	❹	❺	❻	❼
Porque me enseña disciplina	❶	❷	❸	❹	❺	❻	❼
Porque me sentiría haber fallado si lo abandono	❶	❷	❸	❹	❺	❻	❼
Porque otros me presionan a jugar	❶	❷	❸	❹	❺	❻	❼
Sin embargo, me cuestiono por qué continuo	❶	❷	❸	❹	❺	❻	❼
Porque me divierto	❶	❷	❸	❹	❺	❻	❼
Porque disfruto aprendiendo nuevas técnicas	❶	❷	❸	❹	❺	❻	❼
Por el placer que me da cuando estoy totalmente entregado en este deporte	❶	❷	❸	❹	❺	❻	❼
Porque disfruto mientras hago algo lo mejor que puedo	❶	❷	❸	❹	❺	❻	❼
Porque el practicar este deporte es parte de quien soy	❶	❷	❸	❹	❺	❻	❼
Porque aprecio los beneficios de este deporte	❶	❷	❸	❹	❺	❻	❼
Porque me siento obligado a continuar	❶	❷	❸	❹	❺	❻	❼
Porque otros me empujan a jugar	❶	❷	❸	❹	❺	❻	❼
Sin embargo, las razones de por qué practico no las tengo claras	❶	❷	❸	❹	❺	❻	❼
Porque lo encuentro agradable	❶	❷	❸	❹	❺	❻	❼
Me gusta aprender cosas nuevas acerca de este deporte	❶	❷	❸	❹	❺	❻	❼
Por los sentimientos positivos que siento mientras practico este deporte	❶	❷	❸	❹	❺	❻	❼
Porque me produce satisfacción cuando me esfuerzo por alcanzar mis metas	❶	❷	❸	❹	❺	❻	❼
Porque me permite vivir de acuerdo con mis valores	❶	❷	❸	❹	❺	❻	❼
Porque es una manera muy buena de aprender cosas que pueden ser de gran utilidad en mi vida diaria	❶	❷	❸	❹	❺	❻	❼
Porque me sentiría culpable si lo abandono	❶	❷	❸	❹	❺	❻	❼
Para satisfacer a los que quieren que juegue	❶	❷	❸	❹	❺	❻	❼
Sin embargo, me pregunto por qué me esfuerzo para esto	❶	❷	❸	❹	❺	❻	❼

ANNEX XII: Multidimensional scale of perceived social support

ESCALA MULTIDIMENSIONAL DE APOYO SOCIAL PERCIBIDO (EMAS)

Lee cada una de las siguientes frases cuidadosamente. Indica tu acuerdo con cada una de ellas empleando esta escala:

1	2	3	4	5	6	7
Totalmente en desacuerdo	Bastante en desacuerdo	Más Bien en desacuerdo	Ni de acuerdo ni en desacuerdo	Más bien de acuerdo	Totalmente en desacuerdo	Totalmente de Acuerdo

1.	Hay una persona que está cerca cuando estoy en una situación difícil.	1	2	3	4	5	6	7
2.	Existe una persona especial con la cual yo puedo compartir penas y alegrías.	1	2	3	4	5	6	7
3.	Mi familia realmente intenta ayudarme	1	2	3	4	5	6	7
4.	Obtengo de mi familia la ayuda y el apoyo emocional que necesito.	1	2	3	4	5	6	7
5.	Existe una persona que realmente es una fuente de bienestar para mí.	1	2	3	4	5	6	7
6.	Mis amigos realmente tratan de ayudarme	1	2	3	4	5	6	7
7.	Puedo contar con mis amigos cuando las cosas van mal	1	2	3	4	5	6	7
8.	Yo puedo hablar de mis problemas con mi familia	1	2	3	4	5	6	7
9.	Tengo amigos con los que puedo compartir las penas y alegrías	1	2	3	4	5	6	7
10.	Existe una persona especial en mi vida que se preocupa por mis sentimientos	1	2	3	4	5	6	7
11.	Mi familia se muestra dispuesta a ayudarme para tomar decisiones	1	2	3	4	5	6	7
12.	Puedo hablar de mis problemas con mis amigos.	1	2	3	4	5	6	7

ANNEX XIII: Quality of Life Questionnaire.

CUESTIONARIO DE SALUD SF-12

INSTRUCCIONES: Las preguntas que siguen se refieren a lo que usted piensa sobre su salud. Sus respuestas permitirán saber como se encuentra usted y hasta qué punto es capaz de hacer sus actividades habituales.

Por favor, conteste cada pregunta marcando una casilla. Si no está seguro/a de cómo responder a una pregunta, por favor, conteste lo que le parezca más cierto.

1. En general, usted diría que su salud es:

1	2	3	4	5
❑	❑	❑	❑	❑
**Excelente**	**Muy buena**	**Buena**	**Regular**	**Mala**

Las siguientes preguntas se refieren a actividades o cosas que usted podría hacer en un día normal. Su salud actual, ¿le limita para hacer esas actividades o cosas? Si es así, ¿cuánto?

	1<br>**Sí, me limita mucho**	2<br>**Sí, me limita un poco**	3<br>**No, no me limita nada**
2. **Esfuerzos moderados,** como mover una mesa, pasar la aspiradora, jugar a los bolos o caminar más de 1 hora	❑	❑	❑
3. Subir **varios** pisos por la escalera	❑	❑	❑

Durante **las 4 últimas semanas,** ¿ha tenido alguno de los siguientes problemas en su trabajo o en sus actividades cotidianas, **a causa de su salud física?**

	1<br>**Sí**	2<br>**No**
4. ¿**Hizo menos** de lo que hubiera querido hacer?	❑	❑
5. ¿Tuvo que **dejar de hacer algunas tareas** en su trabajo o en sus actividades cotidianas?	❑	❑

Durante las **4 últimas semanas,** ¿ha tenido alguno de los siguientes problemas en su trabajo o en sus actividades cotidianas, **a causa de algún problema emocional** (como estar triste, deprimido, o nervioso)?

	1<br>**Sí**	2<br>**No**
6. ¿**Hizo menos** de lo que hubiera querido hacer, **por algún problema emocional**?	❑	❑
7. ¿No hizo su trabajo o sus actividades cotidianas tan **cuidadosamente** como de costumbre, **por algún problema emocional**?	❑	❑

8. Durante las **4 últimas semanas,** ¿hasta qué punto **el dolor** le ha dificultado su trabajo habitual (incluido el trabajo fuera de casa y las tareas domésticas)?

1	2	3	4	5
❑	❑	❑	❑	❑
**Nada**	**Un poco**	**Regular**	**Bastante**	**Mucho**

Las preguntas que siguen se refieren a cómo se ha sentido y cómo le han ido las cosas durante las **4 últimas semanas.** En cada pregunta responda lo que se parezca más a cómo se ha sentido usted. Durante las **4 últimas semanas** ¿cuánto tiempo...

	1<br>**Siempre**	2<br>**Casi siempre**	3<br>**Muchas veces**	4<br>**Algunas veces**	5<br>**Sólo alguna vez**	6<br>**Nunca**
**9.** ...se sintió calmado y tranquilo?	☐	☐	☐	☐	☐	☐
**10.** ...tuvo mucha energía?	☐	☐	☐	☐	☐	☐
**11.** ...se sintió desanimado y triste?	☐	☐	☐	☐	☐	☐

12. Durante las **4 últimas semanas,** ¿con qué frecuencia la salud física o los problemas emocionales le han dificultado sus actividades sociales (como visitar a los amigos o familiares)?

1	2	3	4	5
☐	☐	☐	☐	☐
**Siempre**	**Casi**	**Algunas siempre**	**Sólo veces**	**Nunca alguna vez**

ANNEX XIV: Activity adherence monitoring diary (to measure adherence and commitment variables)

Weekly	Sem 1			Sem 2			Sem 3		
Diary	1	2	3	1	2	3	1	2	3
I have done all the exercises as instructed									
I have done all, but not all the repetitions.									
I have done some complete exercises as instructed									
I have done some exercises and not all the repetitions.									
I have not done them									
I have done more voluntary exercise than recommended									
Walking more than 30 minutes									
Running more than 30 minutes									
Additional exercises:									
Describe which									

ANNEX XV: Analysis of satisfaction with the programme and the platform.

- Please rate from 0 to 4 your overall satisfaction, where 0=very dissatisfied and 4=very satisfied, with the following aspects:

- The exercise programme...........
- The attention received during the programme............

- Experimental group:

- The Hefora platform ....
- The exercise videos.................
- The explanations of the exercises...............
- The educational videos..................

- Please let me know if you have had any problems with the exercise programme during these 15 days:
    - Yes
        - Have you been able to solve it?
            - Yes
            - No
        - Has it prevented you from doing the programme?
            - Yes
            - No
    - No

- Control group:

- The explanatory brochure..............

ANNEX XVI: Patient Information and Informed Consent Form

PARTICIPANT INFORMATION DOCUMENT

Title of the research: Effectiveness of an asynchronous telerehabilitation programme in post-COVID-19 patients: feasibility study.<br><br>Promoter: San Jorge University.<br><br>Principal Investigator: Sandra Calvo Carrión and Carolina Jiménez Sánchez Tel: 661 833 194 / 649 612 644 mail: scalvo@usj.es / cjimenez@usj.es<br><br>Centre: San Jorge University - Nuestra Señora de Gracia Provincial Hospital of [illegible]

1. Introduction:

We are writing to you to request your participation in a research project that we are carrying out from San Jorge University in collaboration with Nuestra Señora de Gracia Provincial Hospital and Royo Villanova Hospital in Zaragoza. Your participation is voluntary, but this study is important to obtain the knowledge we need. This project has been approved by the Ethics Committee of Aragon. Before making a decision it is necessary that you please:

decision it is necessary that you please:

- read this entire document
- understand the information contained in the document
- ask as many questions as you consider necessary
- make a considered decision
- sign the informed consent form, if you finally wish to participate

If you choose to participate, you will be given a copy of this sheet and the signed consent form. Please keep it in case you need it in the future.

2. Why are you being asked to participate?

Your cooperation is requested because you have suffered from COVID-19 and have been admitted to the Hospital Provincial Nuestra Señora de Gracia or to the Hospital Royo Villanova de Zaragoza and you will be offered, upon your discharge, to participate in a pilot study where a home programme will be carried out consisting of a series of exercises and therapeutic recommendations for you to improve your physical condition and quality of life.

A total of 32 patients previously admitted to and discharged from the Hospital Provincial Nuestra Señora de Gracia in Zaragoza will participate in the study.

3. What is the subject of this study?

The study will allow us to assess whether the prescription of an exercise and health education programme carried out at home using a web application or written information helps to improve the physical condition and quality of life of people who have undergone COVID-19 and have been discharged from hospital.

4. What do I have to do if I decide to participate?

The programme will consist of an exercise and health education programme for patients with post-COVID19 sequelae. The intervention group will receive an exercise programme and educational recommendations through the HEFORA web platform offered free of charge by the Professional Association of Physiotherapists of Aragon. The control group will receive the same exercise programme and educational recommendations in the form of an explanatory leaflet with information on how to follow the programme.

It is required to evaluate your clinical history, which is located at the Hospital Provincial Nuestra Señora de Gracia in Zaragoza). If you wish to participate, meet the inclusion criteria and sign the informed consent form, you will be summoned to the Hospital Provincial Nuestra Señora de Gracia for a physical assessment, which you will have to repeat after 12 weeks at the end of the study (**two assessments lasting** approximately 30 minutes each). It is important for you to know that you will have to travel to the Hospital Provincial Nuestra Señora de Gracia for these two assessments, and that these trips (2 in total: one at the beginning of the intervention and one immediately after its completion) will not be compensated. This study is not funded, so these trips cannot be covered).

In addition, during the duration of the study, you will be asked to complete a diary, either in

writing or online via a web platform, in which you will record whether you have completed the programmed exercises, whether you have performed them with the indicated load (weight and repetitions), whether you have performed additional exercise to that prescribed and your level of fatigue (from 0 to 10) at the end of the exercises.

There will also be a **telephone check-up every two weeks** to solve possible problems with the implementation, to assess the level of fatigue during the programme and to analyse possible incidents.

The programme will last for a total of 13 weeks from the time you are assigned to a group and make the first assessment until the final assessment and for months6 after the two follow-ups. All assessment, intervention and follow-up sessions will be free of charge for all participants. The only cost borne by the participant will be the cost of travel to the Hospital Provincial Nuestra Señora de Gracia in Zaragoza where the face-to-face assessments will be carried out.

5. What risks or inconvenience does it pose?

The implementation of a therapeutic exercise and health education programme has no adverse effects if it is carried out according to the recommendations. Each patient will have the telephone number of the principal investigators in case of any incident and will be able to communicate by telephone with the investigators for any doubt or adverse circumstance during the treatment. The HEFORA web platform also has a messaging channel where any circumstance of this nature can be reported.

6. Will I get any benefits from my participation?

As this is a research study aimed at generating knowledge, you will not receive any profit for your participation, but you will contribute to scientific progress and social benefit. You will not receive any financial compensation for your participation.

7. **How will my personal data be processed?**

Basic information on data protection. Data controller: San Jorge University.

Purpose: Your personal data will be processed exclusively for the research work referred to in this document.

Legitimation: The processing of the data in this study is legitimised by your consent to participate.

Recipients: No data will be passed on to third parties unless legally obliged to do so. Rights: As a participant in the study you may exercise your rights of access, modification, opposition, cancellation, limitation of processing and portability by contacting the Data Protection Delegate of the University attaching a photocopy of your ID card or equivalent to your request to exercise your rights to the registered office of USJ located at Autovía A-23 Zaragoza- Huesca, km. 50830-299, Villanueva de Gállego (Zaragoza), or the email address privacidad@usj.es. As a user of the HEFORA platform, you have the right to access, rectify and delete the data, as well as other rights indicated in the additional information, by sending an e-mail to info@hefora.com. The platform will anonymously and encrypted collect the number of times you have accessed the platform and the number of times you have viewed the videos. This data will be stored until the end of the study, when it will be transferred to the principal investigators for analysis, solely for research purposes and maintaining the anonymity of the participants at all times.

Data collection will be carried out through the paper diaries and through the HEFORA web application (you can consult the full privacy policy at https://es.hefora.com/privacy-and-cookie-policies "privacy policy").

When accessing the platform for the first time, you must tick the box to check whether you accept the platform's privacy and data protection policy.

The HEFORA platform first collects some initial data to create a user account, which are a username, password, email address and name and surname. Once the patient has created their account, they contact the researcher and digitally accept the authorisation document for the processing of personal data in the study and once this link has been established, the patient is asked to provide a username and password.

The researcher can include in the platform the data collected directly by the researcher or access the results of the questionnaires self-administered by the patient (data described in the research project). The date and time of signature is recorded and can be consulted by both the patient and the researcher

The data that may be collected on the platform after this acceptance, if the user decides to fill them in, will be, depending on the user (researcher/professional or patient):

Professionals: DNI/NIF, Name and surname, postal or e-mail address, telephone, image, manual signature, personal characteristics, social, academic and professional circumstances, employment details, commercial, economic, financial and insurance information, transactions of goods and services.

Users: DNI/NIF, Name and surname, postal or e-mail address, telephone, image, manual signature, personal characteristics (personality or behaviour), personal characteristics, social, academic and professional circumstances, employment details, commercial, economic, financial and insurance information, transactions of goods and services.

Patients: DNI/NIF, Name and surname, postal or e-mail address, telephone, image, manual signature, physical marks, health, personal characteristics (personality or behaviour), personal characteristics, social circumstances, academic and professional, employment details, commercial, economic, financial and insurance information, transactions of goods and services.

2.- Regarding the data kept, for the duration of the investigation, the data kept will be those indicated above. If the service is terminated, the data will be deleted except for those that must be kept due to legal obligations. And for further use, research, these data are anonymised and therefore are not personal. The user has the possibility to delete his account also after the end of the study.

3.- The Privacy Policy is the one that appears on the website hefora.net and only the researchers have access to the data, with the exception of name and surname and email which can be accessed by the company that owns Hefora, which does not pass this data on to third parties.

4. Regarding security measures, the platform is GDPR compliant, and has the following security measures in place:

- At the server level, the server that manages hefora.net is hosted in a private data centre with cloud technology on OpenStack. The provider has no access to the server system or its data. The snapshots taken are stored in another data centre on the same premises and are transferred via an isolated internal network. The administration of the server is done under the OpenSSH service with authentication by password or RSA private key. The communication between the user and the web service is carried out under SSL with an EV certificate (SectigoRSA Extended Validation Secure Server CA). The communication between the web services and the database is not exposed to the outside and is reduced to its isolated internal network managed by Debian system services. Data 9.9.backups are encrypted at the system level underAES-256 and transferred with OpenSSH over the internal network under VLAN to another backup server. Like the hefora.net server, this backup server runs with cloud technology on OpenStack and the provider also has no access to the system. The administration of this server is also carried out under the OpenSSH service

with password or RSA private key authentication.

- At the database level, all personal and medical data are stored with record-level encryption, which means that everything is incomprehensible without first being decrypted. In addition, medical records are properly pseudo-anonymised so that it is not possible to directly link the natural person and his or her medical data. In addition, all documents exchanged with users (e.g. videos) are encrypted through an external security provider, chino.io, which is ISO certified.27001.
- Regarding the export of questionnaires: for each questionnaire and the person who filled it in, what is done is to generate an encrypted identifier from the original identifier of that person, so that even if you had access to the original database, you could not know which original identifier it refers to, and therefore you could not know which person filled in each questionnaire. In this way, with the exported list of questionnaires, it is possible to know if 2 different questionnaires belong to the same person (they will have the same encrypted identifier), but it is not possible to know which person filled in which questionnaire.
- As for the specific process of pseudo-anonymisation, what has been done is to break the link in databases between the patient and medical data tables. There is no identifier to indicate which patient each medical record corresponds to; they are independent tables. In another separate table, also unrelated to the previous ones, what is being done is to store the patient's identifier encrypted with an AES-256 algorithm and their personal data also encrypted with the same algorithm. In order to know to which individual a medical record corresponds, this identifier would have to be decrypted, so there is no way, if you have access to the database, to establish the relationship between patient and medical record without knowing the decryption key and algorithm. Moreover, no personally identifiable data is stored in the medical history table. The result is that the medical records are pseudo-anonymised, as their information does not contain any personal data and no direct relationship with the patient can be established. The same applies to the rest of the data related to the clinical history: questionnaires, prescriptions, etc.

Likewise, in compliance with the provisions of the RGPD, you are informed that, if you so wish, you may go to the Data Protection Agency (https://www.aepd.es) to file a complaint when you consider that your rights have not been duly addressed.

The processing of your personal data will be carried out using techniques to maintain your anonymity through the use of random codes, so that your personal identity is completely hidden during the research process.

Based on the results of the research work, scientific communications may be prepared for presentation at congresses or in scientific journals, but they will always be done with grouped data and nothing that could identify the researcher will ever be disclosed.

The data from the face-to-face interviews and assessments will be processed by entering these results on paper into a data processing programme.

8. Who is funding the study?

This project has no external funding.

9. Will I be informed of the results of the study?

You have the right to know the results of this study, both the general results and those derived from your specific data. You also have the right not to know the results of this study if you wish to do so. For this reason, we will ask you in the informed consent document which option you prefer. If you do wish to know the results, the researcher will send you the results.

10. Can I change my mind?

Your participation is completely voluntary, you can decide not to participate or to withdraw from the study at any time without having to give explanations and without this having any impact on your health care. You only need to tell the principal investigator of the study your intention.

11. What happens if I have questions during my participation?

The name and contact telephone number of the researchers responsible for the study are listed on the first page of this document. If you have any questions about your participation, please do not hesitate to contact them.

Thank you very much for your attention, if you finally wish to participate, please sign the attached consent form.

ANNEX XVII: INFORMED CONSENT DOCUMENT

PROJECT TITLE: Effectiveness of an asynchronous telerehabilitation programme in post-COVID-19 patients: feasibility study.

.... I,(participant's nameand surname)

I have read the information sheet provided to me.

I have been able to ask questions about the study and have received sufficient information about it.

I have spoken to:(nameand surname of researcher)

I understand that my participation is voluntary.

I understand that I may withdraw from the study:

1) whenever you want
2) without having to explain
3) without affecting my medical care.

I freely give my consent to participate in this study and consent to the access and use of my data as stipulated in the information sheet provided to me (also for the genetic analysis to be carried out - if applicable).

I wish to be informed about the results of the study: yes no (please tick as appropriate)

If applicable: I agree that my clinical data may be reviewed by personnel outside the centre for the purposes of the study, and I am aware that this consent is revocable.

I have received a signed copy of this Informed Consent.

Signature of the participant:

Date:

I have explained the nature and purpose of the study to the above-mentioned patient.

Investigator's signature:

Date:

ANNEX XVIII: Data protection policy HEFORA platform.

PRIVACY POLICY

**Basic information on the Protection of**	
**Responsible:**	FISIO CONSULTORES, S.L.
**Purpose:**	1. To be able to offer you the services and utilities offered by our HEFORA platform. As well as to inform you about our services and/or products and to send you commercial communications of interest to you.<br>2. Conduct scientific research and statistical studies.
**Legitimation:**	- Execution of a contract (use of the platform)<br>- Consent of the data subject (processing of health data and scientific research and statistical studies).

**Target group:**	The only disposals envisaged are:<br>- The one carried out at the moment of contracting the services of a professional of the platform, since your existing data in the platform will become visible and the responsibility of the professional for a different purpose, such as providing you with the healthcare service.<br><br>- Those carried out by legal obligation.<br><br>There are Data Processors outside the European Union, under the Privacy Policy.<br>Shield".
**Rights:**	You have the right to access, rectify and delete the data, as well as other rights, indicated in the additional information, which you can exercise at info@hefora.com.
**Provenance:**	From the interested party himself or from his legal representative.
**Additional information:**	Additional detailed information on Data Protection can be found on our website: https://hefora.com/wp-content/uploads/2018/08/Politica_._de_PRIVACITY.pdf

1. USER INFORMATION

FISIO CONSULTORES, S.L. hereinafter "Responsible" or "Service", and as a Spanish trading company with registered office at Calle Magallón, 12- 50420de Cadrete (Zaragoza) and NIF: B-99396947 is the Responsible, or Charged where appropriate depending on their profile, for the processing of personal data of users of the platform and informs you that these data will be treated in accordance with the provisions of Regulation (EU) 2016/679 of 27 April 2016 (GDPR) on the protection of natural persons with regard to the processing of personal data and the free movement of such data, for which you are provided

with the following processing information:

PROFILE AND DATA	HEFORA processes your data as:
**Professional** (identification , professional and invoicing data, among others).	**Data Controller**

**Patient invited by Professional** (data as a patient of the professional and registration data on the platform, in addition to health-related data)	**Data Processor**
**Potential patient** (registration data and platform profile. Including possible health data you enter).	**Data Controller**

Purposes of processing:

1.- ***To maintain a contractual relationship with the User through the platform in order to provide the service***. The operations envisaged to carry out the processing are:

· The collection and storage of personal data for the provision of the services described on the platform. So that you can manage your profile, keep track and receive physiotherapy health services with the help of the platform. Such processing may be carried out by the Data Controller in its capacity as such or by the Data Processor depending on the user profile concerned.

· To process orders, requests or any type of request made by the user through any of the forms of contact made available to them.

2.- ***The sending of commercial advertising communications via e-mail, social communities or any other electronic or physical medium, present or future, that enables commercial communications to be made***. The operations envisaged to carry out the processing are:

· The same as those established for the use of the platform. These communications will be made by the RESPONSIBLE and related to its products and/or services, or those of its collaborators or suppliers with whom it has reached a promotional agreement. In this case, third parties will never have access to the personal data of the users of the platform.

-

And, if available, the newsletter of the website.

3.- ***The carrying out of scientific research and statistical studies after carrying out and applying the corresponding techniques for the anonymisation of personal data, these being irreversible.***

Legal basis for the different treatments: As appropriate,

· The contract with the interested party through the Terms and Conditions of Use of the platform and this Privacy Policy to enjoy the services of the platform.
· The legitimate interest of the Data Controller, for the sending of commercial communications.
· The consent of the data subject, for the processing of health data and scientific research and statistical studies.

Data retention criteria: data shall be retained for as long as the contract remains in force and/or there is a mutual interest in maintaining the purpose of the processing. When it is no longer necessary for that purpose, it shall be deleted with appropriate security measures to ensure pseudonymisation and/or anonymisation of the data or total destruction of the data.

Communication of data: We do not sell your personal data to other companies. The only transfers envisaged are:
- The one carried out at the moment in which, once registered on the platform, you decide to contract the services of a professional on the platform, as your existing data on the platform will become visible and the responsibility of the professional for a purpose other than that of accessing the platform, such as providing you with the corresponding healthcare service.
- Those carried out by legal obligation.

There are Data Processors outside the European Union, under PrivacyShield.

Rights of the User:

- Right to withdraw consent at any time.
- The right of access, rectification, portability and deletion of your data and the right to limit or oppose its processing.
- The right to lodge a complaint with the supervisory authority (www.agpd.es) if you consider that the processing does not comply with the regulations in force.

Contact details for exercising your rights:

You may at any time exercise your rights of access, rectification, suppression, limitation of processing, opposition and portability of your personal data by sending an e-mail to: info@hefora.com or to the postal address: FISIO CONSULTORES, S.L. Calle Magallón, - 12Cadrete50420 (ZARAGOZA).

In both cases you must identify yourself with your name and surname, as well as a copy of your ID card.

Where you have given consent for a specific purpose, you have the right to withdraw your consent at any time, without affecting the lawfulness of the processing based on your consent prior to its withdrawal.

In addition, if as a user of the platform you consider that there is a problem with the way in which the Data Controller is handling your data, you can address your complaints to the DPD or the corresponding data protection authority, being the Spanish Data Protection Agency the indicated one in the case of Spain.

This document sets out the conditions of collection, processing and use of personal and non-personal information of users.

2. INFORMATION PROVIDED BY THE USER AND ITS MANDATORY OR OPTIONAL NATURE

Users, by ticking the corresponding boxes and entering data in the fields marked with an asterisk (*) in the contact form or presented in download forms, expressly and freely and unequivocally accept that their data are necessary to meet their request, by the provider, being voluntary the inclusion of data in the remaining fields. The User guarantees that the personal data provided to the RESPONSIBLE are truthful and is responsible for communicating any changes to them.

Completion of the forms is voluntary and, if the mandatory fields are not filled in, some of the platform's functions may not be executed or may be limited.

The RESPONSIBLE expressly informs and guarantees users that their personal data will not be transferred under any circumstances to third parties, and that whenever any type of transfer of personal data is carried out, the express, informed and unequivocal consent of the Users will be requested beforehand.

The personal data we will collect will depend on how you use the platform.
We will collect this data automatically - through cookies or similar, with your consent. Visit our **Cookie Policy**; the IP address of your connection and data about your device and connection, among others-, those that you voluntarily provide us with -through registration on the platform, use, messages sent, newsletter subscription, among others- and those provided to us by third parties - such as social networks-.

3. SECURITY MEASURES

That in accordance with the provisions of the applicable personal data protection regulations, the RESPONSIBLE is complying with all the provisions of the GDPR regulations for the processing of personal data under its responsibility, and manifestly with the principles described above

in Article 5 of the GDPR, whereby they are processed lawfully, fairly and in a transparent manner in relation to the data subject and are adequate, relevant and limited to what is necessary in relation to the purposes for which they are processed.

The RESPONSIBLE PARTY warrants that it has implemented appropriate technical and organisational policies to implement the security measures set out in the GDPR to protect the rights and freedoms of Users and has communicated appropriate information to Users to enable them to exercise these rights and freedoms.

4.- CHANGES TO THE PRIVACY POLICY

We may update this Privacy Policy in the future. We will notify you of changes by sending a notice to the email address provided and/or by posting a notice in a prominent place on our website.

5.- CONTACT

If you have any questions about this Privacy Policy, please contact us at:

E-mail: **info@hefora.com**

Address: FISIO CONSULTORES, S.L.. Calle Magallón, - 12Cadrete50420 (ZARAGOZA).

8.- BIBLIOGRAPHY

1. Huang C, Wang Y, Li X, Ren L, Zhao J, Hu Y, et al. Clinical features of patients infected with 2019 novel coronavirus in Wuhan, China. Lancet. 2020;395(10223):497-506.
2. Sanidad Md. Enfermedad por coronavirus, COVID-19. Actualización, 12 noviembre, 2020.2020:[101 p.].
3. Cucinotta D, Vanelli M. WHO Declares COVID-19 a Pandemic. Acta Biomed. 2020;91(1):157-60.
4. SEDISA. ara la atención integral al paciente post-COVID:[28 p.].
5. Kim SY, Kumble S, Patel B, Pruski AD, Azola A, Tatini AL, et al. Managing the Rehabilitation Wave: Rehabilitation Services for COVID-19 Survivors. Arch Phys Med Rehabil. 2020.
6. Aragón. Gd. Información coronavirus COVID-19 Aragón. 2020 [Available from: https://www.aragon.es/coronavirus.
7. Cipollaro L, Giordano L, Padulo J, Oliva F, Maffulli N. Musculoskeletal symptoms in SARS-CoV-2 (COVID-19) patients. J Orthop Surg Res. 152020. p. 178.
8. Wang D, Hu B, Hu C, Zhu F, Liu X, Zhang J, et al. Clinical Characteristics of 138 Hospitalized Patients With 2019 Novel Coronavirus-Infected Pneumonia in Wuhan, China. Jama. 2020;323(11):1061-9.
9. Guan W-j, Ni Z-y, Hu Y, Liang W-h, Ou C-q, He J-x, et al. Clinical characteristics of 2019 novel coronavirus infection in China. medRxiv. 2020:2020.02.06.20020974.
10. Wu Z, McGoogan JM. Characteristics of and Important Lessons From the Coronavirus Disease 2019 (COVID-19) Outbreak in China: Summary of a Report of 72 314 Cases From the Chinese Center for Disease Control and Prevention. Jama. 2020;323(13):1239-42.
11. Laxe S, Miangolarra Page JC, Chaler J, Gil Fraguas L, Gómez A, Luna F, et al. Rehabilitation in the time of COVID-19. Rehabilitacion. 2020;54(3):149-53.
12. Bury TJ, Stokes EK. On "Musculoskeletal Physical Therapy During the COVID-19 Pandemic: Is Telerehabilitation the Answer?" Turolla A, Rossettini G, Viceconti A, Palese A, Geri T. Phys Ther. 2020;100:1260-1264. Phys Ther. 1002020. p. 1883.
13. Thomas P, Baldwin C, Bissett B, Boden I, Gosselink R, Granger CL, et al. Physiotherapy management for COVID-19 in the acute hospital setting: clinical practice recommendations. J Physiother. 2020;66(2):73-82.

14. Duan L, Zhu G. Psychological interventions for people affected by the COVID-19 epidemic. Lancet Psychiatry. 2020;7(4):300-2.
15. Gonzalez-Gerez JJ, Bernal-Utrera C, Anarte-Lazo E, Garcia-Vidal JA, Botella-Rico JM, Rodriguez-Blanco C. Therapeutic pulmonary telerehabilitation protocol for patients affected by COVID-19, confined to their homes: study protocol for a randomized controlled trial. Trials. 2020;21(1):588.
16. Vasconcello-Castillo L, Torres-Castro R, Solís-Navarro L, Rivera G, Puppo H. Evaluación Funcional y Respiratoria en Pacientes post COVID-19: ¿Cuáles son las mejores pruebas? 2020:109-15.
17. Bowden Davies KA, Pickles S, Sprung VS, Kemp GJ, Alam U, Moore DR, et al. Reduced physical activity in young and older adults: metabolic and musculoskeletal implications. Ther Adv Endocrinol Metab. 2019;10:2042018819888824.
18. de Sire A, Andrenelli E, Negrini F, Negrini S, Ceravolo MG. Systematic rapid living review on rehabilitation needs due to COVID-19: update as of April 30th, 2020. Eur J Phys Rehabil Med. 2020;56(3):354-60.
19. Ceravolo MG, de Sire A, Andrenelli E, Negrini F, Negrini S. Systematic rapid "living" review on rehabilitation needs due to COVID-19: update to March 31st, 2020. Eur J Phys Rehabil Med. 2020;56(3):347-53.
20. Curci C, Pisano F, Bonacci E, Camozzi DM, Ceravolo C, Bergonzi R, et al. Early rehabilitation in post-acute COVID-19 patients: data from an Italian COVID-19 Rehabilitation Unit and proposal of a treatment protocol. Eur J Phys Rehabil Med. 2020;56(5):633-41.
21. Richmond T, Peterson C, Cason J, Billings M, Terrell EA, Lee ACW, et al. American Telemedicine Association's Principles for Delivering Telerehabilitation Services. Int J Telerehabil. 2017;9(2):63-8.
22. Cottrell MA, Russell TG. Telehealth for musculoskeletal physiotherapy. Musculoskelet Sci Pract. 2020;48:102193.
23. Barbosa MT, Sousa CS, Morais-Almeida M, Simões MJ, Mendes P. Telemedicine in COPD: An Overview by Topics. Copd. 2020;17(5):601-17.
24. Piotrowicz E PROGOPPSGRSDIRPMKIKTMABM, Zareba W. Hybrid telerehabilitation and telecare is more efffective than usual care in improving peak vo2 and 6-minute walk distance but is not associated with reduction of 24-months mortality except in the most experienced center -result of the first randomized, p. Circulation. 2019;140.

25. Piotrowicz E PROGPMBM, Zaręba W. Hybrid comprehensive telerehabilitation in heart failure patients (TELEREH-HF): a randomized, multicenter, prospective, open-label, parallel group controlled trial-Study design and description of the intervention. American heart journal. 2019;217:148-58.
26. Thompson DA, Leimig R, Gower G, Winsett RP. Assessment of depressive symptoms during post-transplant follow-up care performed via telehealth. Telemed J E Health. 2009;15(7):700-6.
27. Agostini M, Moja L, Banzi R, Pistotti V, Tonin P, Venneri A, et al. Telerehabilitation and recovery of motor function: a systematic review and meta-analysis. Journal of telemedicine and telecare. 2015;21(4):202-13.
28. Grona SL, Bath B, Busch A, Rotter T, Trask C, Harrison E. Use of videoconferencing for physical therapy in people with musculoskeletal conditions: A systematic review. J Telemed Telecare. 2018;24(5):341-55.
29. Bhaskar S, Bradley S, Chattu VK, Adisesh A, Nurtazina A, Kyrykbayeva S, et al. Telemedicine as the New Outpatient Clinic Gone Digital: Position Paper From the Pandemic Health System REsilience PROGRAM (REPROGRAM) International Consortium (Part 2). Front Public Health. 2020;8:410.
30. Bennell KL, Marshall CJ, Dobson F, Kasza J, Lonsdale C, Hinman RS. Does a Web-Based Exercise Programming System Improve Home Exercise Adherence for People With Musculoskeletal Conditions?: A Randomized Controlled Trial. Am J Phys Med Rehabil. 2019;98(10):850-8.
31. Claes J BRWCBAGCAMMNMKMNCIWDBWFD, Cornelissen VA. PATHway I: design and rationale for the investigation of the feasibility, clinical effectiveness and cost-effectiveness of a technology-enabled cardiac rehabilitation platform. BMJ open. 2017;7(6):e016781-e.
32. Hosseiniravandi M, Kahlaee AH, Karim H, Ghamkhar L, Safdari R. Home-based telerehabilitation software systems for remote supervising: a systematic review. International journal of technology assessment in health care. 2020;36(2):113-25.
33. Daher A, Balfanz P, Cornelissen C, Müller A, Bergs I, Marx N, et al. Follow up of patients with severe coronavirus disease 2019 (COVID-19): Pulmonary and extrapulmonary disease sequelae. Respir Med. 2020;174:106197.

34. Sanidad Md. Manejo clínico del COVID-19: atención hospitalaria.2020 20/12/2020:[28 p.]. Available from: https://www.mscbs.gob.es/profesionales/saludPublica/ccayes/alertasActual/nCov/documentos/Protocolo_manejo_clinico_ah_COVID-19.pdf.
35. Lobo A, Saz P, Marcos G, Día JL, de la Cámara C, Ventura T, et al. [Revalidation and standardization of the cognition mini-exam (first Spanish version of the Mini-Mental Status Examination) in the general geriatric population]. Med Clin (Barc). 1999;112(20):767-74.
36. Aragón. CPdFd. Guía de intervención del Fisioterpeuta en la Atención hospitalaria del paciente infectado por el nuevo coronavirus (SARS-COOV-2)2020.
37. Madrid. CPdFdlCd. Recomendaciones de Fisioterapia Respiratoria y Ejercicio Terapéutico para personas confinadas en fase de recuperación por COVID-19. 2020.
38. Greenhalgh T, Javid B, Knight M, Inada-Kin M. What is the efficacy and safety of rapid exercise test for extertional desaturation in Covid-19: a rapid systematic review.: University of Oxford; 2020.
39. Learmonth YC, Dlugonski D, Pilutti LA, Sandroff BM, Klaren R, Motl RW. Psychometric properties of the Fatigue Severity Scale and the Modified Fatigue Impact Scale. J Neurol Sci. 2013;331(1-2):102-7.
40. Bernal-Vargas L, Riveros-Munévar F, Vinaccia-Alpi S, Quiceno-Sierra J-M. Estructura factorial y consistencia interna de la Escala de severidad de fatiga en población colombiana con enfermedades crónicas. Enfermería Global. 2017;16:37-49.
41. Duarte RE, Velasco E, Sánchez-Sosa JJ, Reyes-Lagunes LI. Validación psicométrica de la Escala de gravedad de la fatiga en médicos residentes mexicanos.: Educación Médica; 2019. p. 28-36.
42. Hewlett S, Dures E, Almeida C. Measures of fatigue: Bristol Rheumatoid Arthritis Fatigue Multi-Dimensional Questionnaire (BRAF MDQ), Bristol Rheumatoid Arthritis Fatigue Numerical Rating Scales (BRAF NRS) for severity, effect, and coping, Chalder Fatigue Questionnaire (CFQ), Checklist Individual Strength (CIS20R and CIS8R), Fatigue Severity Scale (FSS), Functional Assessment Chronic Illness Therapy (Fatigue) (FACIT-F), Multi-Dimensional Assessment of Fatigue (MAF), Multi-Dimensional Fatigue Inventory (MFI), Pediatric Quality Of Life (PedsQL) Multi-Dimensional Fatigue Scale, Profile of Fatigue (ProF), Short Form 36 Vitality Subscale (SF-36 VT), and Visual Analog Scales (VAS). Arthritis Care Res (Hoboken). 2011;63 Suppl 11:S263-86.

43. Stone P, Richards M, A'Hern R, Hardy J. Fatigue in patients with cancers of the breast or prostate undergoing radical radiotherapy. J Pain Symptom Manage. 2001;22(6):1007-15.
44. Schneeberger EE, Marengo MF, Dal Pra F, Maldonado Cocco JA, Citera G. Fatigue assessment and its impact in the quality of life of patients with ankylosing spondylitis. Clin Rheumatol. 2015;34(3):497-501.
45. Pennisi M, Malaguarnera G, Di Bartolo G, Lanza G, Bella R, Chisari EM, et al. Decrease in Serum Vitamin D Level of Older Patients with Fatigue. Nutrients. 2019;11(10).
46. Gimeno-Santos E, Arbillaga-Etxarri A, Vilaro J, Balañá A, Barberan-Garcia A, del Corral T, et al. Reference equations for 6-minute walk test in Spanish population2015. PA1543 p.
47. Rikli RE, Jones CJ. Development and validation of criterion-referenced clinically relevant fitness standards for maintaining physical independence in later years. Gerontologist. 2013;53(2):255-67.
48. Cobo-Mejía EA, Ochoa González ME, Y. RCL, Vargas Niño DM, Sáenz Pacheco AM, Sandoval-Cuellar C. Confiabilidad del Senior Fitness Test versión en español, para población adulta mayor en Tunja-Colombia.: Arch Med Deporte; 2016. p. 382-6.
49. Cuellar CS, Mejía EAC, Ochoa ME, Niño DMV, Castillo LYR, González LMB, et al. VALIDACIÓN DEL SENIOR FITNESS TEST EN LA CIUDAD DE TUNJA. Expomotricidad. 2015.
50. Boneth Collantes M, Ariza García CL, Angarita Fonseca A, Parra Patiño J, Monsalve A, Gómez E. Reproducibilidad de las pruebas Arm Curly Chair Stand para evaluar resistencia muscular en población adulta mayor. Revista Ciencias de la Salud. 2012;10:179-93.
51. Vaidya T, Chambellan A, de Bisschop C. Sit-to-stand tests for COPD: A literature review. Respir Med. 2017;128:70-7.
52. Houchen-Wolloff L, Daynes E, Watt A, Chaplin E, Gardiner N, Singh S. Which functional outcome measures can we use as a surrogate for exercise capacity during remote cardiopulmonary rehabilitation assessments? A rapid narrative review. ERJ Open Res. 2020;6(4).
53. Langhammer B, Stanghelle JK. The Senior Fitness Test. Journal of Physiotherapy. 2015;61(3):163.
54. McAllister LS, Palombaro KM. Modified 30-Second Sit-to-Stand Test: Reliability and Validity in Older Adults Unable to Complete Traditional Sit-to-Stand Testing. J

Geriatr Phys Ther. 2020;43(3):153-8.
55. Klok FA, Boon G, Barco S, Endres M, Geelhoed JJM, Knauss S, et al. The Post-COVID-19 Functional Status scale: a tool to measure functional status over time after COVID-19. Eur Respir J. 562020.
56. Sherer M, Maddux JE, Mercandante B, Prentice-Dunn S, Jacobs B, Rogers RW. The Self-Efficacy Scale: Construction and Validation. Psychological Reports. 1982;51(2):663-71.
57. Bosscher RJ, Smit JH. Confirmatory factor analysis of the General Self-Efficacy Scale. Behaviour Research and Therapy. 1998;36(3):339-43.
58. López-Torrecillas F, García J, Cañadas GA, Uclés IR, de la Fuente EI. Validity of Self-Efficacy Scale Scores for a Spanish Sample. Psychological Reports. 2006;98(2):437-50.
59. Herrero R, Espinoza M, Molinari G, Etchemendy E, Garcia-Palacios A, Botella C, et al. Psychometric properties of the General Self Efficacy-12 Scale in Spanish: General and clinical population samples. Comprehensive Psychiatry. 2014;55(7):1738-43.
60. Sanjuan Suarez P, Pérez García AM, Bermúdez Moreno J. Escala de Autoeficacia general:datos psicométricos de la adaptación para población española.: Psicothema; 2000. p. 509-13.
61. Antony MM, Bieling PJ, Cox BJ, Enns MW, Swinson RP. Psychometric properties of the 42-item and 21-item versions of the Depression Anxiety Stress Scales in clinical groups and a community sample. Psychological Assessment. 1998;10(2):176-81.
62. Lovibond PF, Lovibond SH. The structure of negative emotional states: comparison of the Depression Anxiety Stress Scales (DASS) with the Beck Depression and Anxiety Inventories. Behav Res Ther. 1995;33(3):335-43.
63. Daza P, Novy DM, Stanley MA, Averill P. The Depression Anxiety Stress Scale-21: Spanish Translation and Validation with a Hispanic Sample. Journal of Psychopathology and Behavioral Assessment. 2002;24(3):195-205.
64. Ruiz FJ, García Martín MB, Suárez Falcón JC, Odriozola González P. The Hierarchical Factor Structure of the Spanish Version of Depression Anxiety and Stress Scale -21. International Journal of Psychology and Psychological Therapy. 2017;17(1):97-105.
65. Moreno-Murcia JA, Marzo JC, Martinez-Galindo C, Conte Marin L. Validación de le Escala de "Satisfacción de las Necesidades Psicológicas Básicas" y del Cuestionario de la "Regulación Conductual en el Deporte" al contexto español. Revista internacional de ciencias del deporte. 2011;26:355-69.

66. da Cruz MMA, Ricci-Vitor AL, Borges GLB, da Silva PF, Turri-Silva N, Takahashi C, et al. A Randomized Controlled Crossover Trial of Virtual Reality in Maintenance Cardiovascular Rehabilitation in a Low-Resource Setting: Impact on Adherence, Motivation, and Engagement. Phys Ther. 2021.
67. Guddal MH, Stensland SO, Smastuen MC, Johnsen MB, Zwart JA, Storheim K. Physical Activity Level and Sport Participation in Relation to Musculoskeletal Pain in a Population-Based Study of Adolescents: The Young-HUNT Study. Orthop J Sports Med. 2017;5(1):2325967116685543.
68. Landeta O, Calvete E. Adaptación y validación de la Escala Multidimensional de Apoyo Social Percibido. Ansiedad y Estrés. 2002; 8(2-3): 173-182.
69. Granero M, Perman G, Vazquez Peña F, Barbaro CA, Zozaya ME, Martinez Infantino VS, et al. [Validation of the Spanish version of the Lubben Social Network Scale-6]. Rev Fac Cien Med Univ Nac Cordoba. 2020;77(4):296-300.
70. Vilagut G, Valderas JM, Ferrer M, Garin O, López-García E, Alonso J. [Interpretation of SF-36 and SF-12 questionnaires in Spain: physical and mental components]. Med Clin (Barc). 2008;130(19):726-35.
71. Vilagut G, Ferrer M, Rajmil L, Rebollo P, Permanyer-Miralda G, Quintana JM, et al. [The Spanish version of the Short Form 36 Health Survey: a decade of experience and new developments]. Gac Sanit. 2005;19(2):135-50.
72. Guerra-Tapia A, Buendía-Eisman A, Ferrando Barbera J. Final Phase in the Validation of the Cross-Cultural Adaptation of the Hair-Specific Skindex-29 Questionnaire Into Spanish: Sensitivity to Change and Correlation With the 12-Item Short-Form Health Survey. Actas Dermosifiliogr. 2019;110(10):819-29.
