# Supplementary material for: Efficacy of an asynchronous telerehabilitation program in post-COVID-19 patients: A protocol for a pilot randomized controlled trial
Source: PLoS One. 2022 Jul 19;17(7):e0270766. doi: 10.1371/journal.pone.0270766 (PMC9295945; doi:10.1371/journal.pone.0270766)
Supplement: S2 File — (PDF) [file pone.0270766.s003.pdf]

MEMORIA DE PROYECTO

Eficacia de un programa de telerehabilitación asincrónico en pacientes post-COVID-19: estudio de viabilidad.

ÍNDICE

TÍTULO 3
EQUIPO INVESTIGADOR 3
1.- ANTECEDENTES/ JUSTIFICACIÓN 4
2.- HIPÓTESIS 6
2.1.- HIPÓTESIS 7
3.- OBJETIVOS 7
3.1.- OBJETIVO GENERAL 7
3.2.- OBJETIVOS ESPECÍFICOS 7
4.- METODOLOGÍA 8
4.1.- DISEÑO 8
4.2.- SUJETOS DEL ESTUDIO 9
4.2.1.- Criterios de inclusión 10
4.2.2.- Criterios de exclusión 11
4.2.3.- Aleatorización 11
4.3.- INTERVENCIÓN 12
4.3.1.- Protocolo de intervención. 12
4.3.2.- Grupo experimental 14
4.3.3.- Grupo control 15
4.4.- VARIABLES 16
4.4.1.-Variable principal fase previa observacional: detección de necesidades 16
4.4.2.-Variable principal ensayo clínico: nivel de fatiga 17
4.4.3.-Variables secundarias 18
4.5.- TAMAÑO MUESTRAL 22
4.6.- ANÁLISIS ESTADÍSTICO 22
4.7.- SESGOS Y LIMITACIONES 23
5.- ASPECTOS ÉTICOS 23
5.1.- CONSENTIMIENTO INFORMADO 23
5.2.- RIESGOS Y CONTINGENCIAS 24
5.3.- PROTECCIÓN DE DATOS 24
5.4.- IMPLICACIONES ASISTENCIALES 28
6.- CRONOGRAMA 28
7.- ANEXOS 30
8.- BIBLIOGRAFÍA 72

TÍTULO

Eficacia de un programa de telerehabilitación asincrónico en pacientes postCOVID-19: estudio de viabilidad.

EQUIPO INVESTIGADOR

- Sandra Calvo Carrión. Fisioterapeuta. Profesora del Grado de Fisioterapia. Grupo de Investigación iPhysio. Facultad de Ciencias de la Salud. Universidad San Jorge. Zaragoza.
- Carolina Jiménez Sánchez. Fisioterapeuta. Profesora del Grado de Fisioterapia. Grupo de Investigación iPhysio. Facultad de Ciencias de la Salud. Universidad San Jorge. Zaragoza.
- Sara Pérez Palomares. Fisioterapeuta. Profesora del Grado de Fisioterapia. Grupo de Investigación iPhysio. Facultad de Ciencias de la Salud. Universidad San Jorge. Zaragoza.
- Beatriz Carpallo Porcar. Fisioterapeuta. Grupo de Investigación iPhysio. Facultad de Ciencias de la Salud. Universidad San Jorge. Zaragoza.
- Laura Romo Calvo. Fisioterapeuta del Hospital Provincial Nuestra Señora de Gracia. Zaragoza.
- Pablo Herrero Gallego. Fisioterapeuta e investigador. Profesor Ayudante Doctor Universidad de Zaragoza. Zaragoza.
- Natalia Brandín de la Cruz. Fisioterapeuta. Profesora del Grado de Fisioterapia. Grupo de Investigación iPhysio. Facultad de Ciencias de la Salud. Universidad San Jorge. Zaragoza.
- Dr. Jorge Alamillo Salas. Médico Rehabilitador Hospital Royo Villanova.
- Irene Liñares Valera. Licenciada en psicología por la universidad de Santiago de Compostela. Profesora del Grado de Fisioterapia. Facultad de Ciencias de la Salud. Universidad San Jorge. Zaragoza.
- Laura Bafaluy Franch. Licenciada en psicología por la universidad del País Vasco (San Sebastián). Profesora del Grado de Fisioterapia. Facultad de Ciencias de la Salud. Universidad San Jorge. Zaragoza

1.- ANTECEDENTES/ JUSTIFICACIÓN

El SARS-CoV-2 es el coronavirus responsable de desarrollar la enfermedad conocida como COVID-19. Su evolución puede ir desde un transcurso asintomático, hasta evolucionar rápidamente y causar un síndrome respiratorio agudo[1]. Además de síntomas a nivel respiratorio también repercute a nivel neuromuscular y cardiovascular[2, 3]. Según los datos de diciembre de 2020 del Ministerio de Sanidad Español, desde la aparición en Wuhan (China) del SARS-CoV-2 en diciembre de 2019, se han producido en España más de 46.000 muertos y más de 1.628.200 infectados[2]. Con una tasa de mortalidad de pacientes hospitalizados del 21% según la sociedad española de directivos de la salud (SEDISA)[4]. La emergencia de Salud Pública a nivel internacional fue declarada por la Organización Mundial de la Salud (OMS)[3] el 30 de enero de 2020 y la calificación de pandemia el 11 de marzo de 2020[5.]

Desde entonces España se sitúa como el segundo país europeo en número de fallecidos por millón de habitantes y más de 16.000 personas han necesitado ingreso hospitalario[2]. En Aragón se han confirmado más de 72.500 casos y más de 8.200 personas han sido hospitalizadas, de ellas, 730 en Unidades de Cuidados Intensivos (UCI), con un total de 2.280 fallecidos hasta el 6 de diciembre de 2020 según los datos del Gobierno de Aragón[6].

Los pacientes que sufren la COVID-19 suelen presentar distintos síntomas: fiebre, tos, dolor generalizado, alteraciones gastrointestinales y disnea y fatiga, estos dos últimos síntomas se dan en cerca de la mitad de estos pacientes. La prevalencia de síntomas musculoesqueléticos en pacientes con COID-19 a nivel mundial es mayor al 50% en la población mundial, siendo mayor esta prevalencia registrada en Europa[7]. En España, dentro de los casos hospitalizados, la neumonía aparece en cerca del 80% de los casos[4] lo que incrementa el porcentaje de prevalencia de disnea y fatiga en este grupo de pacientes[1, 8, 9]. Estas consecuencias propias de la enfermedad se ven agravadas por el tiempo que pasan confinados en sus domicilios o ingresados en el hospital. El tiempo estimado desde el comienzo de la enfermedad hasta la desaparición de síntomas es de 2 semanas si la enfermedad es leve y de 3 a 8 semanas de media cuando ha sido grave[10]. Por tanto, esta inactividad adicional influye negativamente en la pérdida de la condición física a nivel muscular, cardiovascular y metabólico entre otros sistemas.

Durante la fase 3 de la epidemia se fueron suspendiendo en España y el resto de Europa los tratamientos rehabilitadores hospitalarios menos urgentes(11), como así también lo recomendó la Confederación Mundial de Fisioterapia por seguridad(12). Por esta situación de emergencia sanitaria, las directrices actuales solo recomiendan realizar rehabilitación en pacientes de COVID-19 graves o con un alto riesgo de comorbilidades(13), hasta que la situación sanitaria mejore o disminuya la presión asistencial.

De ello se deriva en parte, que actualmente un gran número de pacientes presenten secuelas; cerca del 40% de las personas dadas de alta siguen teniendo síntomas(5). Es importante destacar que, en España el 61% de los nuevos ingresos por secuelas corresponde a pacientes de COVID-19 leve que requirieron hospitalización(4). Además de las secuelas físicas, la experiencia de la enfermedad y el asilamiento ha generado en los pacientes ya dados de alta, unos mayores niveles de depresión y ansiedad, así como pérdidas de memoria(4) (14). Todo este conjunto de secuelas afecta a la calidad de vida, aumentando el riesgo de comorbilidades y ralentizando la recuperación de estos pacientes(15, 16). Aspecto que puede ser muy perjudicial especialmente en la población de edad avanzada(17, 18). Con todo esto, se recomienda el poder continuar con una rehabilitación temprana en la fase post-aguda tras la fase hospitalaria que aumente los niveles de actividad física, especialmente en pacientes sin discapacidad grave que pueda continuarse además con una telerehabilitación a largo plazo(19).

La telerehabilitación, por tanto, parece ser una opción viable, *"mediante dispositivos que se pueden llevar puestos, aplicaciones de telefonía móvil, realidad virtual"* etc.(18) para disminuir las secuelas de la enfermedad y prevenir la discapacidad(17, 20). Por telerehabilitación se entiende a la realización de tratamientos de rehabilitación mediante las nuevas tecnologías de la información y comunicación (TIC)(21) y se incluye dentro del término "telesalud". Ésta, bien puede realizarse en directo o de forma asincrónica(22). Ya ha demostrado ser eficaz en otras patologías como: cardíaca, neurológica, respiratoria o musculoesquelética(23-27). Entre las ventajas de este tipo de intervención está la posibilidad de dar mayor cobertura sanitaria a personas con barreras geográficas(28), con otras comorbilidades, en situación de dependencia o que deban estar confinadas en sus casas(29).

Además, varios estudios confirman que el trabajo mediante tecnologías a distancia influye positivamente en la adherencia de los pacientes(30, 31) Gran parte de la población a la que

se le prescribe rehabilitación domiciliaria no la realiza con la frecuencia recomendada[32]. Por este motivo se hace necesario incrementar el nivel de adherencia de los pacientes con rehabilitación domiciliaria, y la telerehabilitación podría ser la respuesta, mejorando la satisfacción y con ello la adherencia de los pacientes[29].

El elemento diferenciador e innovador del presente estudio reside, por una parte, en el método de telerehabilitación: asincrónica; por otra en los pacientes: centrándonos en tratar las secuelas en los pacientes dados de alta; y por último en el tipo de programa de intervención basado en rehabilitación física: centrado en la mejora de la fatiga. No existen estudios previos que hayan valorado la eficacia de un programa asincrónico, sino que la mayoría se han centrado en el análisis de la telerehabilitación sincrónica[32] por lo que es necesario llevar a cabo estudios que permitan determinar si la telerehabilitación asincrónica también es eficaz en estos pacientes.

Actualmente los pacientes dados de alta continúan su rehabilitación con recomendaciones de salud y en algunos casos revisiones periódicas. Este proyecto permitiría ofrecer un servicio gratuito para el paciente de fácil implementación y seguimiento que a priori supondrá un beneficio para los pacientes y una alternativa viable a nivel costo/beneficio a largo plazo[29].

Según los resultados revelados tras este estudio de viabilidad, se pretende adaptar la intervención para la realización futura de un ensayo clínico que ofrezca datos extrapolables a la población y poder así, ampliar este tipo de intervención a distancia a otras patologías musculoesqueléticas dentro de los servicios de rehabilitación.

2.- HIPÓTESIS

Las secuelas post-COVID-19, tanto de la hospitalización y/o confinamiento, como de la propia enfermedad, están generando una disminución en la capacidad física de los pacientes y un aumento de la fatiga como consecuencia[33]. Dichos pacientes, dejan de recibir tratamiento rehabilitador hospitalario, por ello la telerehabilitación aparece como un complemento a corto y largo plazo, que podría ayudar a los pacientes a conseguir un estado funcional y, por tanto, una mejor calidad de vida.

2.1.- HIPÓTESIS PRINCIPAL

H_1: la implantación de un programa de 12 semanas de terapia física y educación terapéutica mediante un software de telerehabilitación asincrónica, es viable y eficaz preliminarmente en el aumento de los niveles de condición física en cuanto a fatiga, capacidad funcional, autoeficacia en el manejo del proceso y en la mejora de los factores psicosociales (calidad de vida, depresión-ansiedad-estrés, motivación, compromiso y apoyo social percibido), así como en la adherencia al tratamiento, de los pacientes que han permanecido ingresados por COVID-19 en el Hospital Provincial Nuestra Señora de Gracia (HPNSG) y el Hospital Royo Villanova (HRV) del Área 1 de Salud de Zaragoza, y que han sido dados de alta.

3.- OBJETIVOS

3.1.- OBJETIVO GENERAL

1.- Analizar la viabilidad y la eficacia preliminar en la condición física, de un programa de terapia física y educación terapéutica de 12 semanas, mediante telerehabilitación asincrónica en pacientes post-COVID-19 que vayan siendo dados de alta en el HPNSG y el HRV, y comparar sus efectos con los pacientes que han realizado este mismo programa, pero en formato no telemático.

2.- Determinar las principales secuelas físicas, así como la influencia de los factores psicosociales derivados de la COVID-19 en los pacientes que han sido dados de alta (como máximo 3 meses antes de la fecha de inicio del estudio) en el Área 1 de Salud de Zaragoza y que hubieran sido ingresados en el HPNSG y el HRV, mediante una encuesta de necesidades diseñada a tal efecto.

3.2.- OBJETIVOS ESPECÍFICOS

1.- Analizar los efectos a nivel de la severidad de la fatiga de un programa de terapia física mediante un software de telerehabilitación asincrónico en pacientes post-COVID- 19 que vayan siendo dados de alta en el HPNSG y el HRV, medida con la escala de severidad de la fatiga (FSS).

2.- Valorar el cambio en la capacidad funcional (resistencia aeróbica y fuerza evaluadas en las actividades de la vida diaria) de un programa de terapia física mediante un software de telerehabilitación asincrónico en pacientes post-COVID-19 que vayan siendo dados de alta en el HPNSG y el HRV. Midiendo el cambio en la resistencia aeróbica con el test de 6 minutos marcha, la fuerza de miembros superiores e inferiores con dos ítems del senior fitness test (FST) y la escala del estado funcional post-Covid-19 (PCFS).

3.- Analizar los cambios en los factores psicosociales: autoeficacia en el manejo del proceso, depresión ansiedad y estress, motivación, compromiso y el apoyo social percibido, tras la realización de un programa de terapia física mediante un software de telerehabilitación asincrónico en pacientes post-COVID-19 que vayan siendo dados de alta en el HPNSG y el HRV medida con la escala de autoeficacia general (GSES), la escala de depresión, ansiedad y estrés (DASS-21), el cuestionario de regulación conductual en el deporte (BRSQ-36), el registro de actividad del paciente, y la escala multidimensional de apoyo social percibido (MSPSS), respectivamente.

4.- Analizar los cambios en la calidad de vida de un programa de terapia física mediante un software de telerehabilitación asincrónico en pacientes post-COVID-19 que vayan siendo dados de alta en el HPNSG y el HRV con la escala de calidad de vida SF-12 (versión 2).

5.- Determinar el nivel de adherencia al programa de un programa de terapia física, tasas de reclutamiento y satisfacción, en pacientes post-COVID-19 que vayan siendo dados de alta en el HPNSG y el HRV con el registro de los diarios y una breve entrevista sobre la satisfacción y aceptabilidad de los procedimientos en una escala Likert de 5 puntos en ambos grupos.

4.- METODOLOGÍA

4.1.- DISEÑO

Estudio piloto y de viabilidad con diseño de ensayo clínico aleatorizado simple ciego. El estudio ha sido aprobado por parte de la dirección del HPNSG de Zaragoza y la dirección

del HRV de Zaragoza, y cuenta con la autorización del Vicerrectorado de Investigación de la Universidad San Jorge. Además, será registrado en clinicaltrials.gov.

El estudio de viabilidad contará con dos fases simultáneas pero bien diferenciadas:

- Una de las fases consistirá en la realización de una encuesta inicial de detección de necesidades derivadas de la COVID-19. La encuesta se realizará a todos los pacientes que hubieran sido dados de alta en el HPNSG y el HRV como máximo 3 meses antes desde la fecha de inicio de este estudio y que hayan dado su consentimiento informado en el momento del alta hospitalaria para participar en el estudio observacional.

- La otra fase consistirá en reclutar en el HPNSG y el HRV, a los pacientes que sean dados de alta desde la fecha de inicio del presente estudio y que cumplan criterios de elegibilidad.

  A estos pacientes (que sean dados de alta), también se les pasará la encuesta de necesidades para tener una visión general en el momento de su enrolamiento en el estudio, si deseasen participar y firmasen el consentimiento.

  El reclutamiento del ensayo clínico se hará a medida que a los pacientes se les vaya indicando que van a ser dados de alta desde la fecha de inicio del estudio, siendo informados mediante la hoja de información al paciente, y firmando el consentimiento informado si desean participar (de forma presencial antes del alta hospitalaria).

Además, se realizará durante el tiempo que dure el estudio un control telefónico cada dos semanas desde la fecha de comienzo del programa de intervención. Así mismo, se estiman un seguimiento post-intervención a los 3 y 6 meses.

4.2.- SUJETOS DEL ESTUDIO

Se considerarán sujetos del estudio observacional a todos los pacientes que hubieran sido dados de alta en el HPNSG y el HRV como máximo 3 meses antes desde la fecha de inicio de este estudio. Dichos pacientes simplemente serán encuestados mediante una encuesta de necesidades creada específicamente para este estudio.

Se considerarán sujetos del ensayo longitudinal a pacientes del HPNSG y el HRV de Zaragoza, ingresados por COVID-19 y que vayan a ser dados de alta en el momento de su inclusión en el estudio.

Los datos de la historia clínica previa serán proporcionados por el HPNSG y el HRV. El reclutamiento para el ensayo longitudinal será realizado en el HPNSG y el HRV, y la encuesta de necesidades del estudio observacional, se realizarán in situ desde el HPNSG, y serán realizadas por parte del personal sanitario del HPNSG del servicio de fisioterapia del Hospital, con la autorización de la Dirección del Hospital quiénes informarán presencialmente mediante la hoja de información a los posibles participantes, y pedirán a los pacientes que cumplan los criterios de inclusión y que decidan participar que den su consentimiento y lo firmen. Posteriormente se les dará cita, para realizarles la evaluación pre-intervención (se detalla más abajo).

4.2.1.- Criterios de inclusión

- Edad entre 18 y 75 años*.
- Pacientes que hayan estado hospitalizados más de 5 días por COVID-19.
- Pacientes que hayan sido dados de alta en el HPNSG y el HRV y presenten un grado de fatiga mayor de 4 puntos de media en la FSS**.
- Deambulación independiente, aunque sea mediante el uso cualquier ayuda técnica.
- Haber firmado el consentimiento informado.

*El límite superior de edad determinada en 75 años pretende evitar un sesgo en la intervención por tratarse de un método mediante aplicaciones tecnológicas que requieren acceso y conocimientos de uso básico de internet.
Por otra parte, el paciente medio en España hospitalizado por COVID-19 presenta una edad de 69 años con algún factor de riesgo (hipertensión 51%; dislipemia 39,7%; diabetes 19,4%).

**Una puntuación menor de 4 puntos en la FSS se considera baja fatiga. El programa se centra en intervenir sobre aquellos pacientes que presenten fatiga moderada o alta entendiendo que son quienes presentan mayores necesidades de rehabilitación.

**Se valorarán las condiciones físicas de los pacientes que han estado tanto en UCI/planta como solo en planta para estratificar en función de su situación en el momento del alta hospitalaria en relación a la severidad de la fatiga.

4.2.2.- Criterios de exclusión

- Pacientes con enfermedades neurológicas graves a nivel central y/o periférico que les impidan seguir el programa*.
- Pacientes con insuficiencia respiratoria grave: SaO_2 <90% o frecuencia respiratoria ≧30[34]*.
- Pacientes con patología reumática o lesión musculoesquelética aguda en su historia clínica*.
- Pacientes con comorbilidades cardíacas inestables, como arritmias, alta presión arterial, angina de pecho u otras patologías que tengan contraindicado el entrenamientos de intensidad moderada*.
- Pacientes sin acceso a internet móvil u ordenador con internet en el domicilio**.
- Pacientes que obtengan una puntuación ≤24 en el Mini-Examen Cognoscitivo (MEC)[35] (ANEXO I)**
- Que no sean capaces de seguir las instrucciones orales y escritas en el idioma español**.

*Se excluyen aquellos pacientes cuya situación médica impida la realización de este programa de actividad física sin seguimiento en directo, de forma clínicamente segura, debido a que la herramienta empleada no tiene un seguimiento *in vivo* durante la realización de los ejercicios.
** Por otra parte, se pretende excluir aquellas personas que no puedan seguir el tratamiento, bien por falta de comprensión cognitiva o de idioma, o por accesibilidad evitando sesgos en la intervención.

4.2.3.- Aleatorización

Los pacientes detectados para el ensayo longitudinal que deseen participar, cumplan los criterios de inclusión y firmen el consentimiento informado pasarán a ser enrolados en el estudio.

El ensayo clínico de viabilidad presentará dos grupos, un grupo experimental que recibirá el programa de telerehabilitación en formato de telerehabilitación y un grupo control que

lo recibirá en formato papel.

La aleatorización se realizará mediante el software *www.randomizer.org* otorgando a cada sujeto un código identificatorio (CI) garantizando su anonimato. La persona responsable del proyecto conocerá la lista de casos con el CI permaneciendo cegada al paciente. El evaluador permanecerá cegado al número identificatorio y a la asignación. Una vez terminado el estudio se abrirá el sobre cerrado con la lista de casos y CI. Se estratificará en tres niveles iniciales de fatiga según la escala de severidad de la fatiga (FSS) y se crearán grupos homogéneos con control de las posibles variables de confusión como la toma de medicación o haber recibido tratamiento de fisioterapia anterior o actualmente.

4.3.- INTERVENCIÓN

4.3.1.- Protocolo de intervención.

Se diseñará un programa de rehabilitación física de tres niveles de intensidad, cada uno compuesto por seis ejercicios, siguiendo las recomendaciones los Colegios de Fisioterapia de las Comunidades Autónomas de Andalucía, Madrid y Aragón y las recomendaciones del área de fisioterapia de la Sociedad Española de Neumología y Cirugía Torácica SEPAR[36, 37]. El primer nivel incluirá aquellos que obtengan una puntuación mayor o igual a 4 y menor de 5 (4 a 4,9); el segundo nivel estará formado por aquellos que obtengan una puntuación mayor o igual a 5 puntos de media y menor a 6 (5 a 5,9). El tercer nivel estará formado por los pacientes con más fatiga, con puntuaciones entre 6 y 7 (6 a 7) de media en la FSS.

El programa de ejercicios se dividirá en una parte de ejercicio aeróbico (1 ejercicio), otra de entrenamiento de fuerza (3 ejercicios) y una de ejercicios respiratorios (2 ejercicios) durante 12 semanas, tres días a la semana (ANEXO II).

Del mismo modo, después de la encuesta de necesidades, se creará una lista de recomendaciones para el automanejo de las secuelas de la enfermedad. Tanto, del programa de ejercicios como de educación terapéutica se crearán vídeos explicativos para el grupo intervención y un folleto para el grupo control. Todos los pacientes dispondrán del mismo programa de rehabilitación física y educativa, diferenciándose cada grupo de

asignación en el formato de la prescripción.
El diseño de este programa será realizado por los investigadores: B. Carpallo, C. Jiménez, S. Calvo, S. Pérez y P. Herrero.
A los pacientes que hayan sido enrolados tras cumplir los criterios de inclusión y que hayan firmado el consentimiento informado (presencialmente) se les citará para ser valorados mediante una evaluación pre-intervención con la batería de test que se comentan en el siguiente apartado.

Al finalizar dicha valoración un miembro del equipo investigador explicará a cada paciente la batería de ejercicios prescritos según su nivel inicial de fatiga, dependiendo del grupo al que hayan sido asignados, (dispondrá de una lista con el orden de citación y el IC, desconociendo el nombre del paciente). Al finalizar se comprobará la comprensión de la intervención pidiendo que expliquen los ejercicios. Al grupo control se le entregará en este momento el folleto con los ejercicios y al grupo experimental se le instalará y explicará la aplicación Health for All (HEFORA), una plataforma diseñada para prescribir ejercicio terapéutico por profesionales formados y que actualmente está disponible desde el Colegio Oficial de Fisioterapeutas de Aragón para todos sus fisioterapeutas colegiados de forma gratuita. La plataforma registra de manera encriptada y anonimizada los datos personales (nombre y apellidos) accesos de cada usuario a la plataforma y las veces que ha visualizado los vídeos. Estos datos de acceso y visualización se facilitarán únicamente a los investigadores principales del estudio piloto con el objetivo de registrar la adherencia y compromiso al programa de telerehabilitación. El paciente en su primer acceso y registro en la plataforma deberá de aceptar la política de protección de datos y privacidad de la plataforma (ANEXO XVIII). Además se aporta en la Hoja de Información al paciente, la política completa de protección de datos de la plataforma.

Pasadas las 12 semanas en cada paciente será citado de nuevo tras la última sesión de intervención, en el hospital para la valoración post-intervención con las mismas escalas de la valoración inicial. Se valorará también la satisfacción con el programa asignado con la escala Likert.
La responsable de las evaluaciones pre/post-intervención es: L. Romo, fisioterapeuta del HPNSG. La responsable de la explicación de ejercicios, entrega de folletos e instalación de la aplicación y explicación de su uso será: B. Carpallo.

Durante las 12 semanas de intervención, se realizará un control cada dos semanas a todos los pacientes. Serán llamados telefónicamente y se valorará la fatiga durante las sesiones mediante la escala de esfuerzo de Borg. Si está por debajo de 5/10 se les modificará la pauta de ejercicios del siguiente nivel para seguir las recomendaciones de intensidad [(36, 37).] Se valorará también la presencia de obstáculos en el seguimiento.
Los miembros responsables del control cada dos semanas son los investigadores: S. Calvo; C. Jiménez y B. Carpallo.

A los 3 y 6 meses de finalizar la intervención se les pasará telefónicamente a todos los pacientes la encuesta inicial de necesidades para valorar el estado general de los pacientes en relación a sus actividades de la vida diaria y fatiga. Para el seguimiento de los factores psicosociales se les pasará le encuesta general de autoeficacia abreviada (GSES), la escala de estrés y ansiedad (DASS-21) y la escala de calidad de vida (SF- 12 versión 2).

Durante todo el proceso existirá un control metodológico por parte de S. Calvo y C. Jiménez, y en concreto de las variables psicosociales por parte de las psicólogas del equipo I. Liñares y L. Bafaluy.

4.3.2.- Grupo experimental

El grupo experimental recibirá un programa de ejercicio terapéutico y educativo en forma de texto y vídeos a través de la aplicación HEFORA, de tal forma que puedan volver a visualizar los vídeos tantas veces necesiten.

La plataforma HEFORA no tendrá ningún coste económico para ninguno de los participantes del estudio. La plataforma como se ha comentado anteriormente registrará de manera encriptada y anonimizada los accesos de cada usuario a la plataforma y las veces que ha visualizado los vídeos.

Tras la valoración presencial pre-intervención, estos pacientes pasarán con el investigador responsable de la explicación del programa. A este grupo se les explicarán los tres niveles de actividad física y se les instalará la aplicación en el teléfono móvil. Del mismo modo se les explicará su uso.

Cada paciente tendrá acceso desde su cuenta particular de forma gratuita a la plataforma, con el programa completo de ejercicios y recomendaciones.

Según su nivel inicial de fatiga se le prescribirán los vídeos correspondientes a realizar para facilitar su visualización y ejecución. Cada vídeo aparecerá dentro de la “biblioteca” de la aplicación. Los vídeos serán diseñados y grabados específicamente para este fin. Cada vídeo muestra a una personada entrenada realizando paso a paso el ejercicio. Además, en la parte superior aparecerán las indicaciones fundamentales para poder realizar el ejercicio correctamente.

Cada dos semanas los pacientes recibirán la llamada de control para valorar su nivel de fatiga, y si ésta ha mejorado, se le cerrarán los vídeos de ese nivel y se le activarán los del siguiente nivel. La plataforma, además permite enviar mensajes de control para individualizar más el tratamiento domiciliario.

Además de los ejercicios, cada paciente tendrá a su disposición vídeos con recomendaciones educativas de salud que deberá ir viendo a lo largo de la intervención. Cada visualización queda registrada y podrá ser controlada únicamente por el grupo de investigación al final de la misma. Los pacientes en todo momento sabrán que queda registrado cada acceso a la plataforma. Los participandes del grupo experimental, dispondrán también de un apartado de anotaciones, donde semanalmente los participantes deberán registrar en esta plataforma si han realizado el programa de ejercicios y si han realizado actividad física adicional.

Cada dos semanas se les harán las llamadas de control mencionadas anteriormente, en las que se les preguntará por el transcurso del tratamiento, si presentan alguna dificultad en la puesta en práctica del programa y su nivel de fatiga tras la realización del mismo. Según disminuya su fatiga durante la realización de los ejercicios se les modificará la pauta de ejercicios, pasando a los del siguiente nivel.

4.3.3.- Grupo control

El grupo control recibirá tras la valoración inicial con el fisioterapeuta del HPNSG, un folleto en formato papel en el que figurarán imágenes claras y descripciones de los tres niveles de intensidad de los ejercicios. Además, aparecerán recomendaciones de educación terapéutica. El investigador encargado de la entrega de material se cerciorará de la comprensión de los ejercicios y les marcará en nivel inicial de comienzo de la

actividad, según su nivel de fatiga inicial.

En este mismo momento se les explicará el procedimiento de llamadas de control cada dos semanas, que será el mismo que para el grupo experimental. Según disminuya su fatiga durante la realización de los ejercicios se les modificará la pauta de ejercicios, pasando a los del siguiente nivel.

También se les hará entrega de un diario en el que deben registrar cada semana si han realizado los ejercicios propuestos (en carga y repeticiones y sin han realizado actividad física adicional a sus actividades de la vida diaria como caminar o hacer deporte más de 30 minutos ese día).

4.4.- VARIABLES

4.4.1.- Variable principal fase previa observacional: detección de necesidades.

***Encuesta de detección de necesidades postCOVID-19*:** cuyo objetivo es determinar las principales necesidades físicas, así como los factores psicosociales derivados de la COVID-19.
Para la detección de necesidades en los pacientes que han pasado la COVID-19 se ha diseñado una encuesta que pueda ser realizada presencialmente o telefónicamente (existiendo consentimiento informado previo) basada en otras escalas que permita ajustar el programa de rehabilitación física y las recomendaciones educativas de la fase posterior del ensayo clínico (ANEXO III).

Se compone de cuatro secciones: datos personales, hábitos de vida, características del ingreso hospitalario y consecuencias de la COVID-19. Este último apartado, específico para la detección de necesidades se compone de cuatro partes:

1.- Factores psicosociales a partir de sufrir la COVID-19 con preguntas dicotómicas sobre la presencia de ansiedad, depresión, astenia, estrés, pérdida de memoria o cefaleas.

2.- Estado funcional: basado en la escala del estado funcional post-COVID-19 (PCFS). Esta escala, diseñada específicamente para detectar el estado funcional de los pacientes que han pasado la COVID-19 [20, 38], clasifica a los pacientes en 4 niveles de

estado funcional, desde 0= sin limitación hasta 4=limitación severa.

3.- Nivel de fatiga y su interacción con la vida diaria: basado en la escala severidad de la fatiga (FSS) [39, 40] . (comentada en profundidad en el apartado 4.4.2)

4.- Otros síntomas: sobre pérdida de fuerza, dolor y otros síntomas que afecten al funcionamiento en las actividades de la vida diaria (AVD).

4.4.2.-Variable principal ensayo clínico: nivel de fatiga

La fatiga puede entenderse desde varios aspectos, no solo físico. En este estudio se pretende intervenir en capacidad funcional de los pacientes, para lo cual es imprescindible actuar sobre el nivel inicial de fatiga. Actualmente no hay escalas validadas para pacientes post-COVID-19.

*Esta variable se medirá durante la valoración pre y post intervención. Servirá de referencia para agrupar a los participantes en niveles de fatiga y prescribirles el programa que se ajuste en intensidad a sus necesidades físicas.

- Escala de Severidad de la Fatiga (FSS)[39], validada al español por Bernal-Vargas et al. y Duarte et al.[40, 41]. Fue diseñada inicialmente en 1989 por Krupp et al. para enfermedades neurológicas como lupus eritematoso y esclerosis múltiple. Progresivamente se ha ido validando en distintos idiomas, entre ellos el español y en otros grupos de población, como en Parkinson, cáncer, dolor crónico, hepatitis, espondilitis anquilosante, en población anciana, en algún sector profesional concreto, etc.[42-45]. La FSS se ajusta a nuestro propósito al relacionar el nivel de fatiga con la capacidad funcional, además se emplea para medir la fatiga derivada de patología o envejecimiento como es este caso (con una población esperada anciana). Tiene además la ventaja de ser breve, lo que facilita su aplicación y puede realizarse telefónicamente o de forma presencial.

La FSS es una escala auto-reportada compuesta por 9 ítems que relacionan la severidad de fatiga con las actividades diarias. Cada ítem se valora del 1 al 7 desde 1=totalmente en desacuerdo hasta 7= totalmente de acuerdo (ANEXO IV). Una media inferior a 3 puntos

se considera ausencia de fatiga[44], menor de 4 se considera fatiga leve, más de 4 se considera fatiga severa. Este rango puede dividirse en tres niveles de fatiga: 4=fatiga severa límite; 5= fatiga moderada; 6-7=fatiga severa alta. La versión original solo marca el corte en 4 puntos: presencia o no de fatiga.

4.4.3.-Variables secundarias

La selección de estos test se ha realizado según la bibliografía actual referente a tratamientos de rehabilitación en pacientes COVID-19, buscando la mayor homogeneidad de pruebas y recomendaciones[16].

*4.4.3.1-**Capacidad funcional**:* medida con la prueba de 6 minutos marcha, el test de 30-segundos sentarse y levantarse, el test de 30-segundos flexión de codo y la escala del estado funcional post-COVID-19.

- Prueba de marcha de 6 minutos (6MWT). Validada al castellano por Gimeno-Santos et al.[46] Los participantes en el estudio deberán recorrer la máxima distancia posible caminando durante 6 minutos. Durante el recorrido los pacientes pueden descansar si lo necesitan. Serán monitorizados con un pulsioxímetro y seguirán las indicaciones de la fisioterapeuta. La distancia se medirá en metros (ANEXO V).

- Test de 30-segundos sentarse y levantarse (STST): forma parte del Senior Fitness Test (SFT) diseñado y validado por Rikli y Jones[47] y en español por Cuellar et al. y Cobo-Mejia et al[48, 49] . Actualmente se usa este ítem como prueba aislada junto con el test de flexión de codo[50], especialmente para valorar la debilidad de los pacientes, de edad avanzada, respiratorios y que han pasado la COVID-19[16, 38, 51-53]. Otro criterio para la elección de este test de 30 segundos, ha sido la edad prevista de los participantes y su baja condición física y su validación con respecto al test de 1 minuto el pacientes de edad avanzada[54]

Esta prueba debe realizarse con una silla de altura suficiente para que los pies apoyen en el suelo. Los brazos del paciente deben estar cruzados en el pecho. Una vez en esta posición deberán levantarse y volver a la posición inicial tantas veces sea posible en 30 segundos (ANEXO VI).

- **Test de flexión de codo (ACT)**[47, 53]**:** forma parte también del SFT, mencionado en el test anterior, aunque también se emplea como prueba aislada para valorar la fuerza en pacientes desacondicionados o en población anciana. Desde la posición de sentado, deben hacerse tantos movimientos de flexo-extensión de codo cómo se pueda en 30 segundos, con el brazo dominante, con una pesa de 2kg (ANEXO VII).

- **Escala del estado funcional Post-COVID** (PCFS)[55]: escala diseñada especialmente para valorar la capacidad funcional de los pacientes que han pasado la COVID-19 en el momento de ser dados de alta, a las 4 semanas, a las 8 semanas y a los 6 meses. Actualmente no es una escala validada por la aparición reciente de la enfermedad, pero está en proceso de traducción y validación[16]. Por la escasez de otras herramientas propias y dado que completamos la valoración con otras herramientas validadas, entendemos que es oportuno adjuntar esta escala de valoración traducida al español, para la obtención de información adicional. Esta escala puede pasarse telefónicamente o ser autoadministrada. Se compone de 4 niveles de incapacidad post-COVID-19. F=fallecimiento; 0=sin limitación; 1=limitación funcional no significativa; 2=limitación funcional leve; 3=limitación funcional moderada; 4=limitación funcional severa (ANEXO VIII).

*4.4.3.2.-**Factores psicosociales**:* Autoeficacia en el manejo del proceso, depresión y ansiedad, medidas con la escala de autoeficacia general GSES y DASS-21 respectivamente.

- **Escala de autoeficacia general (GSES-12):** es una escala validada y abreviada que deriva de la original compuesta por 30 ítems y dos subescalas [56]. La versión abreviada fue desarrollada por Bosscher et al. para personas de edad avanzada[57], motivo por el que encaja en el perfil de los pacientes estimados para este estudio. Validada al español por López-Torrecillas, Herrero et al, y Sanjuán et al. [58-60] compuesta por 10 ítems, se valora en una escala de Likert siendo 1=nunca me ocurre hasta 5= siempre me ocurre. Los ítems se dividen en 3 bloques: iniciativa, esfuerzo y perseverancia (ANEXO IX).

- **Escala de depresión y ansiedad (DASS-21)[61]**: es una versión abreviada de la escala DASS-42 diseñada en 1995 por Lovibond et al[62]. Validada el español por Daza et al. y Ruiz et al. [63, 64]. Está compuesta por tres bloques de contenidos (depresión, ansiedad y estrés) valorados mediante una escala de Likert de 0=nunca me ha pasado a 3= muchas

veces o casi siempre me ha pasado (ANEXO X).

- **Cuestionario de regulación conductual en el deporte (BRSQ-36):** mide la motivación extrínseca a través de diferentes factores según el paciente realice la actividad física por creer que forma parte de un estilo de vida saludable (regulación integrada), por creer que la actividad es importante pero no placentera (regulación identificada), por sentimientos de culpabilidad (regulación introyectada) y/o por presiones externas (regulación externa). La motivación intrínseca se mide por medio de tres factores, el conocimiento (el placer de conocer más acerca de la actividad física), la ejecución (el disfrute por alcanzar metas) y la estimulación (entusiasmo que siente al estar implicado en la actividad). Otro de los factores que mide es la desmotivación, acción caracterizada por la falta de interés hacia la práctica y los sentimientos de frustración. Encontramos que esta escala está validada al contexto español[(65)]. Sus ítems son valorados mediante una escala Likert que va desde el 1 (*nada verdadero*) al 7 (*muy verdadero*) (ANEXO XI)

- **Compromiso[(66, 67)]:** será medido mediante la participación. Esta medición por tanto será realizada mediante la adherencia al programa que es otra variable de este proyecto y que se indica más abajo, la cual será establecida mediante un registro diario de la actividad que el paciente completará. (Se corresponderá con el anexo descrito para la variable adherencia).

- **Escala multidimensional de apoyo social percibido (MSPSS)[(68)]:** validada en población española por Landeta y Calvete, obteniendo altas propiedades psicométricas. Se considera una escala prioritaria para ser aplicada en personas que se encuentran en proceso de recuperación. Mide el apoyo social en tres dimensiones (familia, amigos y otros significativos) y aporta también una puntuación final. La versión utilizada consta de 12 ítems que se responden mediante una escala Likert que va desde el 1 (*totalmente en desacuerdo*) al 7 (*totalmente de acuerdo*) (ANEXO XII)

*4.4.3.3.-**Calidad de vida*** medida con el SF-12 (versión 2)

Cuestionario de calidad de vida auto-reportada (SF-12), versión 2 [(70)]. Para su realización los pacientes deberán rellenar el cuestionario autoadministrado que consta de

12 preguntas sobre la salud autopercibida. La versión 2 es una versión resumida del SF-36 realizada en el año 2002, que ha sido validada el español por Vilagut et al. y Guerra-Tapia et al.[71, 72]. Consta de 12 preguntas que recogen 8 dimensiones (función física, función social, rol físico, rol emocional, salud mental, vitalidad, dolor corporal y salud general) con varias opciones de respuesta. La puntuación final oscila entre el 0 y el 100, para cada una de las dimensiones, siendo 100 la peor valoración posible (ANEXO XIII).

*4.4.3.4.-**Adherencia*** medida con diarios y anotaciones en la plataforma de telerehabilitación.

- Adherencia: diarios y registro "anotaciones" de la plataforma: se codificará: 1=si han realizado la actividad, 0=si ese día no han hecho lo prescrito, 2= si ese día han realizado más actividad adicional. Entenderemos como actividad física adicional aquella actividad física voluntaria que exceda la actividad diaria habitual como: caminar ligero más de media hora; deporte durante más de media hora u otros ejercicios voluntarios adicionales (ANEXO XIV).

*4.4.3.5.-**Viabilidad del estudio***

- Viabilidad del estudio: se calculará mediante la tasa de reclutamiento, además del índice de adherencia indicado anteriormente, el reporte de satisfacción con el programa de ejercicios y la aplicación valorado al final del estudio, e incidencias valoradas durante las llamadas de seguimiento. Se registrará en una escala Likert 0=muy insatisfecho a 4=muy satisfecho. Las incidencias se registrarán como: 0=ningún problema; 1= algún problema solucionado; 2= problema que imposibilita el seguimiento ese período (ANEXO XV)

*4.4.3.6.-**Nivel fatiga percibida durante la intervención***

*Variable medida durante los controles cada dos semanas para modificar el nivel intensidad del programa.

- Escala de esfuerzo de Borg[31]: escala subjetiva de esfuerzo que cuantifica de 0=muy fácil a 10= muy duro el nivel de esfuerzo percibido al realizar una actividad.

Con esta herramienta se valorará el nivel de esfuerzo de cada paciente para ajustar el nivel de los ejercicios propuestos. También se empleará para que cada paciente controle la

intensidad recomendada durante el tratamiento.

En la tabla siguiente se muestra el cronograma de medición de cada una de las variables dentro del esquema general de periodización del proyecto:

	T1. Fase Previa	T2. Fase 1. Observacional	T3. Fase 2 Pre-Intervención	T4. Fase 3 Intervención Día 1	T5. Día 15	T6. Día 29	T7. Día 43	T8. Día 57	T9. Día 71	T10. Día 85	T 11. Fase 4. Post-Intervención	T 12. Fase 5. Seguimiento 1	T 12. Fase 5. Seguimiento 2	T 13. Fase 6.
**Diseño estudio**	X													
**Diseño del programa de ejericcios y educativo**	X													
**Estudio observacional**		X												
Encuesta de necesidades		X												
**Reclutamiento/Selección**		X												
**Aleatorización**			X											
**Evaluación pre/post-intervención**				X							X	X	X	
Severidad Fatiga-FSS				X							X	X	X	
Estado Funcional Post-Covid-PCFS				X							X	X	X	
Capacidad aeróbica submáxima-6MWT				X							X			
Capacidad física mmii-STST				X							X			
Capacidad física mmss-ACT				X							X			
Autoeficacia-GSES-12				X							X	X	X	
Depresión-ansiedad-estrés-DASS-21				X							X	X	X	
Apoyo social-MSPSS				X							X	X	X	
Calidad de vida-SF-12				X							X	X	X	
Motivación-BRSQ-36				X							X	X	X	
**Intervención**					X	X	X	X	X	X				
Adherencia/compromiso-Dirarios de actividad					X	X	X	X	X	X				
Fatiga-BORG					X	X	X	X	X	X				
Incidencias Likert					X	X	X	X	X	X				
Satisfacción Likert										X				
**Seguimiento**												X	X	
Análisis de datos														X
Difusión de resultados														X

4.5.- TAMAÑO MUESTRAL

El tamaño muestral será de 32 pacientes, 16 en cada grupo. Cálculo realizado con el programa G*Power 3.1 (Universidad Heinrich-Heine de Düsseldorf, Alemania), basado en un tamaño del efecto f de 0,25, un nivel α de 0,05, un nivel β del 20% y una potencia del 80%, más una tasa de abandono estimada del 30%.

4.6.- ANÁLISIS ESTADÍSTICO

El análisis estadístico se realizará con el software IBM-SPSS Statistics versión 25. La prueba de Kolmogorov-Smirnov será utilizada para determinar la normalidad de los datos. Las variables intra-grupo se medirán con el test T-Student para muestras relacionadas y U-Mann Whitney para las no paramétricas. Se realizarán análisis entre grupos y dentro de los grupos de intervención utilizando un modelo mixto de análisis de varianza (ANOVA) para

medidas repetidas con comparaciones post hoc por pares de Bonferroni cuando se detecte una distribución normal. Se realizará un análisis no paramétrico cuando se asuma una distribución no normal, utilizando la prueba U de Mann–Whitney para las comparaciones entre grupos y la prueba de Friedman con la prueba de Tukey para destacar las diferencias dentro del grupo. Las variables dicotómicas se analizarán mediante la prueba de chi-cuadrado. Se asumirá un nivel de significación del 95% ($p \leq 0,05$). Se utilizarán además estadísticos descriptivos para informar acerca de la viabilidad del reclutamiento y adherencia al programa. Las variables serán descritas en media y desviación típica (SD) o mediana y rango intercuartílico. Si hay más de un 15% de abandonos se hará un análisis por intención de tratar (ITT). El tamaño del efecto será calculado mediante la d de Cohen para determinar la significación clínica: las diferencias insignificantes, pequeñas, medianas y grandes se reflejarán en tamaños de efecto de <0,2, 0,2–0,5, 0,5–0,8 y >0,8, respectivamente.

4.7.- SESGOS Y LIMITACIONES

El presente estudio de viabilidad cuenta con el riesgo del sesgo de memoria en los participantes que no rellenen los diarios cada jornada. Para evitar que tomen las notas al final del estudio y no sean correctas se ha planteado el control cada dos semanas en el que se les recordará personalmente si han registrado la actividad realizada.

Para evitar un sesgo en la valoración de la intervención la fisioterapeuta encargada no conocerá la asignación de cada sujeto.

Con el objetivo de disminuir el sesgo de análisis en los resultados no serán repuestos aquellos casos que abandonen el estudio por problemas con la plataforma, falta de motivación a la hora de realizar el programa prescrito o cualquier otra incidencia, siempre y cuando realicen la valoración post-intervención, ya que lo que se pretende es conocer las limitaciones para la adherencia con el objetivo de proponer mejoras en un ensayo clínico aleatorizado con mayor tamaño muestral.

El seguimiento se realizará en ambos grupos, esto puede afectar al incremento de la adherencia en ambos grupos.

5.- ASPECTOS ÉTICOS

5.1.- CONSENTIMIENTO INFORMADO

Todos los pacientes firmarán un consentimiento informado el primer día que se les haga la valoración inicial. Su participación será libre y voluntaria. Ningún participante recibirá compensación económica ni de ninguna otra índole por su participación.

5.2.- RIESGOS Y CONTINGENCIAS

La intervención prevista no presenta riesgos para la salud de ninguno de los participantes, ya que se han tratado de ajustar los criterios de exclusión y evitar así la participación de pacientes en los cuáles una intervención intensiva pudiese estar contraindicada. Todos los pacientes dispondrán del teléfono del investigador del centro hospitalario de referencia ante cualquier incidencia y del investigador principal.

Además, el grupo experimental, contará con un canal de comunicación a través de la plataforma. Como fisioterapeutas colegiados, todos los investigadores cuentan con un seguro de responsabilidad civil.

5.3.- PROTECCIÓN DE DATOS

Se cumplirá en todo momento la Ley Orgánica de Protección de Datos. Se utilizarán datos codificados de forma que no se incluyan datos del sujeto en la base de datos del estudio y nadie salvo el equipo investigador pueda acceder a la identidad del participante.

Toda la política de privacidad de la plataforma HEFORA se puede consultar en https://es.hefora.com/privacy-and-cookie-policies y https://hefora.com/wp-content/uploads/2018/08/Politica_de_PRIVACIDAD.pdf. FISIO CONSULTORES S.L. es el responsable del tratamiento de los datos personales de los usuarios de la plataforma siendo los datos tratados de acuerdo con el Reglamento (UE) 2016/679 del Parlamento Europeo y del Consejo de 27 de abril de 2016 de Protección de Datos (RGPD). Como usuario tiene derecho a acceder, rectificar y suprimir los datos, así como otros derechos

indicados en la información adicional a través de dirección de correo electrónico info@hefora.com y dpo@fisioconsultores.com. (ANEXO XVIII).

La plataforma HEFORA en primer lugar recoge unos datos iniciales para crearse la cuenta como usuario, que son un nombre de usuario, una contraseña, un email y el nombre y apellidos. Una vez que el paciente tiene su cuenta creada, contacta con el investigador aceptando de forma digital el documento de autorización para tratamiento de datos personales en el estudio y una vez establecido ese vínculo el investigador puede incluir en la plataforma los datos que recoja él directamente o acceder a los resultados de los cuestionarios autoadministrados por el paciente (datos descritos en el proyecto de investigación). La fecha y hora de firma queda registrada y puede ser consultada tanto por el paciente como por el investigador.

Los datos que posteriormente a esta aceptación pueden llegarse a recoger en la plataforma si el usuario decide rellenarlos serán, en función del usuario (investigador/profesional o paciente):

- Profesionales: DNI/NIF, Nombre y apellidos, dirección postal o electrónica, teléfono, imagen, firma manual, características personales, circunstancias sociales, académicos y profesionales, detalles de empleo, información comercial, económicos, financieros y de seguro, transacciones de bienes y servicios.

- Usuarios: DNI/NIF, Nombre y apellidos, dirección postal o electrónica, teléfono, imagen, firma manual, características personales (personalidad o comportamiento), características personales, circunstancias sociales, académicos y profesionales, detalles de empleo, información comercial, económicos, financieros y de seguro, transacciones de bienes y servicios.

- Pacientes: DNI/NIF, Nombre y apellidos, dirección postal o electrónica, teléfono, imagen, firma manual, marcas físicas, salud, características personales (personalidad o comportamiento), características personales, circunstancias sociales, académicos y profesionales, detalles de empleo, información comercial, económicos, financieros y de seguro, transacciones de bienes y servicios.

2.- Sobre los datos que conserva, mientras que dure la investigación, los datos conservados serán los indicados anteriormente. Si finaliza el servicio se suprimen salvo los que haya que mantener por obligación legal. Y para un uso ulterior, investigación, estos datos se encuentran anonimizados y por tanto no son personales. El usuario tiene la posibilidad de eliminar su cuenta también una vez finalice el estudio.

3.- Sobre la Política de privacidad es la que consta en la web hefora.net y sólo los investigadores acceden a los datos, con la excepción de nombre y apellidos y email a los que puede acceder la empresa propietaria de Hefora, que no cede estos datos a terceros.

4. Respecto a las medidas de seguridad, la plataforma cumple con la GDPR, y tiene las siguientes medidas de seguridad implementadas:

- A nivel de servidor, el servidor que administra hefora.net está alojado en un centro de datos privado con tecnología en la nube sobre OpenStack. El proveedor no tiene acceso al sistema del servidor ni a sus datos. Las instantáneas realizadas se almacenan en otro centro de datos de las mismas instalaciones y su transferencia se realiza a través de una red interna aislada. La administración del servidor se realiza bajo el servicio OpenSSH con autenticación por contraseña o clave privada RSA. La comunicación entre el usuario y el servicio web se realiza bajo SSL con un certificado EV (SectigoRSA Extended Validation Secure Server CA). La comunicación entre los servicios web y la base de datos no está expuesta al exterior y se reduce a su red interna aislada administrada por los servicios del sistema Debian 9.9. Las copias de seguridad de la información se cifran a nivel del sistema bajoAES-256 y se transfieren con OpenSSH a través de la red interna bajo VLAN a otro servidor de copia de seguridad. Al igual que el servidor hefora.net, este servidor de respaldo funciona con tecnología en la nube sobre OpenStack y el proveedor tampoco tiene acceso al sistema. La administración de este servidor también se realiza bajo el servicio OpenSSH con autenticación por contraseña o clave privada RSA.

- En el nivel de la base de datos, todos los datos personales y médicos se almacenan con cifrado a nivel de registro, lo que significa que todo es incomprensible sin antes descifrarlo. Además, los registros médicos están debidamente seudoanonimizados por lo que no es posible relacionar directamente a la persona física y sus datos médicos. Además, todos los documentos intercambiados con los usuarios (ej. videos) están encriptados a

través de un proveedor de seguridad externo, chino.io, que tiene la certificación ISO 27001. Para fines de procesamiento y exportación, todos los datos se anonimizan convenientemente".

- Respecto a la exportación de cuestionarios: para cada cuestionario y la persona que lo ha rellenado, lo que se hace es generar un identificador encriptado a partir del identificador original de dicha persona, de tal forma que aunque se tuviera acceso a la base de datos original, no se podría saber a qué identificador original hace referencia, y por tanto no podría saberse qué persona rellenó cada cuestionario. De esta forma, con el listado de cuestionarios exportado, puede saberse si 2 cuestionarios distintos pertenecen a la misma persona (tendrá el mismo identificador encriptado), pero no puede saberse a qué persona física corresponde. Es decir, son anónimos.

- En cuanto al proceso concreto de seudoanonimización, lo que se ha hecho es romper el vínculo en bases de datos entre las tablas de pacientes y de datos médicos. No hay un identificador que nos indique a qué paciente corresponde cada historia clínica, son tablas independientes. En otra tabla aparte, sin relación tampoco con las anteriores, lo que se está haciendo es guardar el identificador del paciente encriptado con un algoritmo AES-256 y sus datos personales también encriptados con ese mismo algoritmo. Para conocer a qué persona física corresponde una historia clínica habría que desencriptar dicho identificador, luego no hay forma de, si se tiene acceso a la base de datos, establecer la relación entre paciente e historia clínica sin conocer la clave y algoritmo de desencriptación. Además, en la tabla de historia clínica no se guarda ningún tipo de dato personal identificativo. El resultado es que las historias clínicas están seudoanonimizadas, ya que en su información no aparece ningún tipo de dato personal ni se puede establecer relación directa con su paciente. Y lo mismo para el resto de datos que tienen relación con la historia clínica: cuestionarios, prescripciones, etc.

De acuerdo con el Reglamento (UE) 2016/679 del Parlamento Europeo y del Consejo de 27 de abril de 2016 de Protección de Datos (RGPD) y la Ley Orgánica 3/2018, de 5 de diciembre, de Protección de Datos Personales y garantía de los derechos digitales, el participante queda informado de que el responsable del tratamiento de sus datos personales será FUNDACION UNIVERSIDAD SAN JORGE.

Como participante en el estudio puede ejercitar sus derechos de acceso, modificación, oposición, cancelación, limitación del tratamiento y portabilidad, dirigiéndose al Delegado de Protección de Datos de la Universidad adjuntando a su solicitud de ejercicio de derechos una fotocopia de su DNI o equivalente al domicilio social de USJ sito en Autovía A-23 Zaragoza- Huesca, km. 299, 50830- Villanueva de Gállego (Zaragoza), o la dirección de correo electrónico privacidad@usj.es. Asimismo, tiene derecho a dirigirse a la Agencia Española de Protección de Datos en caso de no ver correctamente atendido el ejercicio de sus derechos.

El participante podrá retirarse del estudio en cualquier momento comunicándoselo al investigador principal, si bien queda informado de que sus datos no podrán ser eliminados para garantizar la validez de la investigación y garantizar el cumplimiento de los deberes legales del responsable.

5.4.- IMPLICACIONES ASISTENCIALES

El centro hospitalario HPNSG únicamente servirá de centro de referencia de donde se reclutarán los pacientes y donde se realizarán las valoraciones pre y post- intervención. El programa de rehabilitación se realizará en el ámbito domiciliario o en el contexto del paciente por lo que no interfiere en el desarrollo clínico del centro. Para los días de valoración el fisioterapeuta evaluador de dicho centro tendrá permiso para la dedicación a este fin, organizando la agenda asistencial previamente de acuerdo con este requisito.

6.-CRONOGRAMA

CRONOGRAMA	MESES										
FASES	ENERO	FEBRERO	MARZO	2º mitad MARZO	ABRIL	2º mitad ABRIL	MAYO	2º mitad MAYO	JUNIO	SEPTIEM-BRE	ENERO
Diseño del estudio											
Creación del protocolo de ejercicios											
Creación de los vídeos educativos											

Fase previa, estudio observacional											
Análisis datos, fase previa observacional											
Selección y aleatorización ensayo clínico											
Evaluación pre-intervención											
Intervención											
Control cada dos semanas telefónica											
Evaluación post-intervención											
Análisis datos iniciales											
Seguimiento a los 3 y 6 meses											
Análisis datos de seguimiento											

7.-ANEXOS

ANEXO I. Mini-Examen Cognoscitivo (MEC)

	PUNTUACIÓN
**ORIENTACIÓN TEMPORAL:**<br>¿En qué día estamos?.................... ¿En qué fecha?.............................. ¿En qué mes?.................. ¿En qué estación?.............................. ¿En qué año?................	_____(5)
**ORIENTACIÓN ESPACIAL:**<br>¿En qué hospital o lugar estamos?.. ¿En qué piso o planta?................ ¿En qué pueblo o ciudad?.......................... ¿En qué provincia, región o autonomía?................ ¿En qué país?................................	_____(5)
**FIJACIÓN:**<br>Repita estas 3 palabras: 'peseta- caballo- manzana'..<br>(*Repetir hasta 5 veces, pero puntuar solo el primer intento)<br>(**Pueden utilizarse series alternativas de palabras cuando se trata de reevaluaciones ej. Libro, queso, bicicleta)	_____(3)
**CONCENTRACIÓN Y CÁLCULO**<br>Si tiene 30 ptas y le van quitando de 3 en 3, ¿cuantas le quedan?............(27)<br>¿y si le quitan otras 3?.........(24)<br>¿y ahora?...........................(21)<br>¿y 3 menos son?.................(18)<br>¿y si le quitan otras 3?..........(15)<br>(Anote un punto cada vez que la diferencia de 3 sea correcta, aunque la anterior fuera incorrecta)	_____(5)
Repita 5-9-2 (hasta que los aprenda). Ahora hacia atrás...<br>(Como alternativa, decirle 'mundo' y que lo repita al revés)	_____(3)
**MEMORIA**<br>¿Recuerda las 3 palabras que le he dicho antes?..	_____(3)
**LENGUAJE Y CONSTRUCCIÓN**<br>Mostrarle un lápiz o un bolígrafo: ¿Qué es esto?<br>Repetirlo con el reloj..	_____(2)
Repita la frase 'En un trigal había 5 perros'..<br>(Repetir hasta 5 veces, pero puntuar solo el primer intento)	_____(1)
Una manzana y una pera son frutas, ¿verdad?<br>¿Qué son un perro y un gato? ;<br>¿Qué son el verde y el rojo?..	_____(2)
Coja este papel con la mano derecha, dóblelo por la mitad y póngalo encima de la mesa	_____(3)
Lea esta frase y haga lo que dice..	_____(1)
Escriba una frase (con sujeto y predicado)..<br>**CIERRE LOS OJOS**	_____(1)
Copie este dibujo...<br>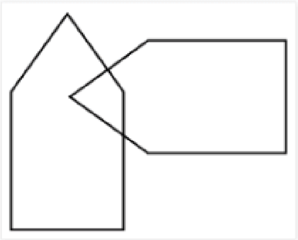	_____(1)
**Puntuación total**	_____(35)

ANEXO II: Folleto inicial de ejercicios.

PLAN DE EJERCICIOS

Indicaciones generales:

- Si aparece **dolor**, disminuir la intensidad o parar el ejercicio y ponerse en contacto con la fisioterapeuta encargada del programa.
- Tres días a la semana.
- **La sensación de fatiga** debe ser moderada 6-7 sobre 10 **cansado, pero puede hablar**.

Aeróbico:

Comenzar con 10 minutos al día y **progresar hasta 30** minutos seguidos al día, luego **aumentar la velocidad** para que siempre se sienta cansado.

Ejercicios nivel 1:

Caminar: en casa o en la calle de forma continua.

Subir escaleras o un escalón.

Ejercicios nivel 2:

Subir escaleras o un escalón.

Caminar rápido.

Nadar.

Ejercicios nivel 3:

Trotar

Saltar en estático

Cualquier otro deporte con la intensidad marcada.

Fuerza-Resistencia:

3 series de tantas repeticiones como se pueda. Descansar 1 minuto entre cada serie.

No deberá mantener el aire, la respiración debe ser continua y al sacar el aire soplando lentamente.

Ejercicios nivel 1:

- **Subir y bajar un peso con las dos manos:** empezar con una botella de 1 litro llena de agua y progresar a la de 2 litros.
- **Sentarse y levantarse** de una silla ayudándose de los brazos.

Ejercicios nivel 2:

- **Agacharse, coger un peso de 1 kilo y levantarlo por encima de la cabeza.**
- **Sentarse y levantarse** de una silla **sin ayuda** de los brazos.

Ejercicios nivel 3:

- **Agacharse, coger un peso de 2 kilos y levantarlo por encima de la cabeza.**
- **Sentadillas.**

Ejercicios de respiración

1.- Sentado en una posición cómoda durante 5 minutos cuantas veces se quiera. Al menos dos al día.

Inspirar lentamente por la nariz.
Guardar el aire 3 segundos.
Soplar lentamente con los labios juntos.

2.- Tumbado de lateral y luego del otro lado durante 5 minutos, al menos dos al día.

Inspirar despacio por la nariz
Sacar el aire de forma lenta con la boca abierta como empañando un cristal.

ANEXO III: Escala de Severidad de la Fatiga (FSS)

Por favor responda del 1 al 7 las siguientes afirmaciones. 1 es que está en total desacuerdo y 7 es que está totalmente de acuerdo.

ESCALA DE INTENSIDAD DE LA FATIGA (KRUPP)	1	2	3	4	5	6	7
1. Mi motivación se reduce cuando estoy fatigado…………………							
2. El ejercicio me produce fatiga………………………………………							
3. Me fatigo fácilmente……………………………………………………							
4. La fatiga interfiere en mi funcionamiento físico…………………							
5. La fatiga me produce con frecuencia problemas…………………							
6. La fatiga me impide hacer ejercicio físico continuado………….							
7. La fatiga interfiere en el desempeño de algunas obligaciones y responsabilidades………………………………………………………							
8. La fatiga es uno de mis 3 síntomas que más me incapacitan….							
9. La fatiga interfiere en mi trabajo, familia o vida social………….							

TOTAL:

ANEXO IV: Encuesta de necesidades.

Buenos días/tardes, necesito que me conteste a las siguientes preguntas:

I.- Datos personales

- Nº id: ……………….
- Nombre: ………………………Apellido… 2º Apellido………………………
- Fecha de nacimiento: día………mes…………año………………
- Vivo
  - Si
  - No
    - Causas derivadas de la COVID-19:
    - Otras causas:
- Género: ……………………………………………

II.- Hábitos de vida

- Fumador:
  - Si
    - Número de cigarrillos al día: ………
  - No

- Ingesta de alcohol:
  - Si
    - Tipo:
      - Cerveza o vino
      - Alcohol alta graduación
    - Vasos a la semana
  - No

- Actividad física semanal:
  - Si
    - Caminar: Días a la semana:
    - Bicicleta estática: Días a la semana
    - Otros:
      - Cual:
      - Días a la semana
    - Total, horas a la semana:
  - No

III.- Características del ingreso

- Días de ingreso hospitalario:
- Forma de estancia:
    - Planta:
    - UCI:
        - Oxígeno:
            - Gafas nasales:
            - Intubación:
        - Sedación:
            - Si
                - Días:
            - No.

- Rehabilitación hospitalaria:
    - Si:
        - Días/semana:
        - Tipo de rehabilitación:
            - Respiratoria.
            - Cardiaca.
            - Neurológica.
            - Movilización.
    - No.

IV.- Consecuencias de la COVID-19

1- ¿Cuál de estos síntomas le ha aparecido que antes no tenía?

- Astenia (falta de apetito):
- Ansiedad:
- Depresión:
- Estrés ante situaciones cotidianas:
- Pérdidas de memoria:
- Cefaleas o migrañas:

  Asociadas al ejercicio:
    - Si
    - No

2- ¿Puede usted vivir solo sin ninguna asistencia constante de otra personal?, por ejemplo, es capaz de comer, caminar, usar el baño y manejar rutinas diarias de higiene de manera independiente.

- Si (si marca si, continúe la encuesta en el punto 3)
- No (si es no, conteste la siguiente pregunta y pase al punto 6)

2.1.- Si ha marcado que no puede vivir sin asistencia personal indique en que aspectos usted necesita una ayuda esencial y constante: (continúe la encuesta en el punto 6)

- Comer
- Usar el baño
- Higiene diaria
- Caminar
- Tareas domésticas
- Viajes locales

3.- ¿Hay tareas/ actividades las cuales ya no es capaz de realizar por sí mismo?, no necesita ayuda constante, pero si para algunas actividades de su vida.

- ○ Si (si es si, conteste la siguiente pregunta y pase al punto 6)
- ○ No (si marca no, continúe la encuesta en el punto 4)

3.1.- Si ha marcado que hay tareas que no puede realizar por si mismo, pero no necesita ayuda constante, marque que tareas no puede hace por si mismo y que necesita modificar o ayuda:

- Compras locales
- Tareas del hogar
- Trabajo
- Cuidado de los seres queridos

- ¿Qué en concreto ya no puede hacer igual?
- ¿Porqué siente que no puede hacerlo?

4.- ¿Necesita usted evitar o reducir tareas/actividades o extender estas tareas en el tiempo? Puede usted hacer lo mismo de antes, pero con modificaciones o alguna cosa en concreto no puede hacerla.

- ○ Si (si es si, conteste la siguiente pregunta y pase al punto 6)
- ○ No (si marca no, continúe la encuesta en el punto 5)

4.1.- Si ha marcado que es autónomo, pero necesita modificar algunas tareas, responda cuales.

- Hogar
- Trabajo
- Participación social o de ocio

▪ ¿Qué en concreto ya no puede hacer igual?
▪ ¿Porqué siente que no puede hacerlo?

5.- ¿Sufre usted síntomas como dolor, depresión o ansiedad?

o Si
o No

6.- ¿Tiene síntomas a través de los cuales es necesario evitar o reducir las tareas? ¿Tiene usted síntomas como resultado de la COVID-19, pero sin experimentar limitaciones funcionales?

o Si
  ▪ ¿Qué tareas hace con limitaciones?.....................................
  ▪ ¿Cuál es la causa de la limitación?.....................................
o No

7.- ¿Tiene problemas para relajarse y/o ve el COVID-19 como un trauma?

o Si
o No

8- De 1: totalmente desacuerdo, a 7 totalmente en de acuerdo indique:

- Mi motivación es baja cuando estoy fatigado:
- El ejercicio me produce fatiga:
- Me fatigo fácilmente:
- La fatiga interfiere en mi funcionamiento físico:

- La fatiga me causa frecuentes problemas:

- Mi fatiga me impide realizar una actividad física mantenida:
- La fatiga está entre mis tres síntomas más discapacitantes:
- La fatiga interfiere en mi trabajo, vida familiar o vida social:

9.- ¿Siente usted falta de fuerza en los brazos o piernas al realizar las actividades diarias?

- Si
    - ¿En qué actividades?...

- No

10.- ¿Tiene usted dolor durante el día o la noche?

- Si
    - De 0=nada a 10=el máximo imaginable, cuánto:
    - Le impide realizar actividades habituales que antes realizada:
        - Si
        - No

- No

11.- ¿Cuál es el síntoma/s que más le afecta a su vida diaria, desde que fue diagnosticado de COVID-19?

………………………………………………………………………………………
………………………………………………………………………………………
……………………………………………………………………………………

12.- En relación a las secuelas que presenta por haber sufrido la COVID-19, ¿cuál sería la necesidad actual que considera que necesitaría ser abordada desde el punto de vista de la rehabilitación?

………………………………………………………………………………………
………………………………………………………………………………………
………………………………………………………………………………………

ANEXO V: Prueba de marcha de 6 minutos.

Debe usted caminar, sin correr, la máxima distancia durante 6 minutos seguidos. Si siente fatiga puede usted parar cuanto necesite.

PRUEBA DE MARCHA DE 6 MINUTOS (6MWT)

Nombre   Fecha   Sexo (H/M)

Edad (años)   Peso (Kg)   Talla (m)   FC máx [210-(edad x 0,65)]

Diagnóstico   Examinador

Medicación (incluir dosis y horario)

6MWT Nº1		30	metros	
		BASAL		FINAL
SaO2			(%)	
FC			(ppm)	
Disnea			(Borg)	
Fatiga EEII			(Borg)	
Tensión Arterial			mmHg	
Vueltas	Metros	Tiempo	SaO2	FC
1	30			
2	60			
3	90			
4	120			
5	150			
6	180			
7	210			
8	240			
9	270			
10	300			
11	330			
12	360			
13	390			
14	420			
15	450			
16	480			
17	510			
18	540			
19	570			
20	600			
21	630			
22	660			
23	690			
24	720			
25	750			
26	780			
27	810			

Incentivo	
min 1	*"Lo está haciendo muy bien, faltan 5 minutos"*
min 2	*"Perfecto, continúe así, faltan 4 minutos"*
min 3	*"Está en la mitad del tiempo de la prueba, lo está haciendo muy bien"*
min 4	*"Perfecto, continúe así, faltan dos minutos"*
min 5	*"Lo está haciendo muy bien, falta un minuto"*
min 5:45	*"Deberá detenerse cuando se lo indique"*
min6	***PARE***

PARADAS	TIEMPO AL PARAR	TIEMPO AL REANUDAR
1		
2		
3		

RECUPERACION			
	SaO 2	*FC*	*TA*
min1			
min2			
min3			
min4			
min5			

Distancia adicional	
Distancia total caminada	

ANEXO VI: Test de 30-segundos sentarse y levantarse.

Por favor, con las manos en cruz sobre los hombros y los pies apoyados en el suelo, levántese completamente y vuelva a la posición inicial todas las veces que pueda en 30 segundos.

Yo le indicaré el comienzo a la señal de "ya" y al finalizar le diré: "pare".

ANEXO VII: Test de flexión de codo en 30 segundos.

Por favor, coja la pesa de 2 kilos con su mano dominante, sentado y con los pies apoyados en el suelo. Lleve la pesa al hombro y vuelva a la posición inicial todas las veces que pueda en 30 segundos.

Yo le indicaré el comienzo a la señal de “ya” y al finalizar le diré: “pare”.

ANEXO VIII: Escala del estado funcional Post-Covid.

EVALUACIÓN DE LA ESCALA

**Nombre / ID paciente**	
**Fecha de diagnóstico de COVID-19**	
**Fecha de evaluación de la escala PCFS**	
**Lugar**	Al alta<br>Visita ambulatoria a las 4 semanas<br>Visita ambulatoria a las 8 semanas<br>Visita ambulatoria a los 6 meses<br>Otra (especifique) ________
**Encuestado**	Paciente<br>Paciente y otra persona<br>Sólo otra persona<br>Especifique ______
**Evaluador**	Médico<br>Personal del estudio

Por favor, conteste con sí o no a las siguientes preguntas.

ENTREVISTA ESTRUCTURADA

**1. SOBREVIDA**	**Calificación correspondiente en la escala PCFS si respuesta es "SI"**
**1.1 ¿Ha fallecido el paciente después del diagnóstico de COVID-19?**	**D**

**2. CUIDADO CONSTANTE**<br>*Explicación*: significa que alguien más debe estar a su disposición todo el tiempo. El cuidado puede ser proporcionada por un cuidador entrenado o no entrenado. El paciente generalmente estará postrado en la cama y puede tener incontinencia.	**Calificación correspondiente en escala PCFS si respuesta es "SI"**
**2.1 ¿Requiere usted cuidados contantes?**	**4**

**3. ACTIVIDADES BÁSICAS DE LA VIDA DIARIA (ABVD)**<br>*Explicación:* la asistencia incluye la ayuda física, instrucción verbal o supervisión de otra persona. Puede considerarse esencial cuando hay una necesidad de ayuda física (por parte de otra persona) con una actividad o para supervisión, o cuando el paciente necesita indicaciones o recordatorios para realizar una tarea. La necesidad de supervisión por razones de seguridad debería obedecer a un peligro objetivo que se presenta, y no "por si acaso".	**Calificación correspondiente en escala PCFS si respuesta es "SI"**
**3.1 ¿Es esencial la asistencia para comer?**<br>(Comer sin ayuda: la comida y los utensilios pueden ser proporcionados por otros)	**4**
**3.2 ¿Es esencial la asistencia para usar el baño?**<br>(Usar el baño sin ayuda: llegar al baño/inodoro; desvestirse lo suficiente; limpiarse; vestirse y salir)	**4**

**3.3 ¿Es esencial la asistencia para la rutina de higiene diaria?**<br>(La rutina de higiene incluye sólo lavarse la cara, peinarse s, lavarse los dientes y colocarse la dentadura postiza. Los implementos pueden ser proporcionados por otros sin considerar esto como asistencia)	**4**
**3.4 ¿Es esencial la asistencia para caminar?**<br>(Caminar sin asistencia: si es absolutamente necesario, es capaz de caminar en el interior o alrededor de la casa o sala, puede utilizar cualquier ayuda, sin embargo, no requiere ayuda física o instrucción verbal o supervisión de otra persona)	**4**

**4. ACTIVIDADES INSTRUMENTALES DE LA VIDA DIARIA (AIVD)**<br>*Explicación*: la asistencia incluye la ayuda física, instrucción verbal o supervisión de otra persona. Puede considerarse esencial cuando hay una necesidad de ayuda física (por parte de otra persona) con una actividad o para supervisión, o cuando el paciente necesita indicaciones o recordatorios para realizar una tarea. La necesidad de supervisión por razones de seguridad debería obedecer a un peligro objetivo que se presenta, y no "por si acaso".	**Calificación correspondiente en escala PCFS si respuesta es "SI"**
**4.1 ¿Es esencial la asistencia para las tareas domésticas básicas que son importantes para la vida diaria?**<br>(Por ejemplo: preparar una comida sencilla, lavar los platos, sacar la basura; excluya tareas que no necesitan ser realizadas todos los días)	**4**
**4.2 ¿Es esencial la asistencia para los viajes locales?**<br>(Viajes locales sin asistencia: el paciente puede conducir o utilizar el transporte público para desplazarse. La posibilidad de utilizar un taxi es suficiente, siempre que el paciente pueda llamar e indicarle al conductor)	**4**
**4.3 ¿Es esencial la asistencia para las compras locales?**<br>(El paciente no es capaz de comprar alimentos o artículos de primera necesidad por sí mismo)	**3**

5. PARTICIPACIÓN EN ROLES SOCIALES HABITUALES<br>*Explicación:* esta sección se refiere al disminución en el cumplimiento de los principales roles sociales (no las circunstancias sociales o financieras).	Calificación correspondiente en escala PCFS si respuesta es "SI"
**5.1 ¿Es esencial adaptar las tareas/actividades en el hogar o en el trabajo/estudio porque usted no puede realizarlas por sí mismo (por ejemplo, produciendo un cambio en el nivel de responsabilidad, un cambio de de tiempo completo a tiempo parcial en el trabajo, o un cambio en la educación)?**<br>(El trabajo se refiere tanto al empleo remunerado como al trabajo voluntario. Las adaptaciones especiales que permiten a alguien volver a trabajar, aunque normalmente no podría hacerlo, deben considerarse como una adaptación del trabajo).	3
**5.2 ¿Necesita usted ocasionalmente evitar o reducir las tareas/actividades en el hogar o en el trabajo/estudio o necesita extenderlas a lo largo del tiempo (aunque básicamente usted sea capaz de realizar todas esas actividades)?**	2
**5.3 ¿Ya no puede cuidar bien de sus seres queridos como antes?**<br>(Cuidar bien incluye cuidar a su pareja, padres, nietos u otras personas dependientes).	3
**5.4 Desde el diagnóstico de COVID-19, ¿ha habido problemas en sus relaciones o se ha aislado?**<br>(Estos problemas incluyen problemas de comunicación, dificultades en las relaciones con las personas en casa o en el trabajo/estudio, pérdida de amistades ,(aumento del) aislamiento, etc.)	3
**5.5 ¿Está restringida su participación en actividades sociales y de ocio?**<br>(Incluye pasatiempos e intereses, incluyendo ir a un restaurante, bar, cine, salir a caminar, juegos, lectura de libros, etc.)	2

**6. LISTA DE CHEQUEO DE SÍNTOMAS**<br>*Explicación:* estos pueden ser cualquier síntoma o problema informado por los pacientes o encontrado en el examen físico. Los síntomas incluyen, pero no se limitan a: disnea, dolor, fatiga, debilidad muscular, pérdida de memoria, depresión y ansiedad.	**Calificación correspondiente en escala PCFS si respuesta es "SI"**
**6.1 ¿Presenta usted síntomas por los cuales se deben evitar, reducir o extender las tareas/actividades habituales a lo largo del tiempo?**	2
**6.2 ¿Presenta usted algún síntoma, resultante de COVID-19, sin experimentar limitaciones funcionales?**	1
**6.3 ¿Tiene usted problemas para relajarse o experimenta el COVID-19 como un trauma?**<br>('Trauma' es definido como: sufrir recuerdos intrusivos, recuerdos recurrentes o respuestas evitativas, asociadas a haber experimentado el COVID-19.)	1

Asignación de la calificación en la escala de estado funcional post-COVID-19

La clasificación general es simplemente el peor estado funcional indicado por las respuestas del paciente (la calificación más alta corresponde a mayores limitaciones). Si un paciente no tiene limitaciones o síntomas, entonces la calificación correspondiente en la escala es 0.

Calificación final de la Escala PCFS: _______

¿Cuál era su grado de escala PCFS antes del COVID-19? ________

Figura 1: Flujograma para auto reporte del paciente de la Escala de estado funcional post COVID19

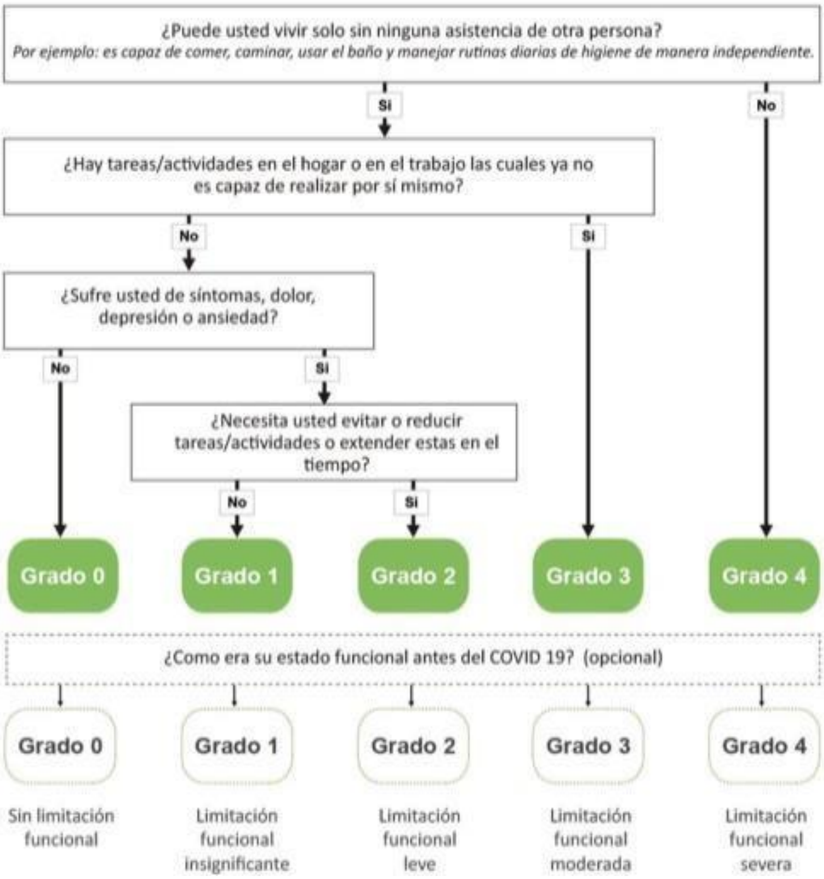

ANEXO IX: Escala de autoeficacia general abreviada.

Puntúe de 1= nunca le ocurre a 5= siempre le ocurre, las siguientes afirmaciones:

Item
1.A pesar de los obstáculos yo puedo encontrar las maneras de obtener lo que quiero
2. Yo enfrento problemas graves si me esfuerzo lo suficiente
3. Es fácil mantenerme en mis metas hasta alcanzarlas
4. Me siento seguro de poder enfrentar eficazmente situaciones inesperadas
5. Gracias a mis cualidades personales yo puedo enfrentar situaciones inesperadas
6. Yo puedo mantener la calma cuando estoy en problemas porque confío en mis habilidades para enfrentarlos
7. Venga lo que venga, por lo general soy capaz de manejarlo
8. Yo puedo resolver la mayoría de los problemas si me esfuerzo lo necesario
9. En una situación difícil, generalmente se me ocurre lo que debo hacer
10. Cuando me enfrento a un problema, generalmente encuentro varias soluciones

ANEXO X: Escala de depresión y ansiedad.

DASS-21

Por favor lea las siguientes afirmaciones y coloque un círculo alrededor de un número (0, 1, 2, 3) que indica en qué grado le ha ocurrido a usted esta afirmación *durante la semana pasada*. La escala de calificación es la siguiente:

0: No me ha ocurrido; 1: Me ha ocurrido un poco, o durante parte del tiempo; 2: Me ha ocurrido bastante, o durante una buena parte del tiempo; 3: Me ha ocurrido mucho, o la mayor parte del tiempo.

1.	Me ha costado mucho descargar la tensión	0	1	2	3
2.	Me di cuenta que tenía la boca seca	0	1	2	3
3.	No podía sentir ningún sentimiento positivo	0	1	2	3
4.	Se me hizo difícil respirar	0	1	2	3
5.	Se me hizo difícil tomar la iniciativa para hacer cosas	0	1	2	3
6.	Reaccioné exageradamente en ciertas situaciones	0	1	2	3
7.	Sentí que mis manos temblaban	0	1	2	3
8.	He sentido que estaba gastando una gran cantidad de energía	0	1	2	3
9.	Estaba preocupado por situaciones en las cuales podía tener pánico o en las que podría hacer el ridículo	0	1	2	3
10.	He sentido que no había nada que me ilusionara	0	1	2	3
11.	Me he sentido inquieto	0	1	2	3
12.	Se me hizo difícil relajarme	0	1	2	3
13.	Me sentí triste y deprimido	0	1	2	3
14.	No toleré nada que no me permitiera continuar con lo que estaba haciendo....	0	1	2	3
15.	Sentí que estaba al punto de pánico	0	1	2	3
16.	No me pude entusiasmar por nada..	0	1	2	3
17.	Sentí que valía muy poco como persona	0	1	2	3
18.	He tendido a sentirme enfadado con facilidad	0	1	2	3
19.	Sentí los latidos de mi corazón a pesar de no haber hecho ningún esfuerzo físico	0	1	2	3
20.	Tuve miedo sin razón	0	1	2	3
21.	Sentí que la vida no tenía ningún sentido..	0	1	2	3

ANEXO XI: Cuestionario de regulación conductual en el deporte

Cuestionario de Regulación de Conducta en el Deporte (BRSQ) Lonsdale, Hodge, y Rose (2008)

**Participo en este deporte…**	Nada es verdad	Bastante en desacuerdo	Algo en desacuerdo	Neutro	Algo de verdad	Bastante de verdad	Muy verdadero
Porque lo disfruto	❶	❷	❸	❹	❺	❻	❼
Por el placer que me da el conocer más acerca de este deporte	❶	❷	❸	❹	❺	❻	❼
Porque me encantan los estímulos intensos que puedo sentir mientras practico este deporte	❶	❷	❸	❹	❺	❻	❼
Porque disfruto cuando intento alcanzar metas a largo plazo	❶	❷	❸	❹	❺	❻	❼
Porque es parte de lo que soy	❶	❷	❸	❹	❺	❻	❼
Porque los beneficios del deporte son importantes para mi	❶	❷	❸	❹	❺	❻	❼
Porque me sentiría avergonzado si lo abandono	❶	❷	❸	❹	❺	❻	❼
Porque si no lo hago otros no estarían contentos conmigo	❶	❷	❸	❹	❺	❻	❼
Sin embargo, no se por qué lo hago	❶	❷	❸	❹	❺	❻	❼
Porque me gusta	❶	❷	❸	❹	❺	❻	❼
Porque me gusta aprender cómo usar nuevas técnicas	❶	❷	❸	❹	❺	❻	❼
Por el entusiasmo que siento cuando estoy implicado en la actividad	❶	❷	❸	❹	❺	❻	❼
Porque disfruto mientras trabajo algo importante	❶	❷	❸	❹	❺	❻	❼
Porque es una oportunidad de ser quien soy	❶	❷	❸	❹	❺	❻	❼
Porque me enseña disciplina	❶	❷	❸	❹	❺	❻	❼
Porque me sentiría haber fallado si lo abandono	❶	❷	❸	❹	❺	❻	❼
Porque otros me presionan a jugar	❶	❷	❸	❹	❺	❻	❼
Sin embargo, me cuestiono por qué continuo	❶	❷	❸	❹	❺	❻	❼
Porque me divierto	❶	❷	❸	❹	❺	❻	❼
Porque disfruto aprendiendo nuevas técnicas	❶	❷	❸	❹	❺	❻	❼
Por el placer que me da cuando estoy totalmente entregado en este deporte	❶	❷	❸	❹	❺	❻	❼
Porque disfruto mientras hago algo lo mejor que puedo	❶	❷	❸	❹	❺	❻	❼
Porque el practicar este deporte es parte de quien soy	❶	❷	❸	❹	❺	❻	❼
Porque aprecio los beneficios de este deporte	❶	❷	❸	❹	❺	❻	❼
Porque me siento obligado a continuar	❶	❷	❸	❹	❺	❻	❼
Porque otros me empujan a jugar	❶	❷	❸	❹	❺	❻	❼
Sin embargo, las razones de por qué practico no las tengo claras	❶	❷	❸	❹	❺	❻	❼
Porque lo encuentro agradable	❶	❷	❸	❹	❺	❻	❼
Me gusta aprender cosas nuevas acerca de este deporte	❶	❷	❸	❹	❺	❻	❼
Por los sentimientos positivos que siento mientras practico este deporte	❶	❷	❸	❹	❺	❻	❼
Porque me produce satisfacción cuando me esfuerzo por alcanzar mis metas	❶	❷	❸	❹	❺	❻	❼
Porque me permite vivir de acuerdo con mis valores	❶	❷	❸	❹	❺	❻	❼
Porque es una manera muy buena de aprender cosas que pueden ser de gran utilidad en mi vida diaria	❶	❷	❸	❹	❺	❻	❼
Porque me sentiría culpable si lo abandono	❶	❷	❸	❹	❺	❻	❼
Para satisfacer a los que quieren que juegue	❶	❷	❸	❹	❺	❻	❼
Sin embargo, me pregunto por qué me esfuerzo para esto	❶	❷	❸	❹	❺	❻	❼

ANEXO XII: Escala multidimensional de apoyo social percibido

ESCALA MULTIDIMENSIONAL DE APOYO SOCIAL PERCIBIDO (EMAS)

Lee cada una de las siguientes frases cuidadosamente. Indica tu acuerdo con cada una de ellas empleando esta escala:

1	2	3	4	5	6	7
Totalmente en desacuerdo	Bastante en desacuerdo	Más Bien en desacuerdo	Ni de acuerdo ni en desacuerdo	Más bien de acuerdo	Totalmente en desacuerdo	Totalmente de Acuerdo

1.	Hay una persona que está cerca cuando estoy en una situación difícil.	1	2	3	4	5	6	7
2.	Existe una persona especial con la cual yo puedo compartir penas y alegrías.	1	2	3	4	5	6	7
3.	Mi familia realmente intenta ayudarme	1	2	3	4	5	6	7
4.	Obtengo de mi familia la ayuda y el apoyo emocional que necesito.	1	2	3	4	5	6	7
5.	Existe una persona que realmente es una fuente de bienestar para mí.	1	2	3	4	5	6	7
6.	Mis amigos realmente tratan de ayudarme	1	2	3	4	5	6	7
7.	Puedo contar con mis amigos cuando las cosas van mal	1	2	3	4	5	6	7
8.	Yo puedo hablar de mis problemas con mi familia	1	2	3	4	5	6	7
9.	Tengo amigos con los que puedo compartir las penas y alegrías	1	2	3	4	5	6	7
10.	Existe una persona especial en mi vida que se preocupa por mis sentimientos	1	2	3	4	5	6	7
11.	Mi familia se muestra dispuesta a ayudarme para tomar decisiones	1	2	3	4	5	6	7
12.	Puedo hablar de mis problemas con mis amigos.	1	2	3	4	5	6	7

ANEXO XIII: Cuestionario de calidad de vida.

CUESTIONARIO DE SALUD SF-12

INSTRUCCIONES: Las preguntas que siguen se refieren a lo que usted piensa sobre su salud. Sus respuestas permitirán saber como se encuentra usted y hasta qué punto es capaz de hacer sus actividades habituales.

Por favor, conteste cada pregunta marcando una casilla. Si no está seguro/a de cómo responder a una pregunta, por favor, conteste lo que le parezca más cierto.

1. En general, usted diría que su salud es:

1	2	3	4	5
☐	☐	☐	☐	☐
**Excelente**	**Muy buena**	**Buena**	**Regular**	**Mala**

Las siguientes preguntas se refieren a actividades o cosas que usted podría hacer en un día normal. Su salud actual, ¿le limita para hacer esas actividades o cosas? Si es así, ¿cuánto?

	1<br>**Sí, me limita mucho**	2<br>**Sí, me limita un poco**	3<br>**No, no me limita nada**
2. **Esfuerzos moderados,** como mover una mesa, pasar la aspiradora, jugar a los bolos o caminar más de 1 hora	☐	☐	☐
3. Subir **varios** pisos por la escalera	☐	☐	☐

Durante **las 4 últimas semanas,** ¿ha tenido alguno de los siguientes problemas en su trabajo o en sus actividades cotidianas, **a causa de su salud física?**

	1<br>**Sí**	2<br>**No**
4. ¿**Hizo menos** de lo que hubiera querido hacer?	☐	☐
5. ¿Tuvo que **dejar de hacer algunas tareas** en su trabajo o en sus actividades cotidianas?	☐	☐

Durante las **4 últimas semanas,** ¿ha tenido alguno de los siguientes problemas en su trabajo o en sus actividades cotidianas, **a causa de algún problema emocional** (como estar triste, deprimido, o nervioso)?

	1<br>**Sí**	2<br>**No**
6. ¿**Hizo menos** de lo que hubiera querido hacer, **por algún problema emocional**?	☐	☐
7. ¿No hizo su trabajo o sus actividades cotidianas tan **cuidadosamente** como de costumbre, **por algún problema emocional**?	☐	☐

8. Durante las **4 últimas semanas,** ¿hasta qué punto **el dolor** le ha dificultado su trabajo habitual (incluido el trabajo fuera de casa y las tareas domésticas)?

1	2	3	4	5
☐	☐	☐	☐	☐
**Nada**	**Un poco**	**Regular**	**Bastante**	**Mucho**

Las preguntas que siguen se refieren a cómo se ha sentido y cómo le han ido las cosas durante las **4 últimas semanas.** En cada pregunta responda lo que se parezca más a cómo se ha sentido usted. Durante las **4 últimas semanas** ¿cuánto tiempo...

	1 **Siempre**	2 **Casi siempre**	3 **Muchas veces**	4 **Algunas veces**	5 **Sólo alguna vez**	6 **Nunca**
9. ...se sintió calmado y tranquilo?	❑	❑	❑	❑	❑	❑
10. ...tuvo mucha energía?	❑	❑	❑	❑	❑	❑
11. ...se sintió desanimado y triste?	❑	❑	❑	❑	❑	❑

12. Durante las **4 últimas semanas,** ¿con qué frecuencia la salud física o los problemas emocionales le han dificultado sus actividades sociales (como visitar a los amigos o familiares)?

1	2	3	4	5
❑	❑	❑	❑	❑
**Siempre**	**Casi**	**Algunas siempre**	**Sólo veces**	**Nunca alguna vez**

ANEXO XIV: Diario de seguimiento de adherencia a la actividad (para medir las variables adherencia y compromiso)

Semanario	Sem 1			Sem 2			Sem 3		
Diario	1	2	3	1	2	3	1	2	3
He hecho todos los ejercicios según las indicaciones									
He hecho todos, pero no todas las repeticiones									
He hecho algunos ejercicios completos según las indicaciones									
He hecho algunos ejercicios y no todas las repeticiones									
No los he hecho									
He hecho más ejercicio voluntario de lo recomendado									
Andar más de 30 minutos									
Correr más de 30 minutos									
Otros ejercicios adicionales:									
Describa cuales									

ANEXO XV: Análisis de satisfacción con el programa y la plataforma.

- Por favor, valore de 0 a 4 su satisfacción general, siendo 0=muy insatisfecho y 4=muy satisfecho, los siguientes aspectos:

- El programa de ejercicios…………
- La atención recibida durante el programa…………

- Grupo experimental:

- La plataforma Hefora……………….
- Los vídeos de ejercicios………………
- Las explicaciones de los ejercicios…………….
- Los vídeos educativos……………….

- Por favor, indíqueme si durante estos 15 días has tenido usted algún problema para realizar el programa de ejercicios:
    - Si
        - ¿Lo ha podido solucionar?
            - Si
            - No
        - ¿Le ha impedido realizar el programa?
            - Si
            - No
    - No

- Grupo Control:

- El folleto explicativo…………..

ANEXO XVI: Hoja de Información al Paciente y Consentimiento Informado

DOCUMENTO DE INFORMACIÓN PARA EL PARTICIPANTE

Título de la investigación: Eficacia de un programa de telerehabilitación asincrónico en pacientes postCOVID-19: estudio de viabilidad.

Promotor: Universidad San Jorge.

Investigador Principal: Sandra Calvo Carrión y Carolina Jiménez Sánchez Tfno: 661 833 194 / 649 612 644 mail: scalvo@usj.es / cjimenez@usj.es

Centro: Universidad San Jorge- Hospital Provincial Nuestra Señora de Gracia de Zaragoza.

1. Introducción:

Nos dirigimos a usted para solicitar su participación en un proyecto de investigación que estamos realizando desde la Universidad San Jorge en colaboración con el Hospital Provincial Nuestra Señora de Gracia y el Hospital Royo Villanova de Zaragoza. Su participación es voluntaria, pero este estudio es importante para obtener el conocimiento que necesitamos. Este proyecto ha sido aprobado por el Comité de Ética de Aragón. Antes de tomar una decisión es necesario que usted por favor:

- lea este documento entero
- entienda la información que contiene el documento
- haga todas las preguntas que considere necesarias
- tome una decisión meditada
- firme el consentimiento informado, si finalmente desea participar

Si decide participar se le entregará una copia de esta hoja y del documento de consentimiento firmado. Por favor, consérvelo por si lo necesitara en un futuro.

2. ¿Por qué se le pide participar?

Se le solicita su colaboración porque usted ha padecido la COVID-19 y ha sido ingresado en el Hospital Provincial Nuestra Señora de Gracia o en el Hospital Royo Villanova de

Zaragoza y se le proponer, a su alta, participar en un estudio piloto donde se realice un programa domiciliario compuesto de una serie de ejercicios y recomendaciones terapéuticas para que usted mejore su condición física y calidad de vida.

En total en el estudio participarán 32 pacientes previamente ingresados en el Hospital Provincial Nuestra Señora de Gracia de Zaragoza y que han sido dados de alta.

3. ¿Cuál es el objeto de este estudio?

El estudio nos permitirá valorar si la prescripción de un programa de ejercicios y educación para la salud que se realizan de forma domiciliaria mediante una aplicación web o mediante información escrita, ayuda a mejorar la condición física y la calidad de vida de las personas que han pasado la COVID-19 y han sido dadas de alta hospitalaria.

4. ¿Qué tengo que hacer si decido participar?

El programa consistirá en la realización de un programa de ejercicios y educación para la salud en pacientes con secuelas post-COVID19. El grupo intervención recibirá un programa de ejercicios y recomendaciones educativas a través de la plataforma web HEFORA que ofrece gratuitamente el Colegio Profesional de Fisioterapeutas de Aragón. El grupo control recibirá el mismo programa de ejercicios y recomendaciones educativas en forma de folleto explicativo con la información para poder seguir el programa.

Se requiere evaluar su historia clínica que se encuentra en el Hospital Provincial Nuestra Señora de Gracia de Zaragoza). Si desea participar, cumple los criterios de inclusión, y firma el consentimiento informado, se le citará, y en el Hospital Provincial Nuestra Señora de Gracia se le realizará una evaluación física que deberá repetir transcurridas 12 semanas al final del estudio (**dos valoraciones** con una duración de 30 minutos aproximadamente cada una de ellas). Es importante que conozca, que para estas dos valoraciones habrá de desplazarse al Hospital Provincial Nuestra Señora de Gracia, y que dichos desplazamientos (2 en total: uno al inicio de la intervención y otro justo tras su finalización; no serán compensados. Este estudio no tiene financiación por lo que dichos desplazamientos no pueden ser asumidos).

Además, durante el tiempo que dure el estudio se le pedirá que complete un diario bien de forma escrita o bien virtual mediante una plataforma web donde deberá registrar si ha completado los ejercicios programados, si los ha realizado con la carga (peso y repeticiones) indicadas, si ha realizado ejercicio adicional al pautado y su nivel de fatiga (de 0 a 10) al terminarlos.

También se realizará un **control telefónico cada dos semanas** para solucionar posibles problemas con la realización, valorar el nivel de fatiga durante la realización del programa y analizar posibles incidencias.

El programa tendrá una duración total de 13 semanas desde que se le asigne un grupo y realice la primera evaluación hasta la valoración final y de 6 meses hasta pasados los dos seguimientos.
Todas las sesiones de evaluación, intervención u seguimiento se realizarán de forma gratuita para todos los participantes. El único gasto asumido por parte del participante será el derivado por el desplazamiento al Hospital Provincial Nuestra Señora de Gracia de Zaragoza donde se llevarán a cabo las valoraciones presenciales.

5. ¿Qué riesgos o molestias supone?
La realización de un programa de ejercicio terapéutico y educación para la salud no presenta efectos adversos si se realiza siguiendo las recomendaciones pautadas. Cada paciente tendrá el teléfono de los investigadores principales en caso de cualquier incidencia y podrá comunicar telefónicamente con los investigadores para cualquier duda o circunstancia adversa durante el tratamiento. La plataforma web HEFORA cuenta además con un canal de mensajería donde reflejar cualquier circunstancia de esta índole.

6. ¿Obtendré algún beneficio por mi participación?
Al tratarse de un estudio de investigación orientado a generar conocimiento no obtendrá ningún beneficio por su participación, si bien, usted contribuirá al avance científico y al beneficio social. Usted no recibirá ninguna compensación económica por su participación.

7. ¿Cómo se van a tratar mis datos personales? Información básica sobre protección de datos. Responsable del tratamiento: Universidad San Jorge.
Finalidad: Sus datos personales serán tratados exclusivamente para el trabajo de investigación a los que hace referencia este documento.
Legitimación: El tratamiento de los datos de este estudio queda legitimado por su consentimiento a participar.

Destinatarios: No se cederán datos a terceros salvo obligación legal.

Derechos: Como participante en el estudio puede ejercitar sus derechos de acceso, modificación, oposición, cancelación, limitación del tratamiento y portabilidad, dirigiéndose al Delegado de Protección de Datos de la Universidad adjuntando a su solicitud de ejercicio de derechos una fotocopia de su DNI o equivalente al domicilio social de USJ sito en Autovía A-23 Zaragoza- Huesca, km. 299, 50830- Villanueva de Gállego (Zaragoza), o la dirección de correo electrónico privacidad@usj.es. Como usuario de la plataforma HEFORA tiene derecho a acceder, rectificar y suprimir los datos, así como otros derechos indicados en la información adicional a través de dirección de correo electrónico info@hefora.com. La plataforma recogerá de forma anónima y encriptada las veces que ha accedido a la plataforma y las veces que ha visualizado los vídeos. Estos datos los guardará hasta el final del estudio, momento en que los cederá a los investigadores principales para su análisis, únicamente con fines de investigación y manteniendo en todo momento el anonimato de los participantes.

La recogida de datos se realizará mediante los diarios en papel y a través de la aplicación web HEFORA (puede consultar toda la política de privacidad en https://es.hefora.com/privacy-and-cookie-policies "política de privacidad".

Al acceder por primera vez a la plataforma, deberá marcar la casilla para verificar si acepta la política de privacidad y protección de datos de la plataforma.

La plataforma HEFORA en primer lugar recoge unos datos iniciales para crearse la cuenta como usuario, que son un nombre de usuario, una contraseña, un email y el nombre y apellidos. Una vez que el paciente tiene su cuenta creada, contacta con el investigador aceptando de forma digital el documento de autorización para tratamiento de datos personales en el estudio y una vez establecido ese vínculo el

investigador puede incluir en la plataforma los datos que recoja él directamente o acceder a los resultados de los cuestionarios autoadministrados por el paciente (datos descritos en el proyecto de investigación). La fecha y hora de firma queda registrada y puede ser consultada tanto por el paciente como por el investigador.

Los datos que posteriormente a esta aceptación pueden llegarse a recoger en la plataforma si el usuario decide rellenarlos serán, en función del usuario (investigador/profesional o paciente):
- Profesionales: DNI/NIF, Nombre y apellidos, dirección postal o electrónica, teléfono, imagen, firma manual, características personales, circunstancias sociales, académicos y profesionales, detalles de empleo, información comercial, económicos, financieros y de seguro, transacciones de bienes y servicios.

- Usuarios: DNI/NIF, Nombre y apellidos, dirección postal o electrónica, teléfono, imagen, firma manual, características personales (personalidad o comportamiento), características personales, circunstancias sociales, académicos y profesionales, detalles de empleo, información comercial, económicos, financieros y de seguro, transacciones de bienes y servicios.

- Pacientes: DNI/NIF, Nombre y apellidos, dirección postal o electrónica, teléfono, imagen, firma manual, marcas físicas, salud, características personales (personalidad o comportamiento), características personales, circunstancias sociales, académicos y profesionales, detalles de empleo, información comercial, económicos, financieros y de seguro, transacciones de bienes y servicios.

2.- Sobre los datos que conserva, mientras que dure la investigación, los datos conservados serán los indicados anteriormente. Si finaliza el servicio se suprimen salvo los que haya que mantener por obligación legal. Y para un uso ulterior, investigación, estos datos se encuentran anonimizados y por tanto no son personales. El usuario tiene la posibilidad de eliminar su cuenta también una vez finalice el estudio.

3.- Sobre la Política de privacidad es la que consta en la web hefora.net y sólo los investigadores acceden a los datos, con la excepción de nombre y apellidos y email a los que puede acceder la empresa propietaria de Hefora, que no cede estos datos a terceros.

4. Respecto a las medidas de seguridad, la plataforma cumple con la GDPR, y tiene las siguientes medidas de seguridad implementadas:

- A nivel de servidor, el servidor que administra hefora.net está alojado en un centro de datos privado con tecnología en la nube sobre OpenStack. El proveedor no tiene acceso al sistema del servidor ni a sus datos. Las instantáneas realizadas se almacenan en otro centro de datos de las mismas instalaciones y su transferencia se realiza a través de una red interna aislada. La administración del servidor se realiza bajo el servicio OpenSSH con autenticación por contraseña o clave privada RSA. La comunicación entre el usuario y el servicio web se realiza bajo SSL con un certificado EV (SectigoRSA Extended Validation Secure Server CA). La comunicación entre los servicios web y la base de datos no está expuesta al exterior y se reduce a su red interna aislada administrada por los servicios del sistema Debian 9.9. Las copias de seguridad de la información se cifran a nivel del sistema bajoAES-256 y se transfieren con OpenSSH a través de la red interna bajo VLAN a otro servidor de copia de seguridad. Al igual que el servidor hefora.net, este servidor de respaldo funciona con tecnología en la nube sobre OpenStack y el proveedor tampoco tiene acceso al sistema. La administración de este servidor también se realiza bajo el servicio OpenSSH con autenticación por contraseña o clave privada RSA.

- En el nivel de la base de datos, todos los datos personales y médicos se almacenan con cifrado a nivel de registro, lo que significa que todo es incomprensible sin antes descifrarlo. Además, los registros médicos están debidamente seudoanonimizados por lo que no es posible relacionar directamente a la persona física y sus datos médicos. Además, todos los documentos intercambiados con los usuarios (ej. videos) están encriptados a través de un proveedor de seguridad externo, chino.io, que tiene la certificación ISO 27001. Para fines de procesamiento y exportación, todos los datos se anonimizan convenientemente".

- Respecto a la exportación de cuestionarios: para cada cuestionario y la persona que lo ha rellenado, lo que se hace es generar un identificador encriptado a partir del identificador original de dicha persona, de tal forma que aunque se tuviera acceso a la base de datos original, no se podría saber a qué identificador original hace referencia, y por tanto no podría saberse qué persona rellenó cada cuestionario. De esta forma, con el listado de cuestionarios exportado, puede saberse si 2 cuestionarios distintos pertenecen a la misma persona (tendrá el mismo identificador encriptado), pero no

puede saberse a qué persona física corresponde. Es decir, son anónimos.

- En cuanto al proceso concreto de seudoanonimización, lo que se ha hecho es romper el vínculo en bases de datos entre las tablas de pacientes y de datos médicos. No hay un identificador que nos indique a qué paciente corresponde cada historia clínica, son tablas independientes. En otra tabla aparte, sin relación tampoco con las anteriores, lo que se está haciendo es guardar el identificador del paciente encriptado con un algoritmo AES-256 y sus datos personales también encriptados con ese mismo algoritmo. Para conocer a qué persona física corresponde una historia clínica habría que desencriptar dicho identificador, luego no hay forma de, si se tiene acceso a la base de datos, establecer la relación entre paciente e historia clínica sin conocer la clave y algoritmo de desencriptación. Además, en la tabla de historia clínica no se guarda ningún tipo de dato personal identificativo. El resultado es que las historias clínicas están seudoanonimizadas, ya que en su información no aparece ningún tipo de dato personal ni se puede establecer relación directa con su paciente. Y lo mismo para el resto de datos que tienen relación con la historia clínica: cuestionarios, prescripciones, etc.

Así mismo, en cumplimiento de los dispuesto en el RGPD, se informa que, si así lo desea, podrá acudir a la Agencia de Protección de Datos (https://www.aepd.es) para presentar una reclamación cuando considere que no se hayan atendido debidamente sus derechos.

El tratamiento de sus datos personales se realizará utilizando técnicas para mantener su anonimato mediante el uso de códigos aleatorios, con el fin de que su identidad personal quede completamente oculta durante el proceso de investigación.

A partir de los resultados del trabajo de investigación, se podrán elaborar comunicaciones científicas para ser presentadas en congresos o revistas científicas, pero se harán siempre con datos agrupados y nunca se divulgará nada que le pueda identificar.

Los datos de las entrevistas y valoraciones presenciales se realizarán mediante la incorporación de estos resultados en papel a un programa de tratamiento de datos.

8. ¿Quién financia el estudio?

Este proyecto no tiene financiación externa.

9. ¿Se me informará de los resultados del estudio?

Usted tiene derecho a conocer los resultados del presente estudio, tanto los resultados generales como los derivados de sus datos específicos. También tiene derecho a no conocer dichos resultados si así lo desea. Por este motivo en el documento de consentimiento informado le preguntaremos qué opción prefiere. En caso de que desee conocer los resultados, el investigador le hará llegar los resultados.

10. ¿Puedo cambiar de opinión?

Su participación es totalmente voluntaria, puede decidir no participar o retirarse del estudio en cualquier momento sin tener que dar explicaciones y sin que esto repercuta en su atención sanitaria. Basta con que le manifieste su intención al investigador principal del estudio.

11. ¿Qué pasa si me surge alguna duda durante mi participación?

En la primera página de este documento está recogido el nombre y el teléfono de contacto de los investigadores responsables del estudio. Puede dirigirse a él en caso de que le surja cualquier duda sobre su participación.

Muchas gracias por su atención, si finalmente desea participar le rogamos que firme el documento de consentimiento que se adjunta.

....

ANEXO XVII: DOCUMENTO DE CONSENTIMIENTO INFORMADO

Título del PROYECTO: Eficacia de un programa de telerehabilitación asincrónico en pacientes postCOVID-19: estudio de viabilidad.

Yo, ... (nombre y apellidos del participante)

He leído la hoja de información que se me ha entregado.
He podido hacer preguntas sobre el estudio y he recibido suficiente información sobre el mismo.
He hablado con: .... (nombre y apellidos del investigador)
Comprendo que mi participación es voluntaria.
Comprendo que puedo retirarme del estudio:

1) cuando quiera
2) sin tener que dar explicaciones
3) sin que esto repercuta en mis cuidados médicos

Presto libremente mi consentimiento para participar en este estudio y doy mi consentimiento para el acceso y utilización de mis datos conforme se estipula en la hoja de información que se me ha entregado (también para que se realice el análisis genético –si procede-).
Deseo ser informado sobre los resultados del estudio: sí no (marque lo que proceda)

Si procede: Doy mi conformidad para que mis datos clínicos sean revisados por personal ajeno al centro, para los fines del estudio, y soy consciente de que este consentimiento es revocable.

He recibido una copia firmada de este Consentimiento Informado.
Firma del participante: ....
Fecha: ....

He explicado la naturaleza y el propósito del estudio al paciente mencionado
Firma del Investigador:
Fecha:

………………………………………………………………

………………………………………………………………

ANEXO XVIII: Política de protección de datos plataforma HEFORA.

POLÍTICA DE PRIVACIDAD

**Información básica sobre Protección de datos**	
**Responsable:**	FISIO CONSULTORES, S.L.
**Finalidades:**	1. Poder ofrecerle los servicios y utilidades que presenta nuestra plataforma HEFORA. Así como, informarle sobre nuestros servicios y/o productos y realizar comunicaciones comerciales de su interés.<br><br>2. Realizar una investigación científica y de estudios estadísticos.
**Legitimación:**	- Ejecución de un contrato (uso de la plataforma)<br>- Consentimiento del interesado (tratamiento de datos de salud e investigación científica y estudios estadísticos).
**Destinatarios:**	Las únicas cesiones previstas son:<br>- La realizada en el momento de contratar los servicios de un profesional de la plataforma ya que tus datos existentes en la plataforma pasarán a ser visibles y responsabilidad del profesional para una finalidad diferente como es la de prestarte el servicio de asistencia sanitaria.<br><br>- Las que se realicen por obligación legal.<br><br>Existen Encargados de Tratamiento fuera de la Unión Europea, acogido a “Privacy

	Shield".
**Derechos:**	Tiene derecho a acceder, rectificar y suprimir los datos, así como otros derechos, indicados en la información adicional, que puede ejercer en info@hefora.com
**Procedencia:**	Del propio interesado o de su representante legal.
**Información adicional:**	Puede consultar información adicional y detallada sobre Protección de Datos en nuestra página web: https://hefora.com/wp-content/uploads/2018/08/Politica_de_PRIVACIDAD.pdf

1. INFORMACIÓN AL USUARIO

FISIO CONSULTORES, S.L., en adelante "Responsable" o "Servicio", y como sociedad mercantil española con domicilio social en la Calle Magallón, 12 – 50420 de Cadrete (Zaragoza) y NIF: B-99396947 es el Responsable, o Encargado cuando así corresponda en función de su perfil, del tratamiento de los datos personales de los usuarios de la plataforma y le informa que estos datos serán tratados de conformidad con lo dispuesto en el Reglamento (UE) 2016/679 de 27 de abril de 2016 (GDPR) relativo a la protección de las personas físicas en lo que respecta al tratamiento de datos personales y a la libre circulación de estos datos, por lo que se le facilita la siguiente información del tratamiento:

**PERFIL Y DATOS**	**HEFORA trata sus datos como:**
**Profesional** (datos identificativos, profesionales, de facturación, entre otros)	**Responsable del Tratamiento**

**Paciente invitado por Profesional** (datos como paciente del profesional y de registro en la plataforma, además de los relativos a salud)	**Encargado del Tratamiento**
**Paciente potencial** (datos de registro y perfil de plataforma. Incluyendo posibles datos de salud que introduzcas)	**Responsable del Tratamiento**

Finalidades del tratamiento:

1.- ***Mantener una relación contractual con el Usuario a través de la plataforma para poder prestarle el servicio***. Las operaciones previstas para realizar el tratamiento son:

· La recabación y almacenamiento de los datos personales para la prestación de los servicios descritos en la plataforma. Para que pueda gestionar su perfil, llevar un control y recibir servicios sanitarios de fisioterapia con la ayuda de la plataforma. Pudiendo ser tal tratamiento llevado a cabo por parte del Responsable en calidad de tal o de Encargado de tratamiento según el perfil de usuario afectado.

· Tramitar encargos, solicitudes o cualquier tipo de petición que sea realizada por el usuario a través de cualquiera de las formas de contacto que se ponen a su disposición.

2.- ***La remisión de comunicaciones comerciales publicitarias vía correo electrónico, comunidades sociales o cualquier otro medio electrónico o físico, presente o futuro, que posibilite realizar comunicaciones comerciales***. Las operaciones previstas para realizar el tratamiento son:

· Las mismas establecidas para el uso de la plataforma. Estas comunicaciones serán realizadas por el RESPONSABLE y relacionadas sobre sus productos y/o servicios, o de sus colaboradores o proveedores con los que éste haya alcanzado algún acuerdo de promoción. En este caso, los terceros nunca tendrán acceso a

los datos personales de los usuarios de la plataforma.

.

Y, de existir, la remisión del boletín de noticias de la página web.

3.- ***La realización de una investigación científica y de estudios estadísticos tras realizar y aplicar las correspondientes técnicas de anonimización de los datos personales siendo estas irreversibles.***

Bases jurídicas de los distintos tratamientos: Según proceda,

· El contrato con el interesado a través de los Términos y Condiciones de Uso de la plataforma y de la presente Política de Privacidad para disfrutar de los servicios de la plataforma.

· El interés legítimo del Responsable, para el envío de comunicaciones comerciales.

· El consentimiento del interesado, para el tratamiento de datos de salud e investigación científica y estudios estadísticos.

Criterios de conservación de los datos: se conservarán mientras permanezca vigente el contrato y/o exista un interés mutuo para mantener el fin del tratamiento. Cuando ya no sea necesario para tal fin, se suprimirán con medidas de seguridad adecuadas para garantizar la pseudonimización y/o anonimización de los datos o la destrucción total de los mismos.

Comunicación de los datos: No vendemos a otras empresas tus datos personales. Las únicas cesiones previstas son:

- La realizada en el momento en el que una vez registrado en la plataforma decide contratar los servicios de un profesional de la misma, ya que sus datos existentes en la plataforma pasarán a ser visibles y responsabilidad del profesional para una finalidad diferente a la del acceso a la plataforma, como es la de prestarle el servicio de asistencia sanitaria correspondiente.
- Las que se realicen por obligación legal.

Existen Encargados de Tratamiento fuera de la Unión Europea, acogido a

“PrivacyShield”.

Derechos que asisten al Usuario:

- Derecho a retirar el consentimiento en cualquier momento.
- Derecho de acceso, rectificación, portabilidad y supresión de sus datos y a la limitación u oposición a su tratamiento.
- Derecho a presentar una reclamación ante la autoridad de control (www.agpd.es) si considera que el tratamiento no se ajusta a la normativa vigente.

Datos de contacto para ejercer sus derechos:

Puedes ejercer en cualquier momento los derechos de acceso, rectificación, supresión, limitación de tu tratamiento, oposición y portabilidad de tus datos de carácter personal mediante correo electrónico dirigido a: info@hefora.com O la dirección postal: FISIO CONSULTORES, S.L. Calle Magallón, 12 - 50420 Cadrete (ZARAGOZA).

En ambos casos deberás identificarte con tu nombre y apellidos, además de una copia de tu DNI.

En el caso de que hayas otorgado el consentimiento para alguna finalidad específica, tienes derecho a retirar el consentimiento en cualquier momento, sin que ello afecte a la licitud del tratamiento basado en el consentimiento previo a su retirada.

Además, si como usuario de la plataforma consideras que hay un problema con la forma en que el Responsable está manejando tus datos, puedes dirigir tus reclamaciones al DPD o la autoridad de protección de datos que corresponda, siendo la Agencia Española de Protección de Datos la indicada en el caso de España.

El presente documento presenta las condiciones de recopilación, tratamiento y uso de la información personal y no personal de los usuarios.

2. INFORMACIÓN FACILITADA POR EL USUARIO Y SU CARÁCTER OBLIGATORIO O FACULTATIVO

Los Usuarios, mediante la marcación de las casillas correspondientes y entrada de datos en los campos, marcados con un asterisco (*) en el formulario de contacto o presentados en formularios de descarga, aceptan expresamente y de forma libre e inequívoca, que sus datos son necesarios para atender su petición, por parte del prestador, siendo voluntaria la inclusión de datos en los campos restantes. El Usuario garantiza que los datos personales facilitados al RESPONSABLE son veraces y se hace responsable de comunicar cualquier modificación de los mismos.

Cumplimentar los formularios es voluntario y, si no se completan los campos obligatorios, posiblemente algunas de las funciones de la plataforma no se ejecutarán o lo harán de forma limitada.

El RESPONSABLE informa y garantiza expresamente a los usuarios que sus datos personales no serán cedidos en ningún caso a terceros, y que siempre que realizará algún tipo de cesión de datos personales, se pedirá previamente el consentimiento expreso, informado e inequívoco por parte los Usuarios.

Los datos personales que recogeremos dependerán en función del uso que se haga de la plataforma.

Recopilaremos estos datos de manera automática –mediante cookies o similares, con tu consentimiento. Visita nuestra **Política de Cookies**; la dirección IP de tu conexión y datos sobre tu dispositivo y conexión, entre otros-, los que nos proporciones voluntariamente –a través del registro en la plataforma, uso, mensajes enviados, suscripción a newsletter, entre otros- y los que nos proporcionen terceros –como redes sociales-.

3. MEDIDAS DE SEGURIDAD

Que de conformidad con lo dispuesto en las normativas vigentes en protección de datos personales, el RESPONSABLE está cumpliendo con todas las disposiciones

de las normativas GDPR para el tratamiento de los datos personales de su responsabilidad, y manifiestamente con los principios descritos

en el artículo 5 del GDPR, por los cuales son tratados de manera lícita, leal y transparente en relación con el interesado y adecuados, pertinentes y limitados a lo necesario en relación con los fines para los que son tratados.

El RESPONSABLE garantiza que ha implementado políticas técnicas y organizativas apropiadas para aplicar las medidas de seguridad que establecen el GDPR con el fin de proteger los derechos y libertades de los Usuarios y les ha comunicado la información adecuada para que puedan ejercerlos.

4.- CAMBIOS EN LA POLÍTICA DE PRIVACIDAD

Podremos actualizar esta Política de Privacidad en el futuro. Te informaremos sobre sus cambios enviando un aviso a la dirección de correo electrónico facilitada y/o colocando un aviso en un lugar prominente de nuestra web.

5.- CONTACTO

Si tienes dudas sobre esta Política de Privacidad, contacta con nosotros en:

Correo electrónico: **info@hefora.com**

Dirección: FISIO CONSULTORES, S.L.. Calle Magallón, 12 - 50420 Cadrete (ZARAGOZA).

8.- BIBLIOGRAFÍA

1. Huang C, Wang Y, Li X, Ren L, Zhao J, Hu Y, et al. Clinical features of patients infected with 2019 novel coronavirus in Wuhan, China. Lancet. 2020;395(10223):497-506.
2. Sanidad Md. Enfermedad por coronavirus, COVID-19. Actualización, 12 noviembre, 2020.2020:[101 p.].
3. Cucinotta D, Vanelli M. WHO Declares COVID-19 a Pandemic. Acta Biomed. 2020;91(1):157-60.
4. SEDISA. ara la atención integral al paciente post-COVID:[28 p.].
5. Kim SY, Kumble S, Patel B, Pruski AD, Azola A, Tatini AL, et al. Managing the Rehabilitation Wave: Rehabilitation Services for COVID-19 Survivors. Arch Phys Med Rehabil. 2020.
6. Aragón. Gd. Información coronavirus COVID-19 Aragón. 2020 [Available from: https://www.aragon.es/coronavirus.
7. Cipollaro L, Giordano L, Padulo J, Oliva F, Maffulli N. Musculoskeletal symptoms in SARS-CoV-2 (COVID-19) patients. J Orthop Surg Res. 152020. p. 178.
8. Wang D, Hu B, Hu C, Zhu F, Liu X, Zhang J, et al. Clinical Characteristics of 138 Hospitalized Patients With 2019 Novel Coronavirus-Infected Pneumonia in Wuhan, China. Jama. 2020;323(11):1061-9.
9. Guan W-j, Ni Z-y, Hu Y, Liang W-h, Ou C-q, He J-x, et al. Clinical characteristics of 2019 novel coronavirus infection in China. medRxiv. 2020:2020.02.06.20020974.
10. Wu Z, McGoogan JM. Characteristics of and Important Lessons From the Coronavirus Disease 2019 (COVID-19) Outbreak in China: Summary of a Report of 72 314 Cases From the Chinese Center for Disease Control and Prevention. Jama. 2020;323(13):1239-42.
11. Laxe S, Miangolarra Page JC, Chaler J, Gil Fraguas L, Gómez A, Luna F, et al. Rehabilitation in the time of COVID-19. Rehabilitacion. 2020;54(3):149-53.
12. Bury TJ, Stokes EK. On "Musculoskeletal Physical Therapy During the COVID-19 Pandemic: Is Telerehabilitation the Answer?" Turolla A, Rossettini G, Viceconti A, Palese A, Geri T. Phys Ther. 2020;100:1260-1264. Phys Ther. 1002020. p. 1883.
13. Thomas P, Baldwin C, Bissett B, Boden I, Gosselink R, Granger CL, et al. Physiotherapy management for COVID-19 in the acute hospital setting: clinical practice recommendations. J Physiother. 2020;66(2):73-82.

14. Duan L, Zhu G. Psychological interventions for people affected by the COVID-19 epidemic. Lancet Psychiatry. 2020;7(4):300-2.

15. Gonzalez-Gerez JJ, Bernal-Utrera C, Anarte-Lazo E, Garcia-Vidal JA, Botella-Rico JM, Rodriguez-Blanco C. Therapeutic pulmonary telerehabilitation protocol for patients affected by COVID-19, confined to their homes: study protocol for a randomized controlled trial. Trials. 2020;21(1):588.
16. Vasconcello-Castillo L, Torres-Castro R, Solís-Navarro L, Rivera G, Puppo H. Evaluación Funcional y Respiratoria en Pacientes post COVID-19: ¿Cuáles son las mejores pruebas? 2020:109-15.
17. Bowden Davies KA, Pickles S, Sprung VS, Kemp GJ, Alam U, Moore DR, et al. Reduced physical activity in young and older adults: metabolic and musculoskeletal implications. Ther Adv Endocrinol Metab. 2019;10:2042018819888824.
18. de Sire A, Andrenelli E, Negrini F, Negrini S, Ceravolo MG. Systematic rapid living review on rehabilitation needs due to COVID-19: update as of April 30th, 2020. Eur J Phys Rehabil Med. 2020;56(3):354-60.
19. Ceravolo MG, de Sire A, Andrenelli E, Negrini F, Negrini S. Systematic rapid "living" review on rehabilitation needs due to COVID-19: update to March 31st, 2020. Eur J Phys Rehabil Med. 2020;56(3):347-53.
20. Curci C, Pisano F, Bonacci E, Camozzi DM, Ceravolo C, Bergonzi R, et al. Early rehabilitation in post-acute COVID-19 patients: data from an Italian COVID-19 Rehabilitation Unit and proposal of a treatment protocol. Eur J Phys Rehabil Med. 2020;56(5):633-41.
21. Richmond T, Peterson C, Cason J, Billings M, Terrell EA, Lee ACW, et al. American Telemedicine Association's Principles for Delivering Telerehabilitation Services. Int J Telerehabil. 2017;9(2):63-8.
22. Cottrell MA, Russell TG. Telehealth for musculoskeletal physiotherapy. Musculoskelet Sci Pract. 2020;48:102193.
23. Barbosa MT, Sousa CS, Morais-Almeida M, Simões MJ, Mendes P. Telemedicine in COPD: An Overview by Topics. Copd. 2020;17(5):601-17.
24. Piotrowicz E PROGOPPSGRSDIRPMKIKTMABM, Zareba W. Hybrid telerehabilitation and telecare is more efffective than usual care in improving peak vo2 and 6-minute walk distance but is not associated with reduction of 24-months mortality except in the most experienced center -result of the first randomized, p. Circulation. 2019;140.

25. Piotrowicz E PROGPMBM, Zaręba W. Hybrid comprehensive telerehabilitation in heart failure patients (TELEREH-HF): a randomized, multicenter, prospective, open-label, parallel group controlled trial-Study design and description of the intervention. American heart journal. 2019;217:148-58.
26. Thompson DA, Leimig R, Gower G, Winsett RP. Assessment of depressive symptoms during post-transplant follow-up care performed via telehealth. Telemed J E Health. 2009;15(7):700-6.
27. Agostini M, Moja L, Banzi R, Pistotti V, Tonin P, Venneri A, et al. Telerehabilitation and recovery of motor function: a systematic review and meta-analysis. Journal of telemedicine and telecare. 2015;21(4):202-13.
28. Grona SL, Bath B, Busch A, Rotter T, Trask C, Harrison E. Use of videoconferencing for physical therapy in people with musculoskeletal conditions: A systematic review. J Telemed Telecare. 2018;24(5):341-55.
29. Bhaskar S, Bradley S, Chattu VK, Adisesh A, Nurtazina A, Kyrykbayeva S, et al. Telemedicine as the New Outpatient Clinic Gone Digital: Position Paper From the Pandemic Health System REsilience PROGRAM (REPROGRAM) International Consortium (Part 2). Front Public Health. 2020;8:410.
30. Bennell KL, Marshall CJ, Dobson F, Kasza J, Lonsdale C, Hinman RS. Does a Web-Based Exercise Programming System Improve Home Exercise Adherence for People With Musculoskeletal Conditions?: A Randomized Controlled Trial. Am J Phys Med Rehabil. 2019;98(10):850-8.
31. Claes J BRWCBAGCAMMNMKMNCIWDBWFD, Cornelissen VA. PATHway I: design and rationale for the investigation of the feasibility, clinical effectiveness and cost-effectiveness of a technology-enabled cardiac rehabilitation platform. BMJ open. 2017;7(6):e016781-e.
32. Hosseiniravandi M, Kahlaee AH, Karim H, Ghamkhar L, Safdari R. Home-based telerehabilitation software systems for remote supervising: a systematic review. International journal of technology assessment in health care. 2020;36(2):113-25.
33. Daher A, Balfanz P, Cornelissen C, Müller A, Bergs I, Marx N, et al. Follow up of patients with severe coronavirus disease 2019 (COVID-19): Pulmonary and extrapulmonary disease sequelae. Respir Med. 2020;174:106197.

34. Sanidad Md. Manejo clínico del COVID-19: atención hospitalaria.2020 20/12/2020:[28 p.]. Available from: https://www.mscbs.gob.es/profesionales/saludPublica/ccayes/alertasActual/nCov/documentos/Protocolo_manejo_clinico_ah_COVID-19.pdf.
35. Lobo A, Saz P, Marcos G, Día JL, de la Cámara C, Ventura T, et al. [Revalidation and standardization of the cognition mini-exam (first Spanish version of the Mini-Mental Status Examination) in the general geriatric population]. Med Clin (Barc). 1999;112(20):767-74.
36. Aragón. CPdFd. Guía de intervención del Fisioterpeuta en la Atención hospitalaria del paciente infectado por el nuevo coronavirus (SARS-COOV-2)2020.
37. Madrid. CPdFdlCd. Recomendaciones de Fisioterapia Respiratoria y Ejercicio Terapéutico para personas confinadas en fase de recuperación por COVID-19. 2020.
38. Greenhalgh T, Javid B, Knight M, Inada-Kin M. What is the efficacy and safety of rapid exercise test for extertional desaturation in Covid-19: a rapid systematic review.: University of Oxford; 2020.
39. Learmonth YC, Dlugonski D, Pilutti LA, Sandroff BM, Klaren R, Motl RW. Psychometric properties of the Fatigue Severity Scale and the Modified Fatigue Impact Scale. J Neurol Sci. 2013;331(1-2):102-7.
40. Bernal-Vargas L, Riveros-Munévar F, Vinaccia-Alpi S, Quiceno-Sierra J-M. Estructura factorial y consistencia interna de la Escala de severidad de fatiga en población colombiana con enfermedades crónicas. Enfermería Global. 2017;16:37-49.
41. Duarte RE, Velasco E, Sánchez-Sosa JJ, Reyes-Lagunes LI. Validación psicométrica de la Escala de gravedad de la fatiga en médicos residentes mexicanos.: Educación Médica; 2019. p. 28-36.
42. Hewlett S, Dures E, Almeida C. Measures of fatigue: Bristol Rheumatoid Arthritis Fatigue Multi-Dimensional Questionnaire (BRAF MDQ), Bristol Rheumatoid Arthritis Fatigue Numerical Rating Scales (BRAF NRS) for severity, effect, and coping, Chalder Fatigue Questionnaire (CFQ), Checklist Individual Strength (CIS20R and CIS8R), Fatigue Severity Scale (FSS), Functional Assessment Chronic Illness Therapy (Fatigue) (FACIT-F), Multi-Dimensional Assessment of Fatigue (MAF), Multi-Dimensional Fatigue Inventory (MFI), Pediatric Quality Of Life (PedsQL) Multi-Dimensional Fatigue Scale,

Profile of Fatigue (ProF), Short Form 36 Vitality Subscale (SF-36 VT), and Visual Analog Scales (VAS). Arthritis Care Res (Hoboken). 2011;63 Suppl 11:S263-86.

43. Stone P, Richards M, A'Hern R, Hardy J. Fatigue in patients with cancers of the breast or prostate undergoing radical radiotherapy. J Pain Symptom Manage. 2001;22(6):1007-15.
44. Schneeberger EE, Marengo MF, Dal Pra F, Maldonado Cocco JA, Citera G. Fatigue assessment and its impact in the quality of life of patients with ankylosing spondylitis. Clin Rheumatol. 2015;34(3):497-501.
45. Pennisi M, Malaguarnera G, Di Bartolo G, Lanza G, Bella R, Chisari EM, et al. Decrease in Serum Vitamin D Level of Older Patients with Fatigue. Nutrients. 2019;11(10).
46. Gimeno-Santos E, Arbillaga-Etxarri A, Vilaro J, Balañá A, Barberan-Garcia A, del Corral T, et al. Reference equations for 6-minute walk test in Spanish population2015. PA1543 p.
47. Rikli RE, Jones CJ. Development and validation of criterion-referenced clinically relevant fitness standards for maintaining physical independence in later years. Gerontologist. 2013;53(2):255-67.
48. Cobo-Mejía EA, Ochoa González ME, Y. RCL, Vargas Niño DM, Sáenz Pacheco AM, Sandoval-Cuellar C. Confiabilidad del Senior Fitness Test versión en español, para población adulta mayor en Tunja-Colombia.: Arch Med Deporte; 2016. p. 382-6.
49. Cuellar CS, Mejía EAC, Ochoa ME, Niño DMV, Castillo LYR, González LMB, et al. VALIDACIÓN DEL SENIOR FITNESS TEST EN LA CIUDAD DE TUNJA. Expomotricidad. 2015.
50. Boneth Collantes M, Ariza García CL, Angarita Fonseca A, Parra Patiño J, Monsalve A, Gómez E. Reproducibilidad de las pruebas Arm Curly Chair Stand para evaluar resistencia muscular en población adulta mayor. Revista Ciencias de la Salud. 2012;10:179-93.
51. Vaidya T, Chambellan A, de Bisschop C. Sit-to-stand tests for COPD: A literature review. Respir Med. 2017;128:70-7.
52. Houchen-Wolloff L, Daynes E, Watt A, Chaplin E, Gardiner N, Singh S. Which functional outcome measures can we use as a surrogate for exercise capacity during remote cardiopulmonary rehabilitation assessments? A rapid narrative review. ERJ Open Res. 2020;6(4).

53. Langhammer B, Stanghelle JK. The Senior Fitness Test. Journal of Physiotherapy. 2015;61(3):163.

54. McAllister LS, Palombaro KM. Modified 30-Second Sit-to-Stand Test: Reliability and Validity in Older Adults Unable to Complete Traditional Sit-to-Stand Testing. J Geriatr Phys Ther. 2020;43(3):153-8.

55. Klok FA, Boon G, Barco S, Endres M, Geelhoed JJM, Knauss S, et al. The Post-COVID-19 Functional Status scale: a tool to measure functional status over time after COVID-19. Eur Respir J. 562020.

56. Sherer M, Maddux JE, Mercandante B, Prentice-Dunn S, Jacobs B, Rogers RW. The Self-Efficacy Scale: Construction and Validation. Psychological Reports. 1982;51(2):663-71.

57. Bosscher RJ, Smit JH. Confirmatory factor analysis of the General Self-Efficacy Scale. Behaviour Research and Therapy. 1998;36(3):339-43.

58. López-Torrecillas F, García J, Cañadas GA, Uclés IR, de la Fuente EI. Validity of Self-Efficacy Scale Scores for a Spanish Sample. Psychological Reports. 2006;98(2):437-50.

59. Herrero R, Espinoza M, Molinari G, Etchemendy E, Garcia-Palacios A, Botella C, et al. Psychometric properties of the General Self Efficacy-12 Scale in Spanish: General and clinical population samples. Comprehensive Psychiatry. 2014;55(7):1738-43.

60. Sanjuan Suarez P, Pérez García AM, Bermúdez Moreno J. Escala de Autoeficacia general:datos psicométricos de la adaptación para población española.: Psicothema; 2000. p. 509-13.

61. Antony MM, Bieling PJ, Cox BJ, Enns MW, Swinson RP. Psychometric properties of the 42-item and 21-item versions of the Depression Anxiety Stress Scales in clinical groups and a community sample. Psychological Assessment. 1998;10(2):176-81.

62. Lovibond PF, Lovibond SH. The structure of negative emotional states: comparison of the Depression Anxiety Stress Scales (DASS) with the Beck Depression and Anxiety Inventories. Behav Res Ther. 1995;33(3):335-43.

63. Daza P, Novy DM, Stanley MA, Averill P. The Depression Anxiety Stress Scale-21: Spanish Translation and Validation with a Hispanic Sample. Journal of Psychopathology and Behavioral Assessment. 2002;24(3):195-205.

64. Ruiz FJ, García Martín MB, Suárez Falcón JC, Odriozola González P. The Hierarchical Factor Structure of the Spanish Version of Depression Anxiety and Stress

Scale -21. International Journal of Psychology and Psychological Therapy. 2017;17(1):97-105.

65. Moreno-Murcia JA, Marzo JC, Martinez-Galindo C, Conte Marin L. Validación de le Escala de "Satisfacción de las Necesidades Psicológicas Básicas" y del Cuestionario de la "Regulación Conductual en el Deporte" al contexto español. Revista internacional de ciencias del deporte. 2011;26:355-69.
66. da Cruz MMA, Ricci-Vitor AL, Borges GLB, da Silva PF, Turri-Silva N, Takahashi C, et al. A Randomized Controlled Crossover Trial of Virtual Reality in Maintenance Cardiovascular Rehabilitation in a Low-Resource Setting: Impact on Adherence, Motivation, and Engagement. Phys Ther. 2021.
67. Guddal MH, Stensland SO, Smastuen MC, Johnsen MB, Zwart JA, Storheim K. Physical Activity Level and Sport Participation in Relation to Musculoskeletal Pain in a Population-Based Study of Adolescents: The Young-HUNT Study. Orthop J Sports Med. 2017;5(1):2325967116685543.
68. Landeta O, Calvete E. Adaptación y validación de la Escala Multidimensional de Apoyo Social Percibido. Ansiedad y Estrés. 2002; 8(2-3): 173-182.
69. Granero M, Perman G, Vazquez Peña F, Barbaro CA, Zozaya ME, Martinez Infantino VS, et al. [Validation of the Spanish version of the Lubben Social Network Scale-6]. Rev Fac Cien Med Univ Nac Cordoba. 2020;77(4):296-300.
70. Vilagut G, Valderas JM, Ferrer M, Garin O, López-García E, Alonso J. [Interpretation of SF-36 and SF-12 questionnaires in Spain: physical and mental components]. Med Clin (Barc). 2008;130(19):726-35.
71. Vilagut G, Ferrer M, Rajmil L, Rebollo P, Permanyer-Miralda G, Quintana JM, et al. [The Spanish version of the Short Form 36 Health Survey: a decade of experience and new developments]. Gac Sanit. 2005;19(2):135-50.
72. Guerra-Tapia A, Buendía-Eisman A, Ferrando Barbera J. Final Phase in the Validation of the Cross-Cultural Adaptation of the Hair-Specific Skindex-29 Questionnaire Into Spanish: Sensitivity to Change and Correlation With the 12-Item Short-Form Health Survey. Actas Dermosifiliogr. 2019;110(10):819-29.
