# Supplementary material for: Efficacy of an asynchronous telerehabilitation program in post-COVID-19 patients: A protocol for a pilot randomized controlled trial
Source: PLoS One. 2022 Jul 19;17(7):e0270766. doi: 10.1371/journal.pone.0270766 (PMC9295945; doi:10.1371/journal.pone.0270766)
Supplement: S3 File — (PDF) [file pone.0270766.s004.pdf]

Report Favourable Opinion

GOBIERNO DE ARAGON
Departamento de Sanidad

C.P. - C.I. PI21/019
24 February 2021

Ms. María González Hinjos, Secretary of CEIC Aragón (CEICA)

CERTIFIES

That the CEIC Aragón (CEICA) in its meeting of 24/02/2021, Act Nº 04/2021 has evaluated the investigator's proposal referred to the study**:**

Title: Efficacy of an asynchronous telerehabilitation programme in post-COVID-19 patients: feasibility study.

Principal Investigators: Sandra Calvo Carrión and Carolina Jiménez Sánchez

Protocol version: V 23/02/20214
Information and consent document version: V 4 23/02/2021 2º.

Considers that

- The project is planned in accordance with the requirements of Law 14/2007, of 3July, on Biomedical Research, and its implementation is relevant.
- The necessary requirements for the appropriateness of the protocol in relation to the objectives of the study are met and the foreseeable risks and discomfort to the subject are justified.
- The use of the data and the documents drawn up for obtaining consent is appropriate.
- The extent of the financial compensation provided for does not interfere with respect for ethical principles.
- The capacity of the Investigators and the means available are appropriate to carry out the study.

3º. Therefore, this CEIC issues a **FAVOURABLE OPINION to carry out the study**.

What I sign in Zaragoza

GONZALEZ HINJOS MARIA - DNI 03857456B

Digitally signed by GONZALEZ HINJOS MARIA - DNI 03857456B
Date: 2021.02.26 09:50:14 +01'00'

María González Hinjos
Secretary of CEIC Aragón (CEICA)

Tel. 976 71 5836 Fax. 976 71 55 54 E-mail: mgonzalezh.ceic@aragon.es

Report Favourable Opinion

C.P. - C.I. PI21/019

21 April 2021

Ms. María González Hinjos, Secretary of CEIC Aragón (CEICA)

CERTIFIES

That the CEIC Aragón (CEICA) in its meeting of 21/04/2021, Act Nº 08/2021 has evaluated the proposal of relevant modification referred to the study**:**

Title: Efficacy of an asynchronous telerehabilitation programme in post-COVID-19 patients: feasibility study.

Principal Investigators: Sandra Calvo Carrión and Carolina Jiménez Sánchez

2nd. This amendment proposes:

- Inclusion of a new centre: Royo Villanova Hospital.
- Modification of the research team
- Protocol version: V5 of 12/04/2021
- Information and Consent Document Version: V5 of 12/04/2021

3º. Considers that

- The project is planned in accordance with the requirements of Law 14/2007, of 3July, on Biomedical Research, and its implementation is relevant.
- The necessary requirements for the appropriateness of the protocol in relation to the objectives of the study are met and the foreseeable risks and discomfort to the subject are justified.
- The use of the data and the documents drawn up for obtaining consent is appropriate.
- The extent of the financial compensation provided for does not interfere with respect for ethical principles.
- The capacity of the Investigators and the means available are appropriate to carry out the study.

4º. Therefore, this CEIC issues a **FAVOURABLE OPINION to carry out the study and the modifications requested.**

What I sign in Zaragoza

GONZALEZ HINJOS MARIA - DNI 03857456B

Digitally signed by GONZALEZ HINJOS MARIA - DNI 03857456B Date: 2021.04.26 13:25:49 +02'00'

María González Hinjos
Secretary of CEIC Aragón (CEICA)

Tel. 976 71 5836 Fax. 976 71 55 54 Email: mgonzalezh.ceic@aragon.es
