# Supplementary material for: Efficacy of an asynchronous telerehabilitation program in post-COVID-19 patients: A protocol for a pilot randomized controlled trial
Source: PLoS One. 2022 Jul 19;17(7):e0270766. doi: 10.1371/journal.pone.0270766 (PMC9295945; doi:10.1371/journal.pone.0270766)
Supplement: S4 File — (PDF) [file pone.0270766.s005.pdf]

C.P. - C.I. PI21/019

24 de febrero de 2021

Dña. María González Hinjos, Secretaria del CEIC Aragón (CEICA)

**CERTIFICA**

**1º.** Que el CEIC Aragón (CEICA) en su reunión del día 24/02/2021, Acta Nº 04/2021 ha evaluado la propuesta del investigador referida al estudio:

**Título: Eficacia de un programa de telerehabilitación asincrónico en pacientes postCOVID-19: estudio de viabilidad.**

**Investigadoras Principales: Sandra Calvo Carrión y Carolina Jiménez Sánchez**

**Versión protocolo: V 4 23/02/2021**
**Versión documento de información y consentimiento: V 4 23/02/2021**

**2º.** Considera que

- El proyecto se plantea siguiendo los requisitos de la Ley 14/2007, de 3 de julio, de Investigación Biomédica y su realización es pertinente.
- Se cumplen los requisitos necesarios de idoneidad del protocolo en relación con los objetivos del estudio y están justificados los riesgos y molestias previsibles para el sujeto.
- Es adecuada la utilización de los datos y los documentos elaborados para la obtención del consentimiento.
- El alcance de las compensaciones económicas previstas no interfiere con el respeto a los postulados éticos.
- La capacidad de los Investigadores y los medios disponibles son apropiados para llevar a cabo el estudio.

**3º.** Por lo que este CEIC emite **DICTAMEN FAVORABLE a la realización del estudio**.

Lo que firmo en Zaragoza

María González Hinjos
Secretaria del CEIC Aragón (CEICA)



Tel. 976 71 5836 Fax. 976 71 55 54 Correo electrónico mgonzalezh.ceic@aragon.es

GOBIERNO DE ARAGON
Departamento de Sanidad

C.P. - C.I. PI21/019

21 de abril de 2021

Dña. María González Hinjos, Secretaria del CEIC Aragón (CEICA)

**CERTIFICA**

**1º.** Que el CEIC Aragón (CEICA) en su reunión del día 21/04/2021, Acta Nº 08/2021 ha evaluado la propuesta de modificación relevante referida al estudio:

**Título: Eficacia de un programa de telerehabilitación asincrónico en pacientes postCOVID-19: estudio de viabilidad.**

**Investigadoras Principales: Sandra Calvo Carrión y Carolina Jiménez Sánchez**

**2º.** Dicha modificación propone:

- Inclusión de un nuevo centro: Hospital Royo Villanova
- Modificación del equipo investigador
- Versión protocolo: V5 de 12/04/2021
- Versión documento de información y consentimiento: V5 de 12/04/2021

**3º.** Considera que

- El proyecto se plantea siguiendo los requisitos de la Ley 14/2007, de 3 de julio, de Investigación Biomédica y su realización es pertinente.
- Se cumplen los requisitos necesarios de idoneidad del protocolo en relación con los objetivos del estudio y están justificados los riesgos y molestias previsibles para el sujeto.
- Es adecuada la utilización de los datos y los documentos elaborados para la obtención del consentimiento.
- El alcance de las compensaciones económicas previstas no interfiere con el respeto a los postulados éticos.
- La capacidad de los Investigadores y los medios disponibles son apropiados para llevar a cabo el estudio.

**4º.** Por lo que este CEIC emite **DICTAMEN FAVORABLE a la realización del estudio y las modificaciones solicitadas**.

Lo que firmo en Zaragoza

María González Hinjos
Secretaria del CEIC Aragón (CEICA)



Tel. 976 71 5836 Fax. 976 71 55 54 Correo electrónico mgonzalezh.ceic@aragon.es
