# Supplementary material for: Efficacy of an asynchronous telerehabilitation program in post-COVID-19 patients: A protocol for a pilot randomized controlled trial
Source: PLoS One. 2022 Jul 19;17(7):e0270766. doi: 10.1371/journal.pone.0270766 (PMC9295945; doi:10.1371/journal.pone.0270766)
Supplement: S1 Appendix — (DOCX) [file pone.0270766.s006.docx]

**Therapeutic exercise program.**

*All exercises are accompanied by pictures or videos and a detailed explanation of the execution.*

**A: Lung capacity rehabilitation.** Two sets of 10 repetitions. For all levels. Three-five days a week.

1.- Deep inspiration followed by apnea for three to five seconds and exhalation with pursed lips.

2.- Deep inhalation by raising the arms and drawing air into the rib cage, followed by exhalation by lowering the arms.

**B: Cardiovascular therapeutic exercise.** Walking can be replaced by cycling. 3 days a week.

Level 1: walking for a minimum of 10 minutes with breaks as needed and increasing the time to 30 minutes without interruption with a fatigue level between 5 and 6 on the Borg Scale.

Level 2: light walking for a minimum of 10 minutes and increasing the time to 30 minutes without interruption with a fatigue level between 6 and 7 on the Borg Scale.

Level 3: alternate jogging with light walking for a minimum of 10 minutes and increasing the time to 30 minutes without interruption with a fatigue level between 6 and 7 on the Borg Scale.

**C: Therapeutic strength training**. Three days a week, very progressive. Control pain the day after, not to reach more than 3 for post-exercise pain on the EVA Scale. If this occurs, increase rest days. Three sets of as many repetitions as possible while maintaining recommended muscle fatigue.

Level 1: Squat with handstand, wall push-ups or arm curl with weights, sit-ups with raided shoulders.

Level 2: Wall squat, push-ups to the floor supporting knees or wall push-ups with more inclination, get down on the ground and stand up.

Level 3: squat, push-ups to the floor or arm curl with more weight, swing with weights.

**Therapeutic education.** For all levels

**1.- Education for adaptation to the lockdown**

- Use the time to do those things I had forgotten to do.

- Discover new hobbies and interests that you can do at home.

- Organise your day-to-day life well by maintaining stable routines and fixed schedules.

- Set aside time to do some physical activity every day.

- It is a good idea to keep yourself informed to stay connected with what is going on around you.

**2.- Therapeutic education: medical advice, advice on prevention of reinfection and contagion, advice on self-care: hydration, nutrition, active life**

*1.- Health*

- Monitor your symptoms. If you develop a fever, a high pulse rate, excessive fatigue or choking sensation, paralysis or any other symptoms that you think are important, telephone your health center.

*2.- Prevention*

- Keep the house ventilated.

- Wash hands frequently.

*3.-Self-care*

- Drink plenty of water and make sure you stay well hydrated.

- Eat a balanced diet with fruits and vegetables and increase protein intake (fish, meat, eggs) while decreasing foods high in sugar and fat.

- Take time each day to do breathing, relaxation and movement exercises for all parts of the body.

- Try to get some sun each day, even if it is near a window.

**3.- Emotional education.**

- Focus on the things you can do.

- Set realistic, short-term goals.

- Keep in touch with your family, neighbors or friends, even if only by phone.

- Every night think of something good that happened to you that day.

- Be patient if the after-effects of COVID-19 improve slowly, not everyone progresses at the same rate.
